# A pegivirus associated with encephalitis in red-legged partridges shows neurotropism across avian species

**DOI:** 10.1038/s41467-026-73858-8

**Published:** 2026-06-05

**Authors:** Miguel Matos, Ivana Bilic, Nicolas Viloux, Barbara Jaskulska, Fatou Geißler, Yasamin Vali, Eberhard Ludewig, Olivier Albaric, Susanne Richter, Dieter Liebhart, Nicola Palmieri, Michael Hess

**Affiliations:** 1 Clinical Unit for Poultry Medicine, University of Veterinary Medicine, Vienna, Austria; 2 Labovet Conseil, Les Herbiers, France; 3 Diagnostic Imaging - Clinical Center for Small Animal Health and Research, University of Veterinary Medicine, Vienna, Austria; 4 Independent Pathologist, Lasseube, France; 5 Institute for Veterinary Disease Control, Division for Animal Health, Austrian Agency for Health and Food Safety, Mödling, Austria; 6 Present Address: Histovac GmbH, Klosterneuburg, Austria

**Keywords:** Viral pathogenesis, Virus-host interactions, Infection, Pathogens

## Abstract

Pegiviruses are generally regarded as non-pathogenic viruses with controversial clinical significance. Here, we describe an avian pegivirus (partridge pegivirus, ParPgV) associated with field outbreaks of encephalitis in red-legged partridges (*Alectoris rufa*). Next-generation sequencing identified ParPgV in brain tissues, revealing two distinct avian-origin pegiviruses. Histopathology and electron microscopy revealed encephalitic lesions, neuronal degeneration, and viral particles within neurons. Field surveillance demonstrated widespread vertical transmission across multiple partridge flocks. Experimental inoculation of red-legged partridges, grey partridges, and specific-pathogen-free chickens demonstrated viral neurotropism and systemic distribution with differences in humoral immune response. Infected red-legged partridges developed cerebellar atrophy detectable by MRI. Detection of negative-strand RNA replication intermediates confirmed active viral replication across different experimental hosts, and RNAscope in situ hybridization and immunohistochemistry further confirmed viral RNA and antigen in neural and lymphoid tissues. Here, we show experimental evidence supporting an association between a pegivirus and encephalitis, and suggest underappreciated neuropathogenic potential.

## Abstract (web summary)

In this study, the authors identify an avian pegivirus that is associated with brain inflammation in red-legged partridges and using experimental infection in different avian species they demonstrate viral neurotropism, revealing a potential role of pegiviruses in neurological disease.

## Introduction

Pegiviruses belong to the genus *Pegivirus* within the family *Flaviviridae* and are currently classified into 11 recognized species based on amino acid sequence divergence in conserved regions of NS3 and NS5B^1,2^. These viruses are enveloped, spherical particles measuring 50–100 nm in diameter, with a positive-sense single-stranded RNA genome ranging from 8.9 to 11.3 kb^1^. Pegiviruses have been identified in a broad range of mammalian hosts, including humans, non-human primates, horses, pigs, rodents, and bats^3,4^. More recently, the first non-mammalian pegiviruses were reported in diseased geese and metagenomic studies of healthy wild birds in China, Australia, and New Zealand, although their clinical relevance remains unclear^5–9^. In addition, the extent to which pegiviruses can transmit across different host species remains poorly understood^3^.

Historically referred to as GB viruses or hepatitis G viruses (HGV), pegiviruses establish persistent infections, as reflected in their name (pe, persistent; g, GB or G)^10^. Initially, members of *Pegivirus hominis* and *Pegivirus equi* were implicated in human and equine hepatitis (Theiler’s disease), respectively^10,11^. However, subsequent studies failed to substantiate these associations, and pegiviruses are largely considered non-pathogenic^4,12,13^. Nonetheless, human pegivirus (HPgV) infection has been linked to an increased risk of developing non-Hodgkin’s lymphoma and has also been associated with encephalitis^3^. In support of this, HPgV RNA and viral antigen have been detected in post-mortem brain tissue of patients with encephalitis, and in vitro studies have demonstrated viral replication in human astrocytes and microglia^14,15^. The primary cellular targets of pegiviruses remain uncertain, representing a major obstacle to advancing mechanistic studies on infection and pathogenesis, particularly due to the absence of a robust in vitro culture system. Despite this limitation, pegiviruses are well recognized as lymphotropic viruses, with previous studies highlighting the bone marrow and spleen as key target organs^8,16,17^. Additionally, HPgV has been shown to exert immunomodulatory effects that enhance survival in patients co-infected with HIV, hepatitis C virus (HCV), and Ebola virus^3^.

In this study, we investigated outbreaks of viral encephalitis in red-legged partridges (*Alectoris rufa*) from affected flocks in France. Initial diagnostic efforts targeting known avian neurotropic viruses—including avian encephalomyelitis virus, avian influenza virus, different arboviruses, and Marek’s disease virus—yielded negative results, prompting a non-targeted virological approach. This led to the identification of an avian pegivirus, designated partridge pegivirus (ParPgV), in the brain tissue of affected birds. Its consistent detection in encephalitis cases prompted the hypothesis that ParPgV was the causative agent of the outbreaks. To investigate this, we conducted comprehensive genomic characterization, field outbreak analysis together with epidemiological investigations, and performed in vivo studies in both homologous and heterologous hosts, with detailed pathological and imaging approaches providing key insights into its viral dynamics, infection kinetics, and host range. These findings strongly support the role of ParPgV as the etiological agent of the observed clinical signs.

## Results

### Outbreak description and clinical observations

Clinical signs suggestive of CNS involvement, including apathy, torticollis, ataxia, and prostration (Supplementary Movie 1), were generally transient, sporadic, and subtle, and were often overlooked by farm personnel. Affected birds were commonly culled upon detection, and this management practice may have influenced the observed pattern of sporadic, self-limiting cases. These clinical occurrences were not initially considered economically relevant. This perception changed in 2021, when a more severe and prolonged outbreak occurred during the laying period on one of the affected farms. At the time, the breeder flock consisted of 6550 pairs of red-legged partridges. The onset of the outbreak coincided with the beginning of the laying cycle in February/March and was characterized by persistent and recurring neurological signs. Farmers reported culling 10 to 20 birds per week during the first weeks of the season. However, by April, due to the substantial impact of culling on flock productivity (removal of actively laying breeders) and in the absence of evidence for vertical transmission, culling was halted, as affected birds continued to lay eggs despite exhibiting clinical signs. It is estimated, based on clinical observations, that at least 2% of birds were affected over the 16-week laying period, with egg production being reduced by approximately 4.6 eggs per hen in 2021 compared to other years. No bacterial pathogens were isolated during routine diagnostic investigations conducted at the time of the outbreak.

### Gross, histopathological, and ultrastructural evidence of viral encephalitis in affected partridges

**Fig. 1:**
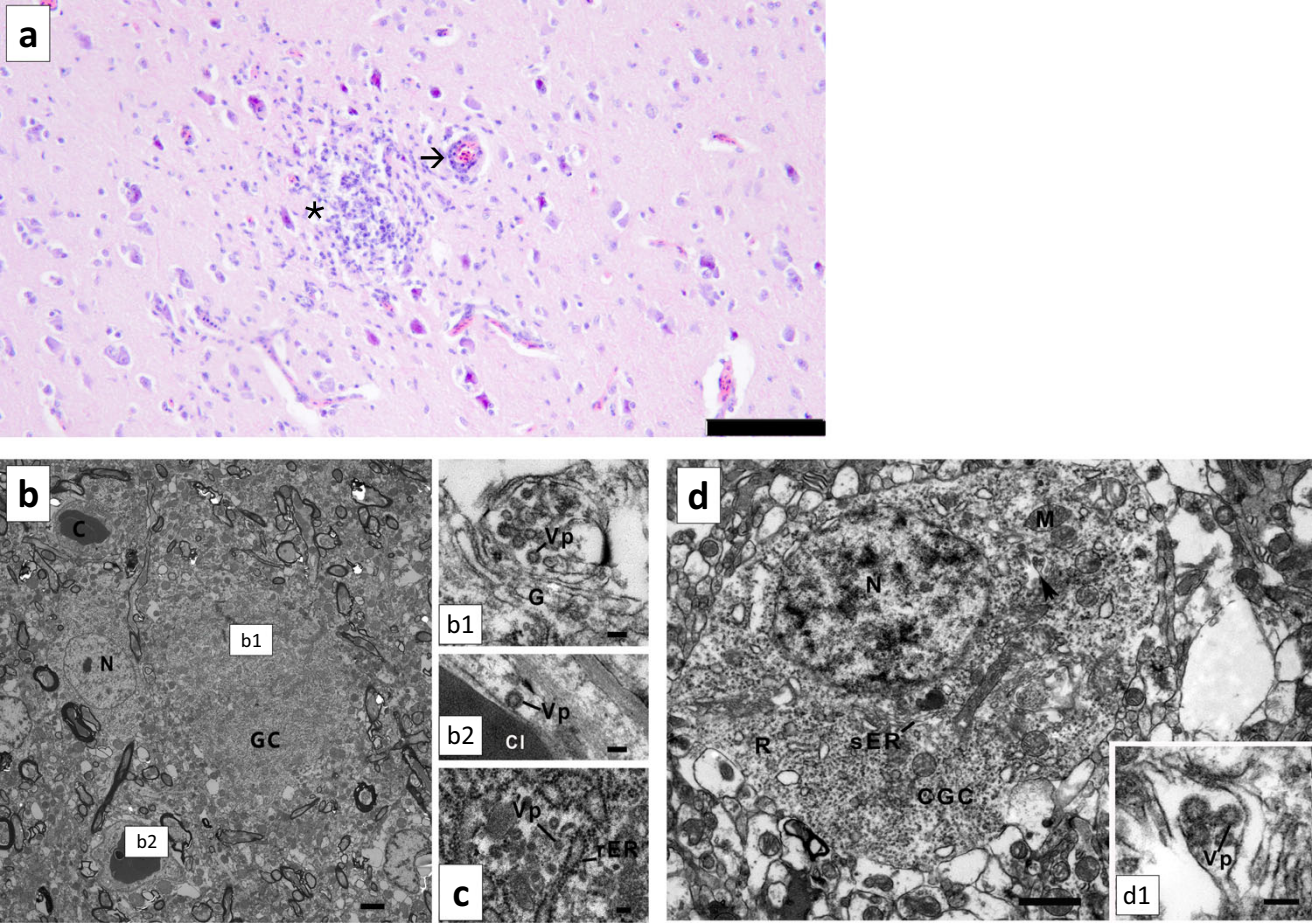
Histopathological and ultrastructural findings in the brain of red-legged partridges with encephalitis. **a** Histopathological changes in the brain. Hematoxylin and eosin (H&E) staining reveals marked perivascular cuffing composed of mononuclear inflammatory cells (lymphocytes and macrophages) (arrow). Diffuse microgliosis and scattered microglial nodules are evident within the neuropil (*), along with neuronal degeneration and reactive astrocytosis (scale bar = 100 µm). **b**–**d** Ultrastructural evidence of virus particles in the cerebrum and cerebellum. **b** Cerebrum–granular layer, granular cell (GC). Virus particles are observed in transport vesicles near the Golgi complex (inset b1) and in close association with the luminal wall of a capillary (inset b2). **c** Cerebrum–pyramidal layer. Clusters of virus particles are located in the cytoplasm of a pyramidal cell, associated with rough endoplasmic reticulum (Nissl bodies). **d** Cerebellum–granular layer, cerebellar granular cell (CGC). Virus particles are visible in transport vesicles adjacent to the smooth endoplasmic reticulum (inset d1). Annotations: C = capillary, Cl = capillary lumen, G = Golgi complex, N = nucleus, M = mitochondrion, R = ribosomes, rER = rough endoplasmic reticulum, sER = smooth endoplasmic reticulum, Vp = virus particles. Scale bars: **b** 2.5 µm (main image), 100 nm (insets); **c** 100 nm; **d** 1 µm (main image), 100 nm (inset). Similar histopathological and ultrastructural findings were observed in all examined partridge brain samples (*n* = 3), with no variation in lesion pattern.

At necropsy, affected partridges from the field outbreaks did not exhibit gross lesions consistent with the observed clinical signs. However, histopathological examination of the brain revealed widespread inflammatory lesions affecting all regions, including both white and, predominantly, grey matter (Fig. 1a). These lesions were characterized by foci of gliosis frequently associated with neuronal degeneration, necrosis, or, in some cases, vacuolation of the neuropil. Consistent perivascular mononuclear infiltrates, composed mainly of lymphocytes and macrophages, were observed.

Transmission electron microscopy (TEM) revealed the presence of viral particles, 70–90 nm in diameter, within neurons in both the cerebrum and cerebellum (Fig. 1b–d). In the cerebrum, these particles were detected in the perikaryon of granular and pyramidal cells (Fig. 1b, c), whereas, in the cerebellum, they were localized within cerebellar granular cells (Fig. 1d). The viral particles were found free in the cytoplasm or associated with transitional vesicles of the endoplasmic reticulum and Golgi complex. Additionally, viral particles were identified in the cytoplasm of endothelial cells lining the cerebral capillaries (Fig. 1b, inset b2). Morphologically, the particles displayed a spherical shape with a dense, rounded core surrounded by a diffuse outer layer.

### Discovery of a novel pegivirus by NGS

**Fig. 2:**
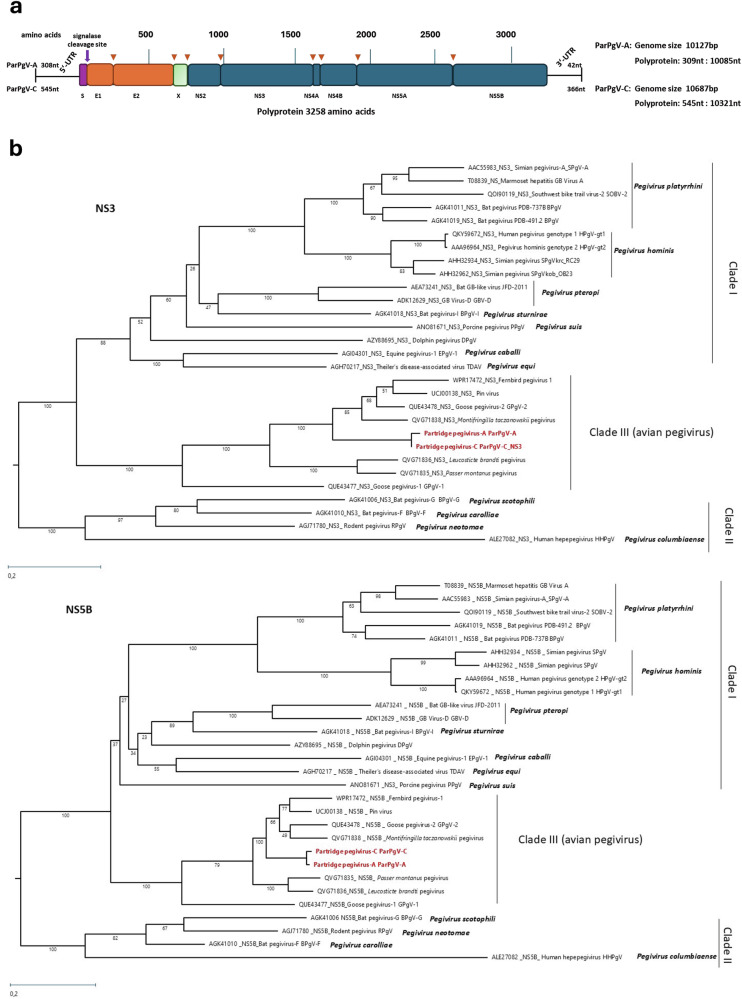
Genomic features of novel partridge pegivirus (ParPgV). **a** Schematic representation of the ParPgV genome and polyprotein organization. **b** Phylogenetic trees based on the NS3 and NS5B regions of the viral polyprotein, inferred using the Maximum Likelihood method (RAxML) from amino acid alignments of conserved sites (544 and 424 positions for NS3 and NS5B, respectively). The analysis included 27 pegivirus polyprotein sequences. Sequences from NCBI GenBank are labelled with accession numbers and names; ParPgV strains are highlighted in bold red. Pegivirus species as defined by ICTV are indicated. Branch support was assessed by bootstrap analysis with 100 replicates; bootstrap values are shown at the corresponding nodes. Trees are drawn to scale, with branch lengths proportional to the number of substitutions per site. Evolutionary analyses were performed using the MegAlign Pro module of Lasergene v18.0 (DNASTAR, Madison, WI, USA).

Brain samples from field outbreaks were screened by PCR for avian influenza virus, avian encephalomyelitis virus, Marek’s disease virus, and arboviruses of the genus *Orthoflavivirus*. All assays returned negative results, thereby excluding these neurotropic avian pathogens as the cause of the observed clinical presentation. To further investigate the aetiology of the outbreaks, RNA extracted from brain samples of all three outbreaks was subjected to next-generation sequencing (NGS). Metagenomic profiling using de novo assembled contigs identified 887 viral contigs, including three short contigs mapping to Goose pegivirus 2 (GPgV-2) and other avian-origin pegiviruses. The presence of an avian pegivirus was confirmed by conventional PCR targeting the 5’-UTR and NS4A regions, followed by Sanger sequencing of the PCR products, which indicated the presence of different strains among the samples. To obtain the complete genome sequence of one strain, designated ParPgV-A, a set of primers covering the pegivirus genome was designed (Supplementary Table 1, Supplementary Fig. 3a), resulting in a 10,127-bp final genome sequence. For the ParPgV-C strain, total RNA from infected partridge embryos was subjected to NGS, followed by PCR and Sanger sequencing to close gaps (Supplementary Table 1, Supplementary Fig. 3b). This yielded a final genome sequence of 10,687 bp, slightly larger than that of ParPgV-A. Both ParPgV genomes consist of short 5’- and 3’-untranslated regions (UTRs) and a single open reading frame (ORF). The longer UTRs observed in ParPgV-C suggest that the ParPgV-A genome may be incomplete. Similar to other pegiviruses, both ParPgV strains encode a single polyprotein of 3258 amino acids. ParPgV-A and ParPgV-C share 83.78% nucleotide identity over the complete genome and 95.64% amino acid identity over the polyprotein sequence. Sequence comparison with known pegiviruses suggested that the polyprotein encodes a signal peptide (S), two structural proteins (E1 and E2) at the N-terminal region, followed by six non-structural proteins (NS2, NS3, NS4A, NS4B, NS5A, and NS5B) (Fig. 2a). A putative signalase cleavage site was identified between amino acids 19 and 20. HMMER searches against the Pfam database identified protein domains conserved in other *Flaviviridae*, corroborating the predicted organization of the viral polyprotein (Supplementary Table 2). Phylogenetic analyses based on the NS3 and NS5B regions positioned both ParPgV strains on a distinct branch within the avian pegivirus clade, closely related to several strains of avian origin (Fig. 2b). In contrast to mammalian pegiviruses, which segregate into two clades, all avian-origin pegiviruses formed a third distinct clade. The percent identity matrix showed 97.98% amino acid identity between the two ParPgV strains and 59.38–77.39% identity to other avian-origin pegiviruses (Supplementary Table 3). Based on the proposed pegivirus species demarcation criterion of 69% amino acid identity within the NS3 region^2^, ParPgV belongs to the same species as Goose pegivirus 2 (GPgV-2), Fernbird pegivirus 1, *Monifringilla taczanowskii* pegivirus, and Pin virus. Furthermore, based on this demarcation criterion, the analysis indicated the existence of two additional species within the avian pegivirus clade. One represented by the Goose pegivirus 1 (GPgV-1) and the other one consisting of *Leucosticte brandti* pegivirus and *Passer montanus* pegivirus (Fig. 2b).

### Prevalence of ParPgV in red-legged partridge farms

**Fig. 3:**
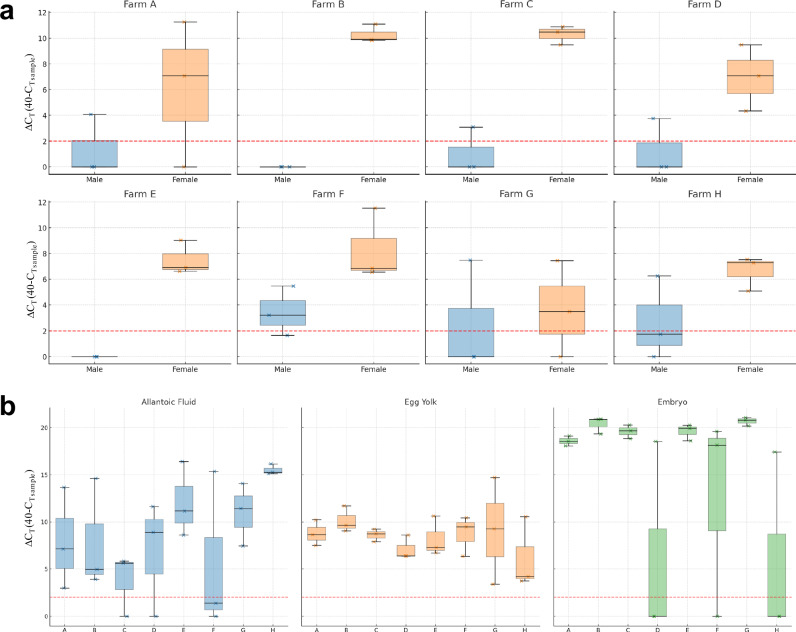
Field screening for pegivirus across eight farms (A–H). Viral load is presented as ΔC_T_ values (40 - C_T_ of the sample), with higher ΔC_T_ values indicating higher viral loads. Boxplots show the median (centre line), interquartile range (box; 25th–75th percentiles), and whiskers extending to the most extreme values within 1.5× the interquartile range. The plots display the distribution of ΔCt values, with individual jittered points representing each pool (*n* = 3). The red dashed line denotes the limit of detection (LOD; ΔCt = 2). Each farm was screened using 15 males and 15 females, as well as 30 embryonated eggs for allantoic fluid, egg yolk, and embryo. **a** Comparison of viral load between male and female birds across farms. Across multiple farms, female pools consistently exhibited higher viral loads than male pools, while male pools frequently clustered at or near the LOD, indicating low or absent detectable shedding. **b** Comparison of viral load in embryonated eggs across different sample types (AF, YS, and embryo). Allantoic fluid and yolk samples exhibited variable but generally moderate viral loads, whereas embryos frequently showed the highest ΔC_T_ values. Source data are provided as a Supplementary Information.

ParPgV was detected in cloacal swabs from all investigated farms (Fig. 3a). All pooled samples from female birds, except one from Farm G, tested positive. In males, all samples from Farms A and F were positive, while negative pools were observed in Farms B and E, and two of three pools were negative in Farms C, D, G, and H. Female samples showed higher positivity rates than male samples.

In embryonated eggs, all pooled yolk sac (YS) samples tested positive for ParPgV (Fig. 3b). Allantoic fluid (AF) samples from Farms A, B, E, G, and H tested positive, while negative pools were detected in Farms C, D, and F. Embryo samples were positive across all farms, with some negative pools identified in Farms D, F, and H. Viral loads were highest in embryos, followed by YS and AF.

### Pathogenicity and tissue distribution of ParPgV in experimentally inoculated birds

#### Grey partridges

**Fig. 4:**
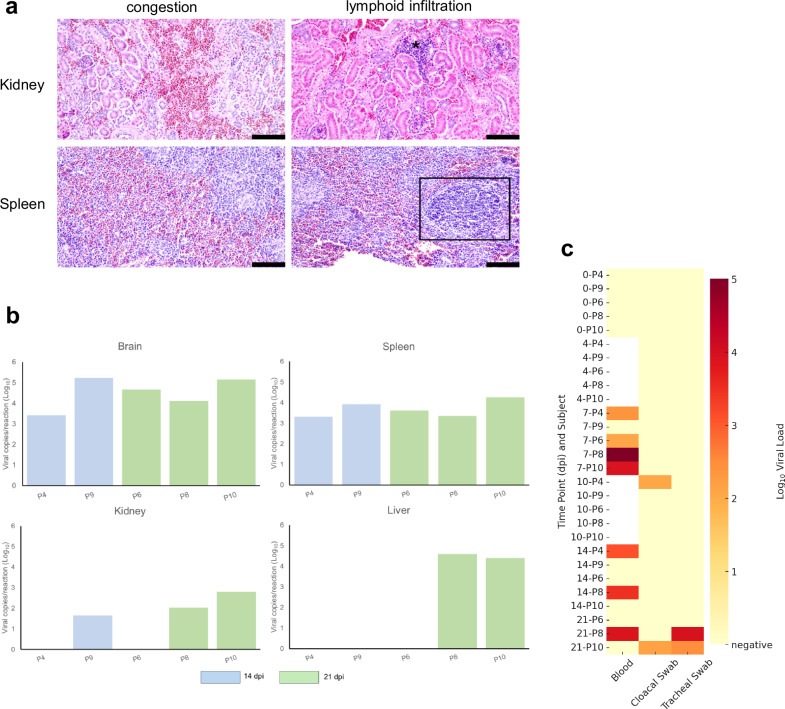
Histopathological changes and viral load dynamics in grey partridges (*Perdix perdix*) experimentally inoculated with mixed ParPgV inoculum. **a** Histopathological analysis of kidney and spleen tissues. Kidney section at 14 days post-inoculation (dpi) showing marked congestion and a focal area of lymphocytic infiltration (*). Spleen section at 21 dpi exhibiting congestion and areas of lymphocytic infiltration (black box). Hematoxylin and eosin (H&E) staining. Scale bar = 100 µm. Representative images are shown; comparable lesions were observed in 3/5 spleens and 5/5 kidneys across examined birds. **b** Viral load in brain, spleen, liver, and kidney from individual partridges (P4 and P9 at 14 dpi; P6, P8, and P10 at 21 dpi), expressed as viral copies per reaction (log_10_ scale). **c** Heatmap representation of viral load in blood, cloacal swabs, and tracheal swabs across different time points (0, 4, 7, 10, 14, and 21 dpi). Blood was collected weekly; swabs were collected at each indicated time point. Source data are provided as a Supplementary Information.

Of the seven inoculated grey partridges (P4–P10), two (P5 and P7) died shortly after inoculation due to unrelated causes. Clinical signs in the remaining birds were limited to transient reduction of flying behaviour observed at 4 dpi (Supplementary Movie 2). Histological examination revealed mild kidney, liver, and spleen alterations (Fig. 4a). Kidney congestion was observed in all inoculated birds, with some cases exhibiting lymphoid infiltration. Liver congestion was noted in most birds, except one. Splenic congestion ranged from mild to severe, with occasional lymphocytic infiltration and encapsulation of connective tissue. No significant histological changes were detected in other organs.

Post-mortem analysis detected viral RNA in the brain and spleen of all birds, with viral load values remaining stable between 14 and 21 dpi (Fig. 4b), ranging from 3.43 to 5.24 log_10_ viral copies/reaction in the brain and 3.35 to 4.28 log_10_ in the spleen. In the kidney, viral RNA was detected in 1 of 2 birds at 14 dpi and in 2 of 3 birds at 21 dpi, with viral loads reaching 2.80 log_10_ viral copies/reaction at the latter time point. Similarly, no viral RNA was detected in the liver at 14 dpi, whereas 2 of 3 birds were positive at 21 dpi, with viral loads reaching 4.60 log_10_ viral copies/reaction (Fig. 4b).

Real-time RT-qPCR analysis of swabs and whole blood revealed sporadic viral detection (Fig. 4c). Tracheal swabs tested positive only at 21 dpi in P8 and P10, while cloacal swabs showed positivity at 10 dpi in P4 and at 21 dpi in P10. In blood, all birds except P9 were positive at 7 dpi, whereas at 14 dpi only P4 and P8 remained positive. By 21 dpi, ParPgV RNA was detected solely in P8.

#### Red-legged partridges

**Fig. 5:**
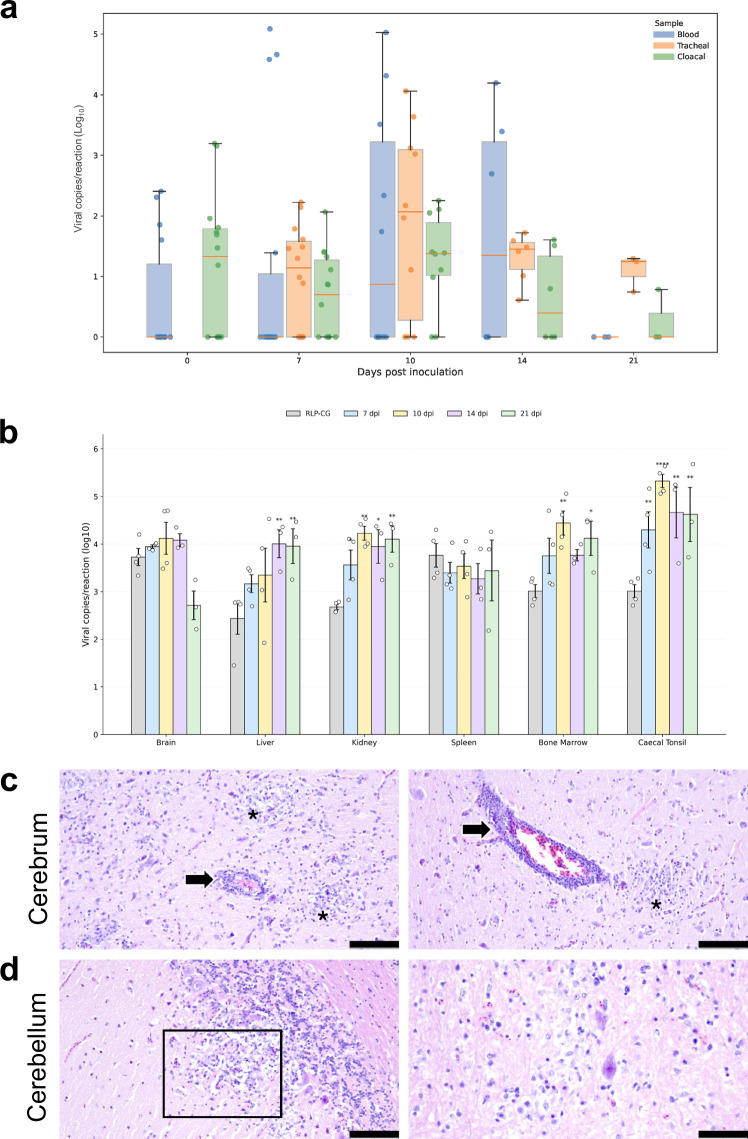
Viral load dynamics, histopathological lesions, and MRI findings in red-legged partridges (*Alectoris rufa*) experimentally inoculated with ParPgV-C strain. **a** Viral RNA levels were quantified in blood, tracheal swabs, and cloacal swabs collected from individual partridges at 0, 7, 10, 14, and 21 days post-inoculation (dpi), expressed as viral copies per reaction (log_10_). Tracheal swabs were not collected at 0 dpi. Boxplots show the median (centre line), interquartile range (25th–75th percentiles), and whiskers extending to the most extreme values within 1.5× the interquartile range, while overlaid jittered points represent individual animals. Sample size: *n* = 14 at 0 and 7 dpi; *n* = 10 at 10 dpi; *n* = 6 at 14 dpi; *n* = 3 at 21 dpi. Blood samples showed marked temporal variation in viral load, whereas tracheal and cloacal swabs exhibited peak viral loads at 10 dpi followed by a decline at later time points. **b** Viral load in brain, liver, kidney, spleen, bone marrow, and caecal tonsils at 7, 10, 14, and 21 dpi. Data are presented as mean ± standard error of the mean (SEM), with individual data points overlaid. Sample size: RLP-CG (*n* = 4), 7 dpi (*n* = 4), 10 dpi (*n* = 4), 14 dpi (*n* = 3), 21 dpi (*n* = 3) per organ. Statistical analysis was performed using a two-way ANOVA (two-sided) with factors organ and time point (dpi), followed by post hoc pairwise comparisons between each infected group and the control group (RLP-CG) within each organ. *P* values were adjusted for multiple comparisons using the Holm method. Exact adjusted *P* values for pairwise comparisons are provided in the Source data file. Statistically significant differences (*P* < 0.05) between ParPgV-inoculated partridges and control birds are indicated above the bars. Source data are provided as a Source data file. **c** Cerebrum at 21 dpi showing moderate to prominent perivascular cuffing (black arrow) with lymphocytes and histiocytes, multifocal gliosis, and neuronal degeneration (*). H&E staining; scale bars: 100 µm. **d** Cerebellum at 21 dpi displaying clear inflammatory infiltrates affecting molecular and granular layers, with neuronal degeneration and necrosis (box), and degenerated Purkinje neurons with vacuolated cytoplasm, nuclear condensation, and surrounding reactive glial cells (right). H&E staining; scale bars: 100 and 50 µm, respectively. Similar brain lesions were observed in all birds at 21 dpi (*n* = 3).

**Fig. 6:**
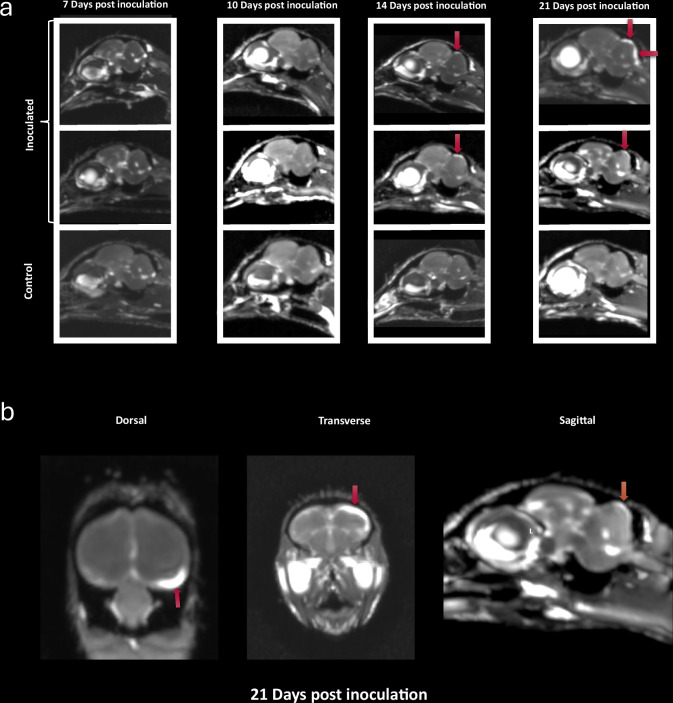
Magnetic resonance imaging findings in red-legged partridges experimentally inoculated with ParPgV-C strain. **a** Representative T2-weighted CISS sagittal MRI images of ParPgV-inoculated red-legged partridges at 7, 10, 14, and 21 days post-inoculation (dpi) compared with a control bird. In inoculated birds, progressive widening of the subarachnoid space at the cerebellar level (red arrows) becomes apparent at later time points, most prominently at 21 dpi, whereas control birds show no comparable changes. **b** Multiplanar T2-weighted MRI views (dorsal, transverse, and sagittal) of a ParPgV-inoculated red-legged partridge at 21 dpi illustrating asymmetric widening of the subarachnoid space in the caudal cranial fossa (arrows), consistent with cerebellar volume loss. MRI findings were interpreted qualitatively and are shown as representative images.

Prior to inoculation, whole blood and cloacal swabs from all red-legged partridges revealed low levels of ParPgV RNA (Fig. 5a, 0 dpi), confirming the field surveillance findings described above. Following inoculation with the ParPgV inoculum, birds exhibited decreased activity and reduced flying behaviour between 5 and 8 dpi, with no other signs being observed. The viral RNA load in blood, tracheal, and cloacal swabs increased until 10 dpi, subsequently declining, with lower levels detected at 21 dpi. At this final time point, no viral RNA was detected in blood samples. Viral RNA was consistently detected in all examined organs throughout the experiment, including the brain, liver, kidney, spleen, bone marrow, and caecal tonsils (Fig. 5b). Following ParPgV inoculation, viral load significantly increased in the liver, kidney, bone marrow, and caecal tonsils compared to PBS-inoculated controls (RLP-CG). In the brain, viral load remained stable until the final time point, when a non-significant decline was observed. In contrast, viral load in the spleen remained unaffected by inoculation. Histological examination revealed no observable lesions until 21 dpi. At this final time point, all ParPgV-inoculated birds displayed encephalitic lesions, both in the cerebrum and cerebellum, characterized by gliosis, neuronal degeneration or necrosis, and perivascular mononuclear cell infiltrations (Fig. 5c, d). Magnetic resonance imaging (MRI) demonstrated progressive widening of the subarachnoid space in the caudal cranial fossa on fluid-sensitive sequences (T2W-CISS and T2-TIR) in ParPgV-inoculated red-legged partridges, becoming most evident at 21 dpi when compared with both control birds and earlier time points (Fig. 6a, b). This finding was observed consistently across inoculated individuals and was not present in controls. Based on qualitative assessment, the changes are compatible with cerebellar volume loss. No additional parenchymal signal abnormalities or focal alterations in signal intensity were detected in other brain regions, suggesting a localized rather than diffuse CNS involvement.

#### SPF chickens

**Fig. 7:**
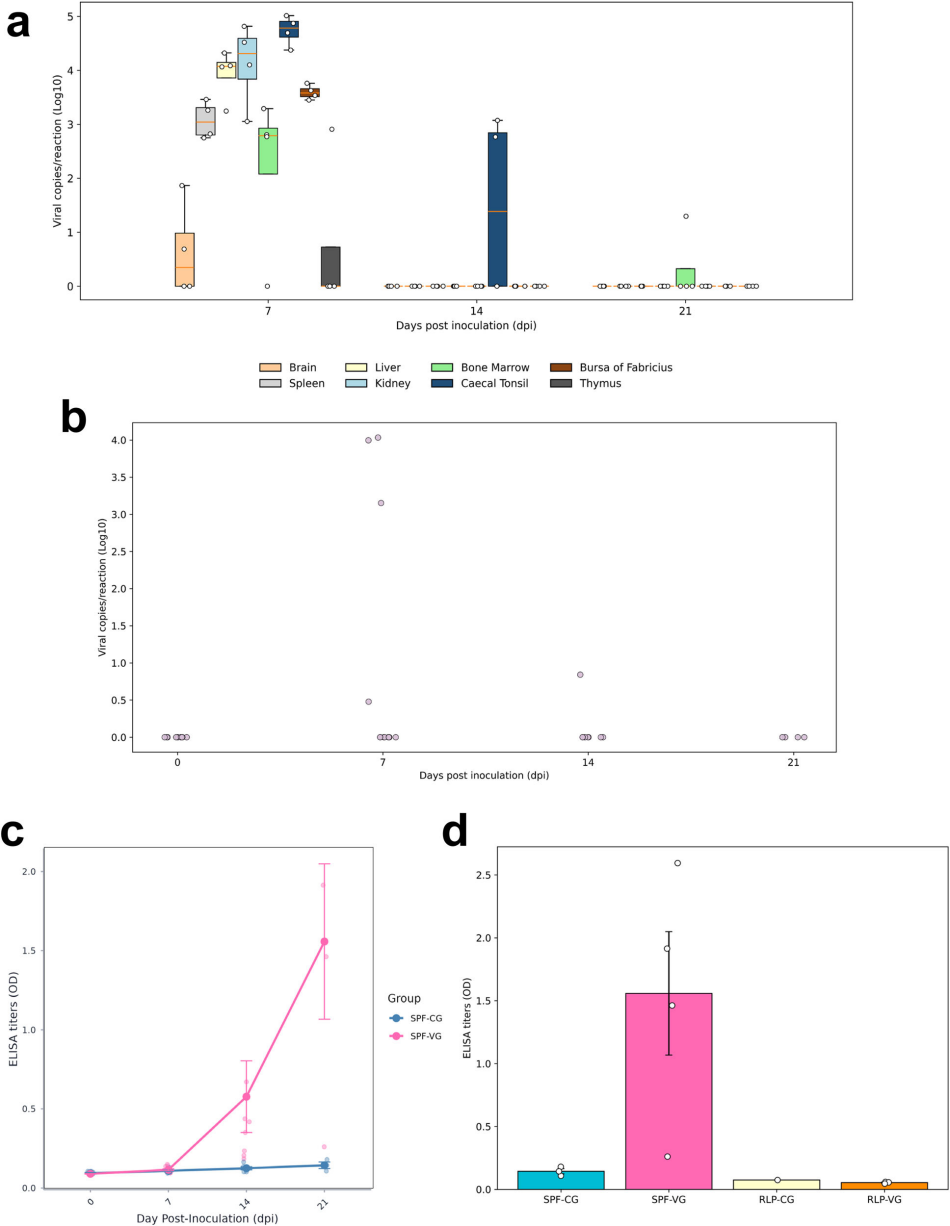
Viral kinetics in SPF chickens and comparative humoral immune responses in SPF chickens and red-legged partridges following experimental infection with ParPgV-C. Viral load is expressed as viral copies per reaction (Log_10_). **a** Box plot showing viral load detected in brain, spleen, liver, kidney, bone marrow, caecal tonsil, bursa of Fabricius, and thymus in SPF chickens at 7, 14, and 21 days post-inoculation (dpi), with highest viral loads detected at 7 dpi, and persistence in caecal tonsil and bone marrow at later time points. Boxplots show the median (centre line), interquartile range (25th–75th percentiles), and whiskers extending to the most extreme values within 1.5× the interquartile range. Individual data points are shown as jittered dots (*n* = 4 per organ per time point). **b** Strip plot of individual viral load in blood samples from SPF chickens at 0, 7, 14, and 21 dpi, showing peak viremia at 7 dpi, followed by a decline. **c** Longitudinal ELISA optical density (OD) values in SPF chickens from control (SPF-CG) and virus-inoculated (SPF-VG) groups at 0, 7, 14, and 21 dpi (mean ± SE). Individual data points are shown as jittered dots. Sample size at consecutive time points, respectively: SPF-VG (*n* = 12, 12, 8, 4) and SPF-CG (*n* = 8, 8, 6, 3). **d** ELISA OD values at 21 dpi comparing control and infected groups of SPF chickens (SPF-CG, SPF-VG) and red-legged partridges (RLP-CG, RLP-VG). Bars represent mean ± SE. SPF-VG chickens show a marked increase in ELISA reactivity at the end of the experiment, whereas no comparable serological response is detected in red-legged partridges. Individual data points are shown as jittered dots (SPF-CG: *n* = 3; SPF-VG: *n* = 4; RLP-CG: *n* = 1; RLP-VG: *n* = 3). Source data are provided as a Source data file.

SPF chickens were included as a heterologous host to assess susceptibility and to explore their potential use as a defined laboratory model. Birds were inoculated at 4 weeks of age, and no clinical signs were observed throughout the study. However, viral RNA was detected in all investigated organs at 7 days post-inoculation (dpi), with the highest loads found in the liver, kidney, caecal tonsils, and bursa of Fabricius (Fig. 7a). By 14 dpi, viral RNA was detected only in the caecal tonsils, and by 21 dpi, it was restricted to the bone marrow, with all other organs testing negative.

Prior to inoculation, blood and cloacal swabs from SPF chickens tested negative for ParPgV by PCR (Fig. 7b). Following inoculation, viremia peaked at 7 dpi, with four birds testing positive for viral RNA in blood. At 14 dpi, only one bird remained positive, with a low viral load, and by 21 dpi, all birds were negative. All cloacal swabs remained negative throughout the study (Fig. 7b).

No histological lesions were observed in any of the organs examined at any time point, and MRI analysis revealed no detectable abnormalities.

### Humoral response to ParPgV infection in chickens and red-legged partridges

To assess the humoral immune response following experimental ParPgV infection, serum samples were analysed by ELISA coated with the ParPgV E2 protein. In SPF chickens, virus-inoculated birds (SPF-VG) exhibited a clear time-dependent increase in ELISA reactivity, becoming evident by 14 dpi and further increasing by 21 dpi, while control birds (SPF-CG) remained at baseline levels throughout the experiment (Fig. 7c). This progressive rise in optical density values indicates the development of a virus-specific antibody response in infected chickens.

Comparison of ELISA results at the end of the experiment (21 dpi) across species revealed a pronounced serological response in SPF-VG chickens, in contrast to both SPF-CG controls and red-legged partridges (Fig. 7d). In the red-legged partridge experiment, serological assessment was prioritised at the terminal time point to evaluate antibody development at the end of the infection course. At this time point, neither control nor virus-inoculated red-legged partridges (RLP-CG and RLP-VG) showed a measurable increase in ELISA signal, with OD values remaining close to background levels. These findings indicate a marked species-dependent difference in humoral immune responses to ParPgV infection, with robust antibody induction in chickens but an absence of detectable seroconversion in red-legged partridges under the applied conditions.

### Immunohistochemistry (IHC) and RNAscope in situ hybridization investigations

**Fig. 8:**
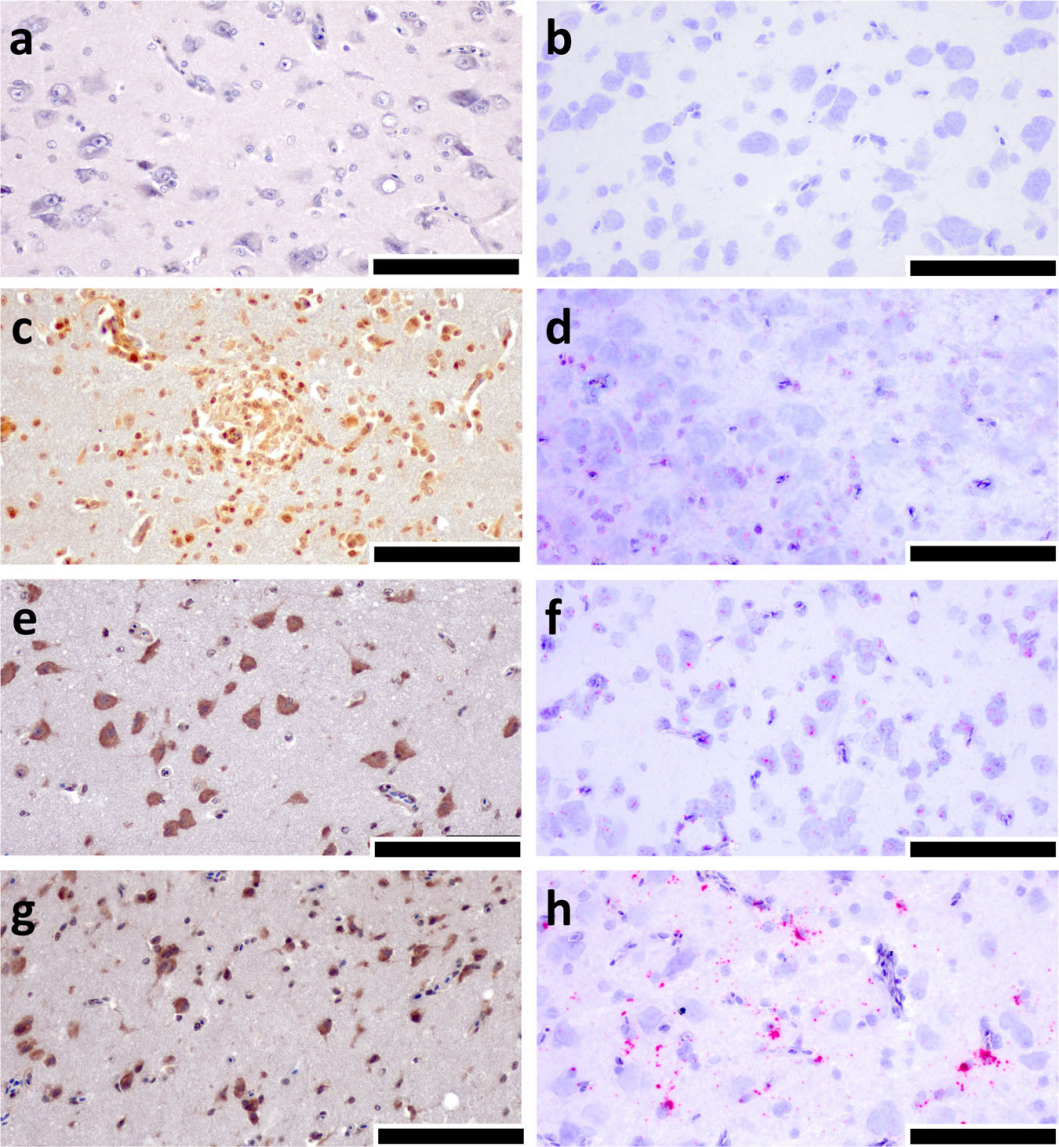
Detection of ParPgV antigen and RNA in brain tissue from field and experimentally infected partridges using immunohistochemistry (IHC) and RNAscope in situ hybridization. Brain sections from negative control birds show no ParPgV staining by IHC (**a**) and no RNA hybridization signal by RNAscope (**b**). Strong immunoreactivity was observed in the cerebrum of naturally infected red-legged partridges (**c**), experimentally inoculated grey partridges (**e**), and red-legged partridges (**g**). Corresponding RNAscope signals localized ParPgV RNA to neuronal cell bodies and glial cells in the cerebrum of naturally infected red-legged partridges (**d**), experimentally inoculated grey (**f**), and red-legged partridges (**h**). Counterstaining with hematoxylin was omitted in (**c**). Scale bars: 100 µm. Analyses were performed on tissues previously characterized by histopathology and/or qPCR, and consistent signal distribution patterns were observed across all examined samples (*n* = 3).

**Fig. 9:**
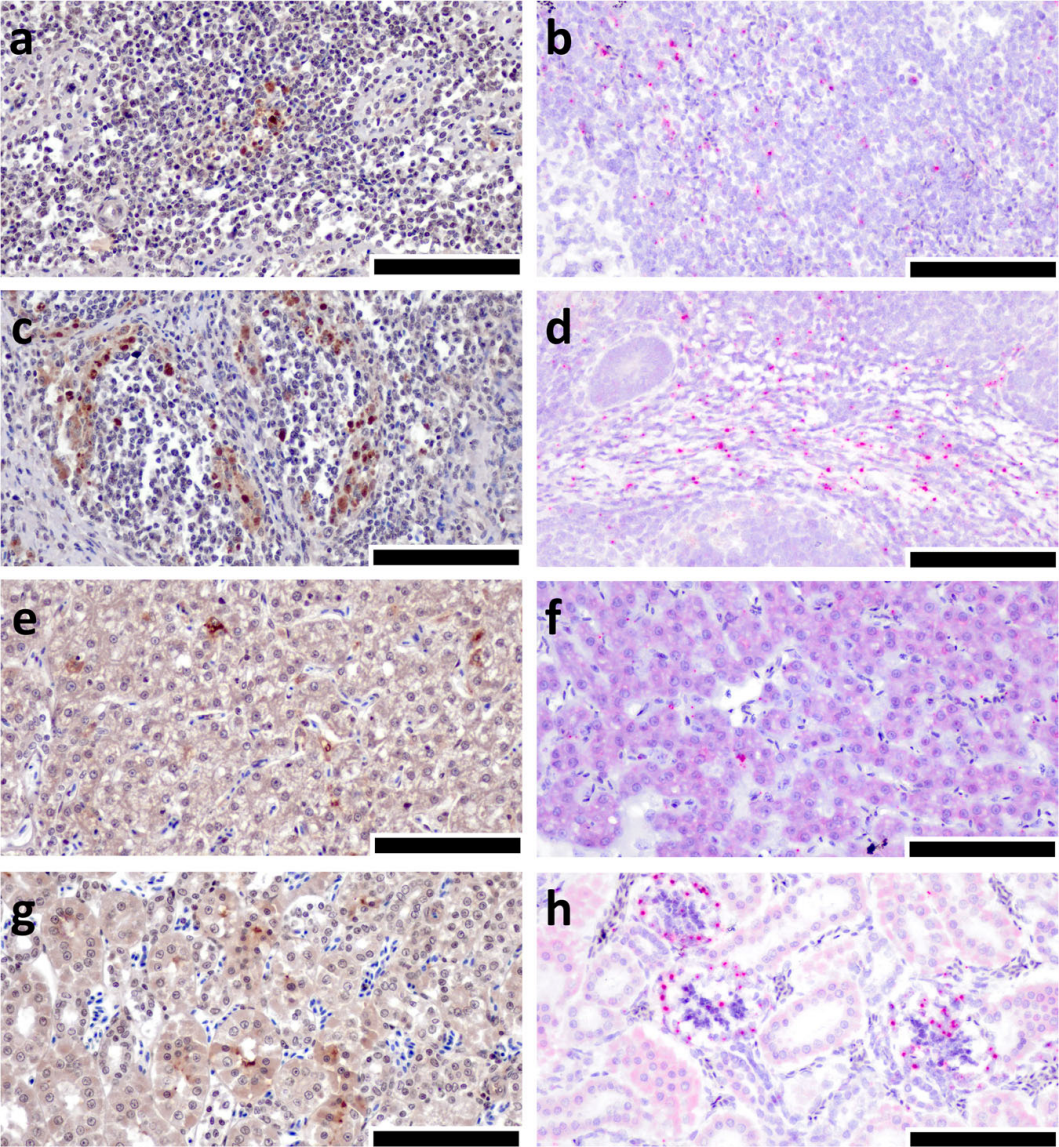
Detection of ParPgV antigen and RNA in systemic organs of experimentally inoculated red-legged partridges. ParPgV antigen was detected by IHC in the spleen (**a**), particularly within periarteriolar lymphoid sheaths, and in caecal tonsils (**c**), liver (**e**), and kidney (**g**). RNAscope hybridization confirmed the presence of viral RNA in the same tissues: lymphoid follicles and reticuloendothelial cells in the spleen (**b**), follicular epithelium and mononuclear cells in the caecal tonsils (**d**), hepatocytes and sinusoidal lining cells in the liver (**f**), and tubular epithelial cells and glomerular structures in the kidney (**h**). Scale bars: 100 µm. Analyses were performed on tissues previously characterized by histopathology and qPCR, and consistent signal distribution was observed across all examined samples (*n* = 3).

To assess the neurotropism of ParPgV in infected birds, IHC and RNAscope in situ hybridization were performed on brain samples from both field outbreaks and the three in vivo experiments (Fig. 8). To investigate systemic distribution, spleen, caecal tonsils, liver, and kidney tissues from experimentally inoculated red-legged partridges were similarly analysed (Fig. 9). A primary antibody dilution of 1:500 yielded optimal signal intensity in IHC and was standardized across all samples.

In the brain, contrary to investigations in negative birds (Fig. 8a, b), IHC confirmed the presence of ParPgV antigen, particularly in perivascular regions and areas of inflammatory infiltrates in naturally infected partridges (Fig. 8c). RNAscope detected viral RNA within neuronal and glial cells of the cerebrum (Fig. 8d). Experimentally inoculated grey and red-legged partridges showed diffuse immunoreactivity throughout the neuropil (Fig. 8e, g). RNAscope also revealed strong signals in Purkinje cells and scattered glial cells (Fig. 8f, h), extending into the cerebellum of red-legged partridges, possibly suggesting viral involvement in motor coordination centers. These findings correlate with the observed neurological signs in infected birds. In SPF chickens, brain sections from PCR-positive individuals showed only faint signals, consistent with lower viral loads.

In the spleen, viral antigen was localized to periarteriolar lymphoid sheaths (PALS) and reticuloendothelial cells (Fig. 9a), with RNAscope confirming viral RNA in the same regions (Fig. 9b). In caecal tonsils, both antigen and RNA were detected in the follicular epithelium and scattered mononuclear cells (Fig. 9c, d). In the liver, viral antigen and RNA were found in hepatocytes and sinusoidal lining cells (Fig. 9e, f). In the kidney, both signals were observed in tubular epithelial cells and glomerular structures (Fig. 9g, h).

### ParPgV negative-strand RNA replication

Detection of the negative-strand RNA, an intermediate of viral replication, was assessed by strand-specific RT-PCR across different tissues and species (Supplementary Table 4). In the brain, negative-strand RNA was consistently detected in inoculated red-legged partridges (RLP-VG group) and grey partridges (GP). Interestingly, control red-legged partridges (RLP-CG group), which were vertically infected in ovo, also tested positive. Additionally, in red-legged partridge embryonated eggs, negative-strand RNA was detected in both the embryos and the yolk sac. In SPF chickens, investigation of brain tissue did not yield conclusive results due to the presence of low viral load (see Fig. 7a). Nonetheless, positive results were obtained from the liver, kidney, spleen, bone marrow, and caecal tonsils in SPF chickens.

## Discussion

Pegiviruses were initially suspected to cause hepatitis due to their association with non-A–E hepatitis in humans and genomic similarities to hepatitis C virus (HCV)^4^. However, extensive studies have since shown that pegiviruses are not directly linked to acute or chronic hepatitis^3^. Most available data come from human pegivirus (HPgV-1), which is widely considered non-pathogenic, though its immunomodulatory effects have gained interest for their potential benefits in co-infections^3^. Recently, however, HPgV-1 has been implicated in fatal leukocytic encephalitis, with evidence of lymphocytic infiltration and gliosis in brain tissue^14^. Further supporting its neurotropism, studies have demonstrated HPgV-1 replication in astrocytes and microglia, highlighting its ability to infect neural cells^15^. In agreement with this, the actual study provides experimental evidence that pegiviruses can cause encephalitis, challenging the prevailing assumption that members of the *Pegivirus* genus are non-pathogenic.

A key finding of this work was the detection of encephalitis in red-legged partridges during field outbreaks, which included histopathological lesions consistent with viral infection and electron microscopy confirmation of virus-like particles in affected neurons. Despite repeated attempts to propagate the virus in primary liver chicken embryo cells and via yolk sac inoculation of chicken embryos, viral titers declined over subsequent passages, indicating unsuccessful in vitro propagation under the conditions tested. This aligns with the broader challenge of establishing efficient in vitro systems for pegiviruses, which often demonstrate narrow tropism and can be notoriously difficult to culture^4^. In agreement with this, a goose pegivirus (GPgV) was passaged eight times in primary goose embryonic fibroblasts, reaching high viral loads, but failed to replicate in other five different cell types^8^. Notwithstanding these limitations, next-generation sequencing of clinical material revealed pegiviral sequences, confirming the presence of an avian pegivirus as a likely etiological agent.

Detailed sequence analysis revealed the presence of potentially more than one pegivirus strain in the outbreak samples. This was confirmed by obtaining the complete genome sequences of two distinct strains, designated as ParPgV-A and ParPgV-C. Among these, the ParPgV-C strain appears to be more prevalent in red-legged partridges, as it was detected in both embryos and outbreak samples. Phylogenetic analysis supports the previously proposed existence of a third clade within the genus *Pegivirus*, comprising all reported avian-origin pegiviruses^6,8^. Moreover, based on the species demarcation threshold of 69% amino acid identity within the NS3 region, our findings confirm the presence of three distinct species within this third Pegivirus clade^2^. The ParPgV strains identified in this study cluster within the species that includes the majority of currently reported avian pegiviruses. However, no distinct phylogenetic clustering was observed correlating with clinical presentation. This may be because most avian pegiviruses reported to date have been identified through metagenomic surveys of asymptomatic birds, whereas the ParPgV genomes described here are among the few sequences present to date associated with clinical disease.

An initial experiment in grey partridges was conducted as an exploratory model, partly because red-legged partridges are native to southern Europe and were less accessible in Austria. Despite this limitation, the study confirmed not only the neurotropic and lymphotropic nature of ParPgV but also its propensity for persistence. Stable viral loads were detected in the brain and spleen until the end of the trial, whereas viral RNA in blood and swabs fluctuated or was only intermittently positive. However, no histopathological lesions were evident in the brain of grey partridges. By contrast, in the subsequent experiment involving red-legged partridges—the original host of ParPgV—encephalitic lesions were observed upon histopathological examination, and cerebellar atrophy was confirmed by MRI, although clinical signs were limited to subtle behavioural changes such as reduced activity and flight responsiveness. The cerebellar atrophy noted in superinfected birds suggests a progressive, degenerative process likely associated with chronic infection and might explain the ataxia and prostration observed in affected birds in the field. Such findings parallel recent reports implicating HPgV-1 in neurological disease, in which a leukoencephalitis patient presented progressive lesions in the white matter by MRI investigation, with involvement of the brainstem and cervical cord^14^. Additionally, the present findings resemble outcomes in other experimental neurotropic flavivirus models, where infection-induced neuronal loss and persistent inflammation lead to long-term neuropathological changes^18,19^. Nonetheless, these results differ from those in a rhesus monkey pegivirus model, which did not demonstrate increased viral RNA loads in neural tissues^16^.

The combined RNAscope ISH and IHC findings reinforce the hypothesis that ParPgV exhibits a strong neurotropic and systemic infection pattern. The presence of viral RNA and antigen in the cerebrum and cerebellum supports the notion that ParPgV actively replicates in neural tissues, contributing to encephalitic lesions and cerebellar atrophy observed in red-legged partridges. Additionally, viral localization in immune-associated tissues such as the spleen and caecal tonsils suggests that the virus persists in lymphoid organs, potentially modulating immune responses. These findings were further corroborated by strand-specific RT-PCR detection of the negative-strand RNA replication intermediate, confirming active viral replication in the brain tissues of inoculated red-legged partridges and grey partridges. Moreover, negative-strand RNA was identified in the liver, kidney, spleen, bone marrow, and caecal tonsils of SPF chickens, and in the embryos and yolk sacs of red-legged partridge embryonated eggs, providing evidence of systemic and in ovo viral replication. The detection of ParPgV RNA and antigen in renal and hepatic tissues further highlights the systemic nature of the infection, raising questions about the potential for viral shedding and long-term persistence. These findings are consistent with other neurotropic flavivirus infections, where persistent viral replication in neural and immune tissues leads to chronic pathology and progressive neurological impairment^20–23^. Further research is needed to clarify whether viral persistence in these organs contributes to long-term disease progression and whether immune modulation plays a role in maintaining viral reservoirs in infected hosts.

Interestingly, although red-legged partridges developed clear neurological lesions in this study, they displayed only mild outward signs of disease, implying that ParPgV could cause a subclinical or progressive form of disease, as it has been reported in mice experimentally inoculated with West Nile virus^18^. This possibility may have implications for both wild and farmed partridge populations, as clinically apparent signs might be infrequent or overlooked, especially in flocks where routine culling removes symptomatic individuals.

A critical limitation of the red-legged partridge experiment lies in the fact that these birds already tested positive for ParPgV at the time of inoculation, likely through vertical transmission. Screening of multiple partridge flocks failed to identify birds free of ParPgV; moreover, females consistently harboured higher viral loads than males. This sex-associated difference in viral load, along with the temporal association of clinical outbreaks with the onset of egg laying, raises the hypothesis that physiological or hormonal changes linked to reproduction, such as immunomodulation during the laying period, may favour viral replication or persistence in females. In agreement with this, embryonated eggs from all flocks were also positive, strongly indicating that vertical transmission occurs regularly in these populations. Vertical mother-to-child transmission of HPgV has been reported in humans^24–27^, however, avian pegiviruses have just been recently identified^5–9^ and in ovo transmission was unknown until now. The widespread distribution of ParPgV in red-legged partridges, together with the predominantly mild clinical presentation observed in experimentally inoculated birds, suggests a well-adapted host-virus relationship. This observation raises important questions regarding whether in ovo infection influences host immune responses and predisposes birds to future neurological disease. If birds become infected as embryos, they may develop tolerance, permitting lifelong viral persistence^28^. Importantly, because all study birds were ParPgV-positive prior to the experimental challenge, it remains unclear whether any observed lesions were specifically induced by superinfection or reflected exacerbation of a pre-existing infection. Therefore, while the experimental findings support an association between ParPgV infection and encephalitis, the lack of naive birds due to the high prevalence of ParPgV in source populations preventing the establishment of a truly naïve infection system, pose a certain limitation to the current experimental model. This cautious interpretation is consistent with the broader pegivirus literature, in which detection of viral RNA in affected tissues has not consistently translated into definitive evidence of disease causation^3,29^.

The final stage of this investigation employed SPF chickens as a potential laboratory model. Although the infection was successfully established in these chickens, evidenced by viral RNA in numerous organs, they showed neither clinical manifestations nor significant histopathological changes, and MRI findings were unremarkable. This is in contrast to red-legged partridges, where viral RNA persisted in multiple tissues, including the brain and spleen. The differences in disease expression may stem from variations in immune responses or viral replication dynamics among host species, potentially also involving differences in receptor binding affinity of ParPgV for chicken versus red-legged partridge cells.

To further explore host-dependent immune responses to ParPgV infection, we examined the humoral antibody profiles elicited in red-legged partridges and SPF chickens. The divergent ELISA profiles observed between SPF chickens and red-legged partridges highlight a pronounced host-dependent difference in humoral immune recognition of ParPgV. While experimentally infected SPF chickens mounted a clear, time-dependent antibody response against the ParPgV E2 protein, becoming evident from 14 dpi onwards, red-legged partridges failed to develop detectable serological reactivity at the end of the experiment (21 dpi). Notably, this absence of seroconversion occurred despite the presence of viral RNA and neuropathological lesions in partridges, indicating that lack of detectable antibodies does not equate to absence of infection. This pattern is consistent with pegivirus biology described in mammalian systems, where persistent infection in the natural host frequently proceeds with minimal or absent antibody responses, and where anti-E2 antibodies, when present, are often associated with viral clearance rather than ongoing replication^3,4,30^. In contrast, heterologous or non-natural hosts may exhibit stronger humoral responses, potentially reflecting altered antigen presentation or a lack of virus–host co-adaptation^31^. Accordingly, the robust antibody induction observed in SPF chickens likely represents a non-natural host response to ParPgV infection, whereas the absence of detectable antibodies in red-legged partridges supports the notion that ParPgV has evolved mechanisms to evade or dampen humoral immunity in its natural host, thereby enabling its persistence.

Furthermore, the possibility that congenital infection shapes immune responses and facilitates later neurological disease in red-legged partridges remains a key question. The limited viral replication and absence of neurological lesions observed in SPF chickens, consistent with the findings in grey partridges, support the notion of restricted host permissiveness and suggest a relatively narrow host adaptation of ParPgV outside red-legged partridges. Host-restricted pathogenicity of other flaviviruses, such as West Nile virus, Usutu virus, western equine encephalomyelitis, and St. Louis encephalitis viruses, underscores how some avian species can harbour high viral loads subclinically^32–36^. Additional research is needed to determine if immune factors restrict ParPgV neuroinvasion in chickens.

In conclusion, the identification of an avian pegivirus associated with encephalitis has significant implications for both wildlife health and the broader understanding of pegivirus evolution. Historically, pegiviruses have been regarded as benign, persistent viruses with limited pathogenic potential. However, our findings, along with recent reports of HPgV-1 in human CNS infections, suggest that pegiviruses may have underappreciated neuropathogenic capabilities. Further studies are needed to elucidate the molecular mechanisms of ParPgV neurotropism and its potential interactions with the host immune system. The role of vertical transmission in viral persistence should also be explored, particularly in the context of population dynamics in farmed and wild partridge populations. Future surveillance studies targeting wild partridges and sympatric bird species will be required to assess transmission dynamics and to determine whether ParPgV contributes to neurological disease in wildlife. Additionally, the development of a reliable in vitro culture system for ParPgV would be invaluable for future studies to further unravel the pathogenesis of such new viruses.

Altogether, this study provides experimental evidence linking a pegivirus to encephalitis. While red-legged partridges appear to tolerate persistent infection with limited clinical manifestations, the histopathological and MRI findings indicate that ParPgV can cause significant neurological disease. These findings challenge the notion of pegivirus non-pathogenicity and highlight the need for further research into their role in avian and mammalian hosts.

## Methods

### Farm background and flock management

Since 2017, sporadic neurological signs have been observed in red-legged partridge (*Alectoris rufa*) flocks on two geographically proximate farms in France. These events primarily involved three breeder flocks (designated A, B, and C), each managed as an independent epidemiological unit, composed of birds aged 20–30 weeks, housed in male-female pairs within individual cages. Flock sizes ranged from approximately 13,000 to 30,000 birds. All flocks were routinely vaccinated against Newcastle disease virus (NDV) and managed under standard photostimulation protocols to induce egg laying, which typically began in early February.

### Histological analysis

Tissue samples were preserved in a 4% neutral buffered formaldehyde solution (SAV LP GmbH, Flintsbach, Germany). After fixation, the samples were rinsed in water and subjected to dehydration and subsequently embedded in paraffin. Thin sections, 4 µm in thickness, were prepared from the paraffin blocks using a microtome (Microm HM 360; Microm Laborgeräte GmbH, Walldorf, Germany). Sections were stained with hematoxylin and eosin (H&E) for histological examination.

### Transmission electron microscopy (TEM)

Brain samples preserved in 4% neutral buffered formaldehyde were used for transmission electron microscopy (TEM). The samples were sectioned into 1-mm³ pieces in 0.1 M phosphate buffer (Sigma-Aldrich, Vienna, Austria) at pH 7.2 and maintained at 4 °C for 3 h. The following preparation steps underwent the usual procedure with post fixation steps in a buffered 2.5% Karnovsky solution (pH 7.3) and in a cold phosphate buffered 1% osmium tetroxide solution (pH 7.3, 4 °C, Agar Scientific, UK), with dehydration in a graded ethanol series followed by treatment with propylene oxide (Merck, Darmstadt, Germany) and with embedding in EPON 812 resin (Serva, Heidelberg, Germany). Ultrathin sections were stained with 1% methanolic uranyl acetate (Ted Pella, USA) and lead citrate after Reynolds (Merck, Germany), and examined using a Zeiss TEM 906 electron microscope (Zeiss, Oberkochen, Germany) operating at 80 kV.

### Nucleic acid extraction and construction of an NGS library

Two next-generation sequencing (NGS) experiments were performed: the first on brain samples from clinically affected red-legged partridges during field outbreaks, and the second on partridge embryos. Total RNA was extracted from brain and embryo homogenates with the RNeasy Plus Mini Kit (Qiagen, Hilden, Germany), followed by genomic DNA removal using RNase-free DNase I (Qiagen) (unless otherwise stated, all reagents and kits were used according to the manufacturers’ protocols). Ribosomal RNA was depleted prior to library preparation using the NEBNext rRNA Depletion Kit v2 (New England Biolabs, Frankfurt am Main, Germany). Sequencing libraries were constructed with the NEBNext® Ultra™ II RNA Library Prep Kit for Illumina (New England Biolabs) and sequenced on the Illumina NextSeq 2000 platform in paired-end 150 bp mode (PE150) at the Vienna BioCenter Core Facilities GmbH (Next Generation Sequencing Facility, Vienna, Austria).

### Analysis of NGS-data obtained from NextSeq

In the first NGS experiment, three brain samples, representing each flock, were processed in a single sequencing run. Raw reads were imported into CLC Genomics Workbench 23 (https://digitalinsights.qiagen.com), trimmed to remove low-quality bases and adaptor sequences, and assembled de novo using the Microbial Genomics Module (default settings). Assembled contigs were screened for candidate viruses using a local BLASTN search against a database of complete viral genomes downloaded from NCBI on July 11, 2022 (-evalue 1e-05 -max_target_seqs 1). For each contig, only the top match was retained. In the second experiment, two partridge embryo samples were sequenced in separate runs. Reads were trimmed using Cutadapt (v4.9) with parameters -a AGATCGGAAGAGC -A AGATCGGAAGAGC -m 20, then imported into CLC Genomics Workbench 24 and assembled de novo as above. Contigs were screened by BLASTN against a custom database of avian pegivirus sequences and the complete genome of the ParPgV-A strain initially derived in this study. Matching contigs were used in the reconstruction of the partial genome sequence of ParPgV-C.

### Establishing the complete genome sequence

The complete genomic sequence was established using a modified Sequence-Independent Single Primer Amplification (SISPA) protocol^37^. Briefly, total RNA from outbreak brain homogenates was reverse-transcribed using the RevertAid First Strand cDNA Synthesis Kit (Thermo Fisher Scientific, Waltham, MA, USA) and 2 µM SISPA-N primer (primers and thermal cycling conditions are listed in Supplementary Table 1). cDNA was amplified with LongAmp® Taq 2X Master Mix (New England Biolabs) and 2 µM SISPA primer, and purified with AMPure Beads XP (Beckman Coulter, Krefeld, Germany) at a bead-to-cDNA ratio of 0.8:1.

Pegivirus-specific primers (Supplementary Table 1) were designed based on NGS-derived contigs and aligned to avian pegivirus sequences from GenBank. PCRs were performed with LongAmp® Taq 2X Master Mix and 0.4 µM primers. Amplicons of expected sizes were excised and purified from agarose gels using the QIAquick Gel Extraction Kit (Qiagen). Purified fragments were cloned into the TOPO-TA vector (Invitrogen, Thermo Fisher Scientific) following the manufacturer’s instructions. From each PCR, at least three independent positive clones were sequenced by Sanger method (LGC Genomics, Berlin, Germany) using M13 and pegivirus-specific primers (Supplementary Table 1). Consensus sequences from ≥3 independent clones were assembled using Accelrys Gene version 2.5 (Accelrys, San Diego, CA, USA). Complete genome sequences of ParPgV-A and ParPgV-C strains were submitted to the NCBI database under accession numbers PV472371 and PV472372, respectively.

### Genome and phylogenetic analyses

Predicted proteins within the viral polyprotein were identified using HMMER Hmmscan search against the Pfam protein database (HmmerWeb version 2.43) and by comparison to the Goose Pegivirus-1 polyprotein. Complete polyprotein sequences of all known avian origin pegiviruses were aligned using the MUSCLE method within the MegAlign Pro module of Lasergene v18.0.1 software (DNASTAR, Madison, WI, USA) with default settings. Signal peptide prediction and cleavage site identification were performed using SignalP 6.0 (SignalP 6.0 - DTU Health Tech - Bioinformatic Services)^38^ with the organism parameter set to “other.” Phylogenetic analyses were conducted based on the NS3 and NS5B regions of the viral polyprotein. A total of 27 pegivirus polyprotein sequences from mammalian and avian origins were downloaded from the NCBI protein database. Amino acid alignments were generated using MUSCLE as implemented in MegAlign Pro (Lasergene v18.0.1) with default parameters. Conserved blocks were identified using GBlocks (https://ngphylogeny.fr implementation), with default settings, retaining positions present in a majority of sequences, requiring ≥85% sequence presence at flanks, permitting up to eight consecutive non-conserved sites, enforcing a minimum block length of 10, and excluding all gap-containing positions. Phylogenetic trees were inferred using the Maximum Likelihood method (RAxML) with 100 bootstrap replicates (as implemented in MegAlignPro v18.0.1.5) under the default protein model, PROTCAT-BLOSUM62 with empirical amino-acid frequencies (Lasergene v 18.0.1). Distance matrices (NS3 region; 544 amino acids) were calculated using uncorrected pairwise distances and global gap removal in MegAlign Pro (Lasergene v 18.0.1).

### Detection of ParPgV RNA by real-time qRT-PCR

Total RNA was extracted from organ tissues with the RNeasy Mini Kit (Qiagen). Real-time RT-PCR assays targeting the conserved 5′-UTR region of the ParPgV genome were performed using the AriaMx Real-Time PCR System and the Brilliant III Ultra-Fast QRT-PCR Master Mix (Agilent Technologies, Vienna, Austria). Each 20 µL reaction contained 2 µL RNA template, 0.5 µM primers, and 0.2 µM probe (primer sequences and PCR conditions are provided in Supplementary Table 1).

Fluorescence signals were analysed with Agilent AriaMx software (version 1.7) with manual threshold settings. A Ct cutoff of 38 was applied^39^. No-template controls were included to monitor contamination. Absolute quantification of viral load in animal samples was performed using a standard curve generated from in vitro transcribed 5′-UTR RNA cloned into a pCR®4-TOPO vector (Invitrogen). RNA transcription was carried out with a MAXIscript T7 kit (Thermo Fisher Scientific) after PCR amplification using the OneStep RT-PCR Kit (Qiagen) and specific primers (Supplementary Table 1). DNA templates were removed with the TURBO DNA-free Kit (Thermo Fisher Scientific). Viral RNA copies/µL were calculated based on RNA concentration and molecular weight.

### Detection of ParPgV-specific RNA by RNAscope

Custom RNAscope probes were designed and provided by Bio-Techne (Dublin, Ireland) to target the highly conserved region at the 5′ end and the NS3 regions of the ParPgV genome, based on the consensus sequences of ParPgV-A nucleotide 2-949 (5’-UTR, signal sequence, and partial E1 region) and ParPgV-C nucleotide 5450-6368 (NS4A-NS5A region) (catalogue no. 1333321-C1 and 1331411-C1, respectively). Probes targeting the messenger RNA (mRNA) of the widely expressed housekeeping gene peptidyl-prolyl-isomerase-B in red-legged partridges (*Alectoris rufa* - PPIB; cat. no. 1331421-C1) and chickens (*Gallus gallus* - PPIB; cat. no. 453371) served as positive controls, while a probe targeting bacterial dihydropicolinate reductase (DapB; cat. no. 310043) was used as a negative control. Detection of viral nucleic acid was performed using in situ hybridization (ISH) with the manual RNAscope 2.5 High Definition RED assay (Bio-Techne), following the manufacturer’s protocol. Briefly, deparaffinized brain sections were pre-treated with 1× Target Retrieval solution and RNAscope® Protease Plus solution before hybridization with the target probe. Post-hybridization, the tissue underwent a series of amplification steps using pre-amplifier and amplifier solutions, followed by the application of a chromogenic substrate. Slides were counterstained with hematoxylin. As positive controls, tissue sections from field outbreaks were used, while negative controls included tissue sections from ParPgV-negative specific-pathogen-free (SPF) chickens. Signal scoring was performed according to the manufacturer’s guidelines.

### Recombinant protein production and generation of a polyclonal anti-ParPgV envelope glycoprotein antibody

The partial E2 envelope glycoprotein (amino acids 414–570) of the ParPgV-A strain was expressed in the Bac-to-Bac Baculovirus system (Invitrogen). The coding region was cloned into the pFastBacHT-A vector using the NEBuilder HiFi DNA Assembly Kit (New England Biolabs). PCR amplification used Q5 High-Fidelity DNA Polymerase (New England Biolabs) with conditions listed in Supplementary Table 1.

Recombinant protein was solubilized in lysis buffer (8 M urea, 50 mM NaH2PO4 pH 7.4, 0.5 M NaCl, 20 µg/mL DNase I, 1 mM MgCl_2_, 1 mM PMSF) and purified under denaturing conditions via His-tag affinity chromatography (His GraviTrap™ TALON®; Cytiva, Marlborough, MA, USA). Protein expression and purification were verified by Coomassie-stained SDS-PAGE and Western blotting using anti-polyHis-tag (Sigma-Aldrich) and anti-mouse-HRP secondary antibodies (BioRad Laboratories) (Supplementary Fig. 1). Approximately 0.5 mg of purified E2 protein was delivered to Davids Biotechnologie GmbH (Regensburg, Germany) for rabbit immunization and polyclonal antibody production. The specificity of the rabbit polyclonal antibody was evaluated using a commercial multi-species competitive ELISA (ID Screen® West Nile Competition, IDvet, Grabels, France) targeting conserved Orthoflavivirus E protein epitopes, confirming the absence of detectable cross-reactivity (Supplementary Fig. 2).

### Detection of ParPgV antigen by immunohistochemistry

For immunohistochemistry, 4-µm sections of formalin-fixed paraffin-embedded (FFPE) samples were prepared using a microtome (Microm HM 360) and mounted on positively charged glass slides (Superfrost Plus; Menzel-Gläser, Braunschweig, Germany). Tissue samples from partridges and SPF chickens, including samples from healthy birds and those infected with heterologous agents such as fowl adenovirus or *Histomonas meleagridis*, were included to evaluate the specificity of the polyclonal serum. Slides were dewaxed, rehydrated, and subjected to antigen retrieval in citrate buffer (pH 6.0). Endogenous peroxidase activity was blocked using a 1.5% hydrogen peroxide solution in methanol for 30 min. To prevent nonspecific binding, the sections were incubated with a blocking solution consisting of a 1:10 dilution of normal goat serum (Vector Laboratories, Burlingame, USA) combined with 2% bovine serum albumin (Roche Diagnostics GmbH, Mannheim, Germany) for 60 min at room temperature in a humidified chamber. Primary antibody incubation was carried out overnight at 4 °C using rabbit polyclonal anti-E2 serum at dilutions of 1:100, 1:500, and 1:1000. Additional sections were incubated with phosphate-buffered saline (PBS) instead of the primary antibody and used as negative controls. After washing with PBS, the sections were incubated with a 1:400 dilution of biotinylated anti-rabbit IgG (Vector Laboratories) for 30 min, followed by treatment with the Vectastain ABC Kit (Vector Laboratories) for 60 min. Signal detection used the DAB Substrate Kit for peroxidase (Vector Laboratories). The sections were counterstained with Mayer’s hematoxylin (Merck, Darmstadt, Germany), dehydrated, and mounted under coverslips with Neomount (VWR, Vienna, Austria).

### Field sampling of red-legged partridge flocks for detection of ParPgV

A field screening for ParPgV was conducted across eight red-legged partridge flocks, from eight different farms, including both farms with a documented history of encephalitis and those without prior outbreaks. From each flock, 30 cloacal swabs were collected, comprising samples from 15 female and 15 male birds, corresponding to the number required to detect at least one infected bird with 95% confidence, assuming a 10% design prevalence. Additionally, 30 embryonated eggs were collected from each flock. The yolk and allantoic fluid were extracted from 15 eggs for screening, while the remaining 15 eggs were incubated for 14 days to allow embryo collection for further analysis. Samples were pooled into groups of five for each type of material, and all pools were tested using real-time RT-PCR.

### Experimental in vivo investigations of ParPgV in partridge and chicken hosts

#### Inoculum preparation

Due to unsuccessful attempts to culture the virus in vitro (data not shown), an experimental inoculum was prepared using a filtrate from a ParPgV PCR-positive brain sample collected from flock A. For this purpose, brain tissue was suspended in PBS at a concentration of 20% (wt/vol), supplemented with 1 mg/ml streptomycin and 100,000 IU/ml penicillin. The suspension was homogenized using a T25 Ultra Turrax® (IKA, Staufen, Germany) at 20,000 rpm. The resulting homogenate was clarified by centrifugation at 2000 × *g* for 10 min, and the supernatant was passed through a 0.2 µm filter (Filtropur S 0.2, Sarstedt, Nümbrecht, Germany) to obtain the final filtrate (10^2.760^ viral genome copies/µl).

Following the first in vivo experiment in grey partridges (Study ID: GP), PCR analysis targeting the 260bp-ParPgV NS4A gene (Supplementary Table 1), followed by Sanger sequencing, identified two distinct ParPgV strains—designated ParPgV-A and ParPgV-C (the strain prevalent in flock C)—in the inoculum. A strain specific real-time qPCR targeting the ParPgV NS3 gene was developed to assess the ratios of each strain both in the inocula and samples obtained from the grey partridge experiment (Supplementary Data 2). As ParPgV-C was the predominant strain detected in the organs of inoculated grey partridges (Supplementary Data 2b) and was also highly prevalent in field monitoring samples (Supplementary Data 1), it was selected for the subsequent red-legged partridge (Study ID: RLP) and SPF-chicken (Study ID: SPF) experiments. Consequently, the RLP and SPF in vivo experiments were conducted using a ParPgV-C inoculum (10^2.752^ viral genome copies/µl), derived from a ParPgV PCR-positive brain sample from flock C, and free from ParPgV-A (Supplementary Data 1a), prepared following the same protocol as ParPgV-A.

#### Birds, experimental inoculation, and sampling

Three separate consecutive experiments were conducted, each involving distinct groups of birds: (GP) grey partridges (*Perdix perdix*), (RLP) red-legged partridges (*Alectoris rufa*), and (SPF) SPF chickens (*Gallus gallus*). Grey partridges were sourced as 5-month-old birds from a local supplier in Austria (Mühlböck, Natternbach, Austria). In contrast, embryonated eggs of red-legged partridges (Gibovendeé, Les Herbiers, France) and SPF chickens (VALO BioMedia GmbH, Osterholz-Scharmbeck, Germany) were incubated at the facilities of the Clinic for Poultry Medicine at the University of Veterinary Medicine Vienna, Austria. Upon hatching, birds were housed under controlled conditions with feed and water ad libitum. Experiments were conducted under biosafety level 1 (BSL-1) conditions; SPF chicken studies were performed in isolator units meeting BSL-2 containment standards. Sex was not considered in the experimental design, as birds were obtained as embryonated eggs or from limited sources where sex determination prior to or at hatching is technically challenging and would require additional invasive procedures. Given the exploratory nature of the study and in accordance with the 3Rs principles, inclusion of sex as a variable was not considered essential for addressing the primary study objectives. All procedures were approved by the Ethics and Animal Welfare Committee of the University of Veterinary Medicine, Vienna, in accordance with the University’s guidelines for Good Scientific Practice and authorized by the Austrian Federal Ministry of Education, Science, and Research (ref BMBWF 2022-0.713.294, and Extension 2023-0.430.929), in accordance with current legislation.

Details of the experimental design are summarized in Table 1. Birds were intravenously inoculated with either ParPgV filtrate or PBS as a control. Clinical signs, which included weakness, reluctance to move, lack of avoidance to capture, and closed eyes, were monitored daily throughout the study. At predetermined timepoints, birds were euthanised, and tissue samples were collected for histopathological and molecular analyses. In the grey partridge experiment, tissues included the brain, spleen, liver, and kidney. In the red-legged partridge experiment, additional samples from the bone marrow and caecal tonsils were collected. For the SPF chicken experiment, the thymus and bursa of Fabricius were also sampled in addition to the previously mentioned organs. All collected tissues were preserved in 4% neutral buffered formaldehyde solution (SAV LP GmbH) for histopathological analysis and stored at −80 °C for virological and molecular investigations. Additionally, at different time points, tracheal and cloacal swabs were collected in the grey partridge and red-legged partridge experiments, while only cloacal swabs were collected in the SPF chicken experiment.

**Table 1:** Overview of in vivo experimental infections with ParPgV in grey partridges, red-legged partridges, and SPF chickens

Study ID	Host	Age at inoculation	Group ID	No. Birds	Inoculum (ml)/bird	Route of inoculation	Sequential culling (dpi): no. birds			
							7	10	14	21
GP	Grey partridge (*Perdix perdix*)	5-months old	GP-VG	7	0.2 ParPgV-A filtrate	iv	n.d.	n.d.	2	3
			GP-CG	3	0.2 PBS	iv	n.d.	n.d.	1	1
RLP	Red-legged partridge (*Alectoris rufa*)	5-months old	RLP-VG	14	0.2 ParPgV-C filtrate	iv	4	4	3	3
			RLP-CG	6	0.2 PBS	iv	2	2	1	1
SPF	SPF chicken (*Gallus gallus*)	4-weeks old	SPF-VG	12	0.2 ParPgV-C filtrate	iv	4	n.d.	4	4
			SPF-CG	8	0.2 PBS	iv	2	n.d.	3	3

*dpi* days post inoculation, *iv* intravenous, *PBS* phosphate-buffered saline, *n.d.* not done.

### Magnetic resonance imaging (MRI)

Before euthanasia, birds from the RLP and SPF experiments were transported under deep sedation to the diagnostic imaging unit. Magnetic resonance imaging (MRI) of the head was performed on two ParPgV-inoculated birds and one control bird per time point using a 1.5 Tesla scanner (Magnetom Espree, Siemens Healthineers, Erlangen, Germany). MRI scans were performed at the time points indicated in Table 1 using a 70 mm medium-sized loop coil. Standardized imaging protocols were applied to ensure consistency across all groups and time points, including T2-weighted constructive interference in steady-state (CISS) 3D (TR 6.9 ms, TE 2.97 ms), T1-weighted turbo spin echo (TSE) transverse (TR 907 ms, TE 16 ms, slice thickness 5 mm), T2-weighted turbo inversion recovery (TIR) transverse (TR 4770 ms, TE 65 ms, slice thickness 0.8 mm), and T1-weighted gradient echo (GRE) turbo flash 3D (TR 1720 ms, TE 5.92 ms). The imaging data were anonymized and independently assessed by a European Board-certified radiologist, who performed subjective scoring of pathological changes.

### ParPgV E2 protein-based enzyme-linked immunosorbent assay (ELISA)

ELISA was conducted using microtiter plates (Nunc, Roskilde, Denmark) coated with 100 µl of 0.2 µg ParPgV E2 protein diluted in 0.05 M carbonate buffer (pH 9.4) supplemented with 0.7 M NaCl and an inert protein additive (StartingBlock™ T20 PBS, Pierce Biotechnology, Rockford, USA). Plates were incubated overnight at 4 °C and subsequently washed three times with PBS containing 0.1% Tween-20 (pH 7.4) using an automated plate washer. Serum samples from the RLP and SPF experiments, diluted 1:100 in blocking buffer (StartingBlock™ T20 PBS), were applied in duplicate and incubated for 1 h at room temperature. After three additional washes, 100 µl of 1:10,000 Goat-anti-Galliformes IgY conjugated to horseradish peroxidase (IDvet, Grabels, France) was added to each well and incubated for 1 h at room temperature, followed by a final wash cycle. Colour development was achieved by adding 100 µl of TMB (tetramethylbenzidine) substrate (Calbiochem, Darmstadt, Germany) per well and incubating the plates in the dark for 15 min. The enzymatic reaction was stopped by adding 100 µl of 0.5 M sulfuric acid, and optical density (OD) values were measured at 450 nm. Reported OD values represent the mean of duplicate measurements.

### Detection of ParPgV negative-strand RNA by RT-PCR

Detection of the negative-strand RNA was adapted from Lin et al.^40^ with modifications. A 260-bp fragment of the NS4A region was amplified using OneStep RT-PCR Kit (Qiagen) and cloned into the pCR®4-TOPO vector (Invitrogen). Primer sequences and thermal cycling conditions are provided in Supplementary Table 1. Positive and negative RNA strand controls were generated by in vitro transcription from T3 and T7 promoter sites, respectively, using the MAXIScript T3 and T7 kits (Thermo Fisher Scientific) and the NS4A-pCR®4-TOPO plasmid. DNA templates were removed with the TURBO DNA-free Kit (Thermo Fisher Scientific) and the synthetized RNA was tested for residual DNA contamination by performing PCR in the absence of reverse transcription.

To detect negative-strand RNA in samples, cDNA synthesis was performed using the OmniScript RT Kit (Qiagen) and a chimeric primer consisting of an oligonucleotide tag fused to a virus-specific sequence. Subsequent PCR amplification was conducted with HotStarTaq Master Mix Kit (Qiagen) using the oligonucleotide tag as the forward primer and a ParPgV-specific reverse primer (Supplementary Table 1). Successful detection of negative-strand RNA yielded a 257-bp PCR amplicon.

### Data analysis, statistics and reproducibility

Sample sizes were selected based on prior experience with similar avian virology experiments, considering ethical limitations and feasibility, while ensuring sufficient biological replicates for statistical analysis and reproducibility. Data were recorded in Excel (Office 365) and analysed in Python (version 3.13). Data processing and statistical analyses were performed using pandas, scipy, and matplotlib/seaborn libraries. Serological data were assessed with Table Analyzer^41^. Normality of continuous variables was assessed using the Shapiro–Wilk test. Comparisons of viral load in organs of experimentally inoculated red-legged partridges were performed using a two-way ANOVA (two-sided) with factors organ and time point (dpi), followed by post hoc pairwise comparisons between each infected group and the control group (RLP-CG) within each organ. *P* values were adjusted for multiple comparisons using the Holm method.

Histological, RNAscope in situ hybridization, and immunohistochemical analyses were performed on available biological samples, with sample sizes (*n*) indicated in the corresponding figure legends. These analyses were conducted on tissues previously characterized by histopathology and qPCR, and consistent findings were observed across all examined samples.

### Reporting summary

Further information on research design is available in the Nature Portfolio Reporting Summary linked to this article.

## Supplementary information

Supplementary Information

Description of Additional Supplementary Files

Supplementary Data 1

Supplementary Data 2

Supplementary Movie 1

Supplementary Movie 2

Reporting Summary

Transparent Peer Review file

## Source data

Source Data

## Data Availability

The complete genome sequences of ParPgV-A and ParPgV-C have been deposited in the NCBI GenBank under accession numbers PV472371 and PV472372, respectively. The raw sequencing data from all field samples used as inocula were submitted into the NCBI SRA database, under the BioProject PRJNA1314175. Data under accessions SAMN50922524 and SAMN50922527 correspond to the inoculum used for animal experiment in grey partridges, whereas data with accessions SAMN50922526 and SAMN50922529 are from the inoculum used in animal experiments performed in red-legged partridges and SPF chickens. All other data supporting the findings of this study, including viral load measurements, histopathology evaluations, serological data, statistical source data, primers, probes, cycling conditions, and amino acid identity matrix, are available within the manuscript and its Supplementary files/Source data file. Source data are provided with this paper.

## Code availability

Serological data were assessed with Table Analyzer (Version 388 v1.10), an R Shiny web application publicly available on GitHub (https://github.com/nicola-palmieri/TableAnalyzer)^41^.
