# Supplementary material for: A pegivirus associated with encephalitis in red-legged partridges shows neurotropism across avian species
Source: Nat Commun. 2026 Jun 5;17:7200. doi: 10.1038/s41467-026-73858-8 (PMC13396197; doi:10.1038/s41467-026-73858-8)
Supplement: Supplementary file 1 — Supplementary Information [file 41467_2026_73858_MOESM1_ESM.pdf]

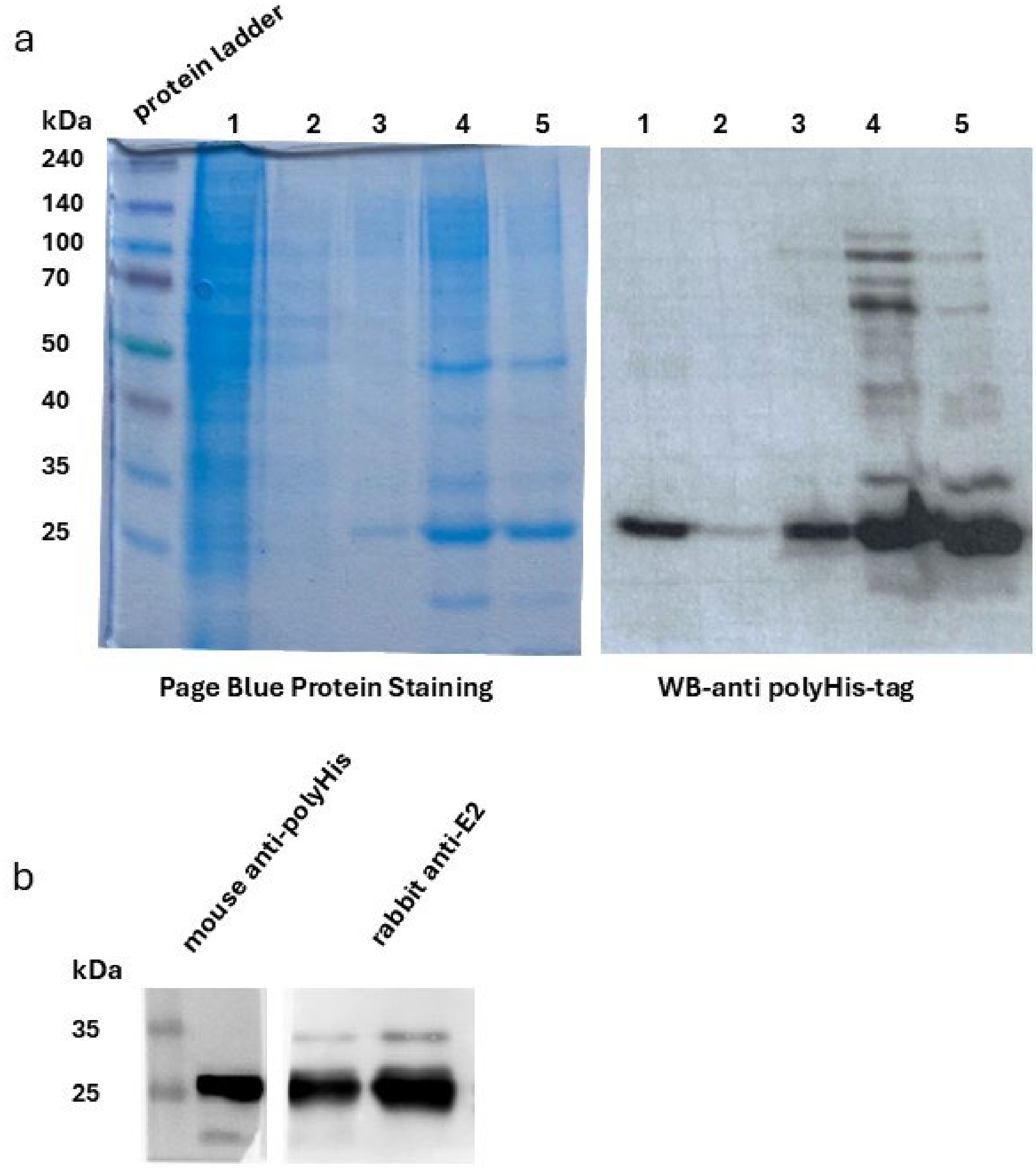

Supplementary Fig. 1. Expression, purification, and detection of recombinant partial ParPgV E2 protein.

(a) Two identical 12% SDS-PAGE gels were run in parallel with the following samples: unpurified E2 protein (lane 1), wash fraction (lane 2), and three elution fractions (lanes 3–5). The left panel shows SDS-PAGE stained with PageBlue Protein Staining Solution (Thermo Scientific), and the right panel shows the corresponding western blot probed with mouse anti-polyHis-tag antibody (1:3000) followed by anti-mouse HRP-conjugated secondary antibody (1:1000).

(b) Western blot of purified E2 protein separated on a 12% SDS-PAGE gel. The left panel shows detection using mouse anti-polyHis-tag antibody (1:3000) with anti-mouse HRP-conjugated secondary antibody (1:1000). The right panel shows detection using rabbit anti-E2 antibody (1:5000) with anti-rabbit HRP-conjugated secondary antibody (1:20,000). Spectra Multicolor Broad Range Protein Ladder (Thermo Scientific) was used in all gels.

a

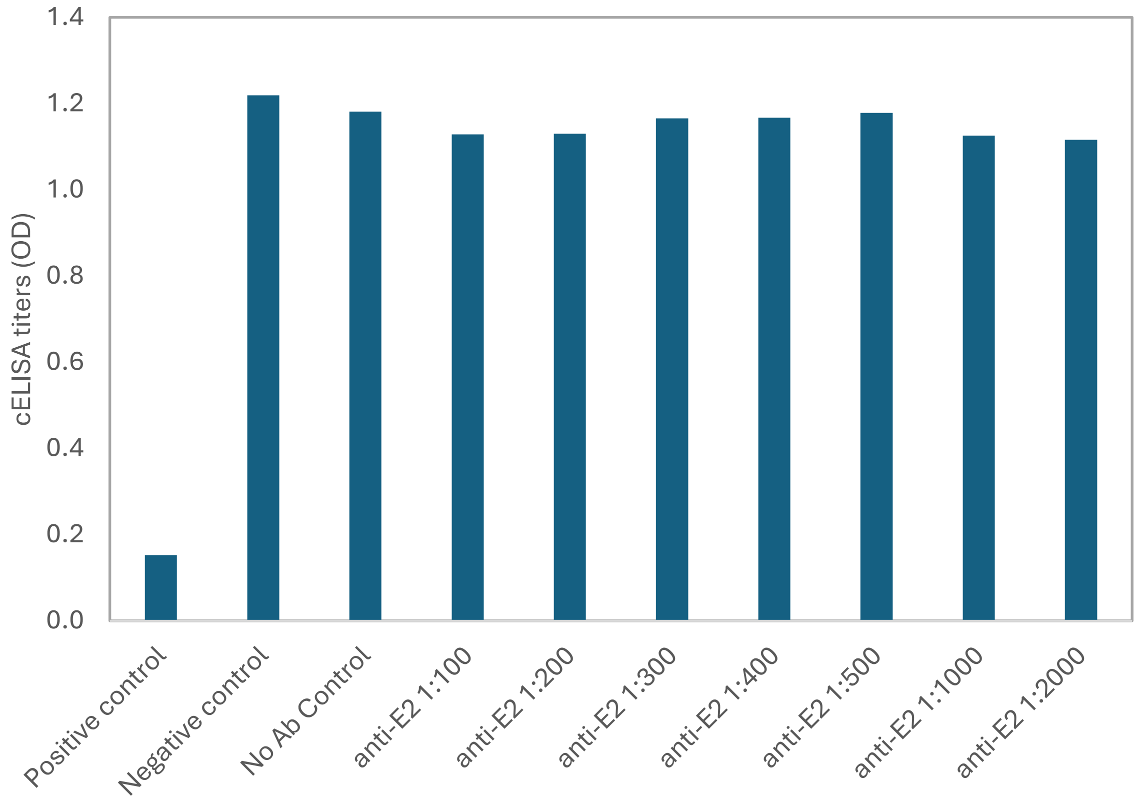

b

Sample	Result (S/N)%	Interpretation
Positive control	12.510	positive
No Ab control	96.883	negative
anti-E2 1:100	92.576	negative
anti-E2 1:200	92.699	negative
anti-E2 1:300	95.611	negative
anti-E2 1:400	95.734	negative
anti-E2 1:500	96.678	negative
anti-E2 1:1000	92.330	negative
anti-E2 1:2000	91.550	negative

Supplementary Fig. 2. Assessment of cross-reactivity of the ParPgV anti-E2 polyclonal antibody using a competitive Orthoflavivirus ELISA.

(a) Competitive ELISA optical density (OD) values obtained for assay controls and serial dilutions of the rabbit polyclonal anti-E2 antibody raised against Partridge pegivirus (ParPgV). Antibody specificity was evaluated using a commercial multi-species competitive ELISA targeting a conserved epitope within the Orthoflavivirus envelope (E) protein (ID Screen® West Nile Competition, IDvet, Grabels, France), which broadly detects antibodies against diverse Orthoflaviviruses, including West Nile, Usutu, Japanese encephalitis, Dengue, Zika, Tick-borne encephalitis, Tembusu, and Bagaza viruses. The anti-E2 antibody was tested at serial dilutions ranging from 1:100 to 1:2000, while the assay conjugate was diluted 1:10 according to the manufacturer's instructions. All measurements, including positive and negative controls, were performed in duplicate, and mean OD values are shown.

(b) Tabulated interpretation of the competitive ELISA results expressed as the sample-to-negative control (S/N) ratio (%), calculated according to the manufacturer's criteria. An S/N ratio $\leq$ 40% was considered positive, values between 40–50% indeterminate, and values > 50% negative. All tested dilutions of the ParPgV anti-E2 antibody yielded S/N ratios within the negative range, comparable to the no-antibody control and clearly distinct from the positive control. Collectively, these results demonstrate an absence of detectable cross-reactivity between the ParPgV anti-E2 antibody and Orthoflavivirus E protein epitopes, supporting the high specificity of the generated polyclonal antibody.

a

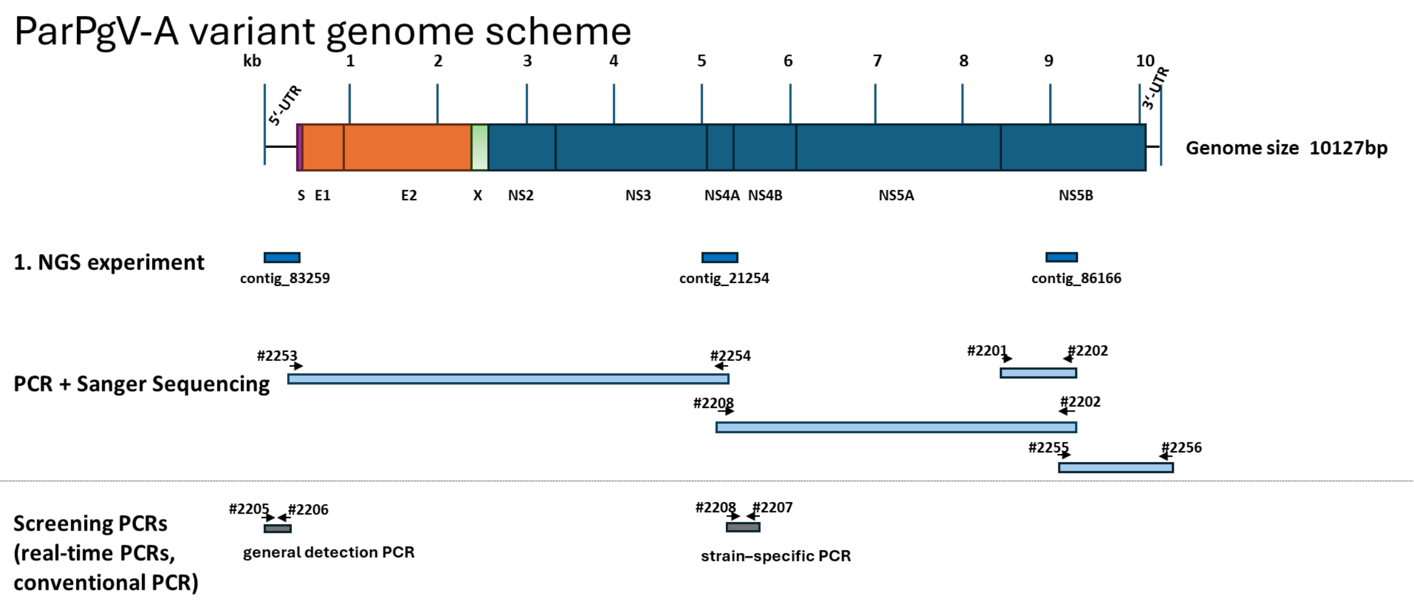

b

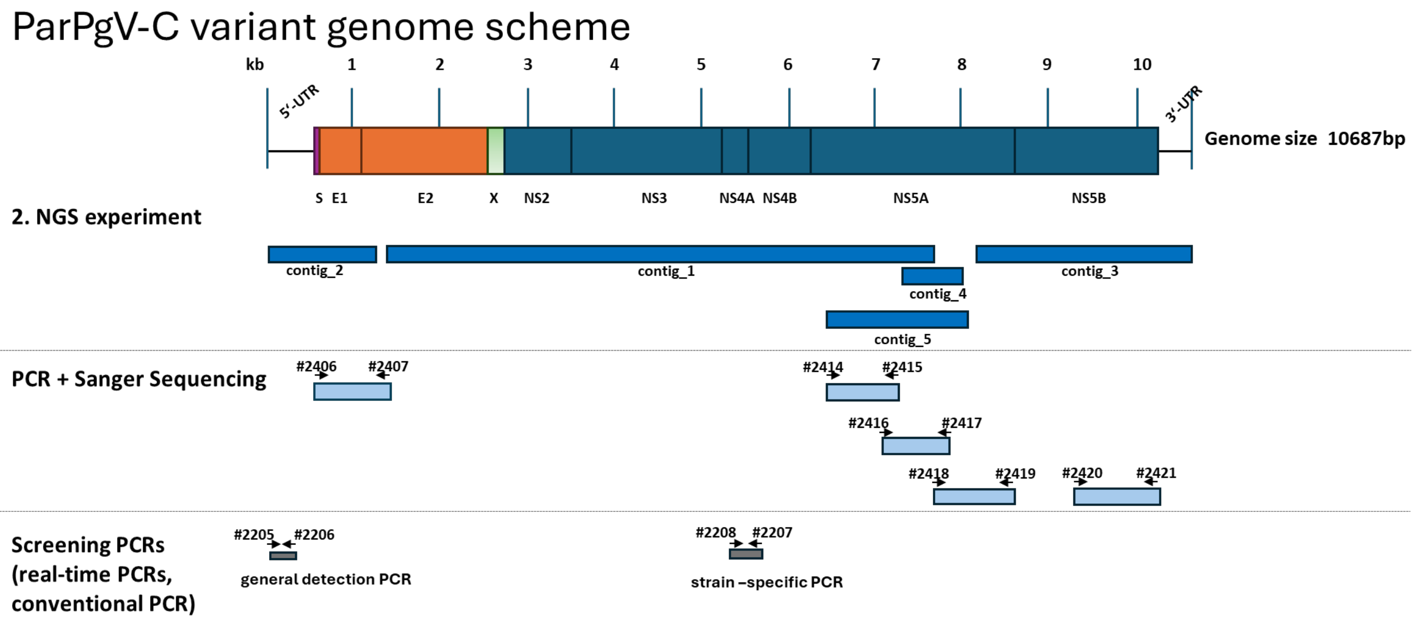

Supplementary Fig. 3. Genome organization and sequencing strategy for two partridge pegivirus (ParPgV) strains.

(a) ParPgV-A strain genome scheme (genome size 10,127 bp). Three contigs were assembled from next generation sequencing (NGS) data (contig_83259, contig_21354, contig_86166), the remaining regions were completed using specific PCRs and Sanger sequencing. General detection and strain-specific PCRs are indicated. All primers are labeled with the ID number, their corresponding sequence and use is listed in the Supplementary table 1.

(b) ParPgV-C strain genome scheme (genome size 10,687 bp). NGS yielded four contigs (contig_1–contig_4), and gap-filling was completed using PCR and Sanger sequencing. General detection and strain-specific PCR targets are indicated. All primers are labeled with the ID number, their corresponding sequence and use is listed in the Supplementary table 1.

Supplementary Table 1. List of primers, probes, and PCR cycling conditions used for the amplification and detection of partridge pegivirus (ParPgV).

Assay	Primer/Probe name[1]	Sequence (5'-3')	Position on ParPgV genome	Ta[2]	Product length	PCR cycling RT[3]	PCR cycling Initial denaturation	PCR cycling cycling conditions	PCR cycling final elongation
genome amplification PCR (ParPgV-A)	Pegi_5.1kb-F (#2253)	TGA CCC CAC CCC AGA CCT C	277:295 (ParPgV-A)	60°C	4.928kb	n.a.[4]	94°C for 3 min	30x 94°C 30s, Ta (Table S1) 30 s, 65°C 50s/1kb PCR product	65°C for 10 min
	Pegi_5.1kb-R (#2254)	CCA GGC ACC CGG TGT AGT TC	5223:5204 (ParPgV-A)						
genome amplification PCR (ParPgV-A)	Pegi260-F (#2208)	GCGGCTTTGGTGAACTACAC	5193:5212 (ParPgV-A)	60°C	4.129kb				
	Pegi814bp-R (#2202)	TCACACTCCTCGCAGACGATGAGGCA	9346:9321 (ParPgV-A)						
Pegivirus screening conventional PCR	Pegi150bp-F (#2205)	TGCACAGTGCTTATAAACTCAG	51:72 (ParPgV-A) / 288:309 (ParPgV-C)	55°C	150bp	50°C for 30 min	95 for 15 min	35X 95°C 30s, Ta (Table S1) 30 s, 72°C 1 min	72°C for 10 min
	Pegi150bp-R (#2206)	GTTCTAACCTTACAGCGCC	181:199 (ParPgV-A) / 437:419 (ParPgV-C)						
Pegivirus screening conventional PCR	Pegi260-F (#2208)	GCGGCTTTGGTGAACTACAC	5193:5212 (ParPgV-A) / 5429:5448 (ParPgV-C)	59°C	260bp				
	Pegi260-R (#2207)	AGCATCCAAGACTCCTTGGC	5452:5433 (ParPgV-A) / 5688:5669 (ParPgV-C)						
Pegivirus screening real-time PCR (5'-UTR real-time RT-PCR)	ParPgV_5UTR-F (#2342)	AGT GCT TAT AAA CTC AGT ACC CTG G	56:80 (ParPgV-A) / 293:317 (ParPgV-C)	n.a.	n.a.	50°C for 15 min	95°C for 3 min	40x 95°C for 5s, 60°C for 20s	n.a.
	ParPgV_5UTR-R (#2343)	GAT GCT TTG TCA AAT CGC GGA TAG	172:149 (ParPgV-A) / 409:386 (ParPgV-C)						
	ParPgV_5UTR-P (#2344)	5'-FAM-TTC CCC AAG GCG GCA ACG GGC TAG GC-BHQ1-3'	85:110 (ParPgV-A) / 322:347 (ParPgV-C)						
ParPgV-A strain screening real-time PCR (NS3 real-time RT-PCR)	ParPgV/A_NS3-F (#2329)	GAA GGG CAC GTT AGT GTG ACT G	5259:5280 (ParPgV-A)						
	ParPgV/A_NS3-R (#2330)	CTT AGA CAC GGT GAT GAG GTG G	5375:5354 (ParPgV-A)						
	ParPgV/A_NS3-P (#2331)	5'-HEX-ACG TGC GTG TCG TGG GAC GCT TGC GT-BHQ1-3'	5316:5341 (ParPgV-A)						
ParPgV-C strain screening real-time PCR (NS3 real-time RT-PCR)	ParPgV/C_NS3-F (#2321)	AAG TGT GAC TGA CGT GGT GAT AG	5506:5528 (ParPgV-C)						
	ParPgV/C_NS3-R (#2322)	GCT GTT TGT GCC AAG TGT ACT G	6540:5619 (ParPgV-C)						
	ParPgV/C_NS3-P (#2323)	5'-FAM-TCC TGG GAC GCT TGC GTC GAC GGG AT-BHQ1-3'	5561:5586 (ParPgV-C)						
genome amplification PCR	Pegi814bp-F (#2201)	GGATTTCGACTGTGACGAGGGAGCAGG	8533:8559 (ParPgV-A)	61°C	814bp	n.a.	94°C for 3 min	30x 94°C 30s, Ta (Table S1) 30 s, 65°C 50s/1kb PCR product	65°C for 10 min
	Pegi814bp-R (#2202)	TCACACTCCTCGCAGACGATGAGGCA	9346:9321 (ParPgV-A)						
genome amplification PCR	Pegi_3'_1.2kb-F (#2255)	GGC TCA TGG TCA ACC CGA AAG G	9153:9174 (ParPgV-A)	60°C	976bp				
	Pegi_3'_1.2kb-R (#2256)	TAG AGT TTT CGC CGG TTC GCC	10148:10128 (ParPgV-A)						
SISPA	SISPA	GTTGGAGCTCTGCAGTC	not applicable		not applicable	n.a.	94°C for 3 min	30x 94°C 30s, 55°C 30s, 65°C 5 min	65°C for 10 min
SISPA	SISPA-N	GTTGGAGCTCTGCAGTCATCNNNNNN	not applicable		not applicable	25°C 10 min, 42°C 1h	n.a	n.a.	n.a.
genome amplification PCR (ParPgV-C)	PegiC_Nterm-884bp-F1 (#2406)	CAGTCTTCAGGGTTTTCGTG	553:572 (ParPgV-C)	57°C	884bp	n.a.	94°C 3 min	30x 94°C 30s, Ta (Table S1) 30 s, 65°C 50s/1kb PCR product	65°C 10 min
	PegiC_Nterm-884bp-R1 (#2407)	AGTAGCCCGATTAGTGTATTCC	1528:1507 (ParPgV-C)						
genome amplification PCR (ParPgV-C)	PegiC_6424-F (#2414)	CACTTTTGCACTGCCAATC	6426:6444 (ParPgV-C)	58°C	835bp				
	PegiC_7260-R (#2415)	GGTGTCAGTGGTAACCTTTC	7260:7241 (ParPgV-C)						
genome amplification PCR (ParPgV-C)	PegiC_7076-F (#2416)	GTGGAGTCTCCCGTGATATG	7076:7095 (ParPgV-C)	58°C	850bp				
	PegiC_7925-R (#2417)	CCCACACTTTCTCTAGGTCG	7925:7906 (ParPgV-C)						
genome amplification PCR (ParPgV-C)	PegiC_7705-F (#2418)	ACTCATGACGTACAAAGCAG	7705:7724 (ParPgV-C)	58°C	948bp				
	PegiC_8652-R (#2419)	ACTCCACTCCAGATGTAGC	8652:8634 (ParPgV-C)						
genome amplification PCR (ParPgV-C)	PegiC_9389-F (#2420)	CTCATGGTCAACCCGAAAG	9389:9407 (ParPgV-C)	58°C	994bp				
	PegiC_10382-R (#2421)	CCACGGCGTACTTGTATTC	10382:10364 (ParPgV-C)						
sequencing primer (ParPgV-A)	Pegi-5kb-819F	TGGACTTCTTTGTCGGCTTG	2083:2102 (ParPgV-A)	n.a.	n. a.	n.a.	n.a.	n.a.	n.a
sequencing primer (ParPgV-A)	Pegi-5kb-C-term-238R	CTGAACACAGGTGCCAAAAG	3410:3391 (ParPgV-A)	n.a.	n. a.	n.a.	n.a.	n.a.	n.a
sequencing primer (ParPgV-C)	PegiC_NS3-F	CCACACTGAAATCAGATGGG	6288:6307 (ParPgV-C)	n.a.	n. a.	n.a.	n.a.	n.a.	n.a
sequencing primer (ParPgV-C)	PegiC_NS5A	ACCGTTGGACTTTATCCTGC	8872:8853 (ParPgV-C)	n.a.	n. a.	n.a.	n.a.	n.a.	n.a
sequencing primer	PegiA+C_NS5A-F	CCTATCAACGTGCTGCACAC	7065:7084 (ParPgV-A) /7302:7321 (ParPgV-C)	n.a.	n. a.	n.a.	n.a.	n.a.	n.a
sequencing primer (ParPgV-C)	PegiC_NS5A-2R	CTAAGTCGTTCTCATCAACGC	7913:7893 (ParPgV-C)	n.a.	n. a.	n.a.	n.a.	n.a.	n.a
sequencing primer	NS4B-F	TCATTGACTGGGCGTTAAGC	6136:6155	n.a.	n. a.	n.a.	n.a.	n.a.	n.a
sequencing primer	NS4B-R	GTTGACACCTTACACAGTGGA		n.a.	n. a.	n.a.	n.a.	n.a.	n.a
Gibson assembly cloning of patial Pegi_A_E2 into pFastBac HT-A (Baculovirus Expression system)	Pegi_A_E2_fwd	acctgtattttcagggcgccGTGCACACTTGCGTGGTAG	1548:1566 (ParPgV-A)	63°C	474bp	n.a.	98°C 1 min	35x 98°C 10s, 63°C 30s, 72°C 35s	72°C 2min
	Pegi_A_E2_rev	ctttgaattccggatccatTCTGTTGATGTGAACGAGC	2003:2021 (ParPgV-A)						
	pFastBacHT-A_fwd	atg gat ccg gaa ttc aaa g	not applicable	60°C	4856bp			35x 98°C 10s, 60°C 30s, 72°C 2.5 min	
	pFastBacHT-A_rev	ggc gcc ctg aaa ata cag	not applicable						
negative RNA strand assay	PegiC260_negStrand_tag_RT	ACT GAT CGT GGA CTG **GCC TTA GTG AAC TAC AC**	5432:5448	n.a.	n.a.	37°C 1h	n.a.	n.a.	n.a.
	TagPrimer_LNA_neg_strand	AC(T)GA(T)CG(T)G(G)ACTG	not applicable	50°C	257bp	n.a.	95°C 15 min	40x 94°C 30s, 50°C 30s, 72°C 1 min	72°C 10 min
	PegiC260_negStrand_R	AGCATCCATGACTCCTTG	5688:5671 (ParPgV-C)						
positive RNA strand assay	PegiC_260_posStrand_tag_RT	ACG TGC ATC GAC ATG **GTT ATT GCA CTA GCC CAA**	5730:5713 (ParPgV-C)	n.a.	n.a.	37°C 1h	n.a.	n.a.	n.a.
	TagPrimer_LNA_pos_strand	ACG(T)GCA(T)CG(A)C(A)TG	not applicable	50°C	310bp	n.a.	95°C 15 min	40x 94°C 30s, 50°C 30s, 72°C 1 min	72°C 10 min
	PegiC_260_posStrand_R	TAG TGA ACT ACA CCG GTT G	5436:5454 (ParPgv-C)						

Footnotes: [1] F/fwd - Foward; R/rev - Reverse; P - Probe; # - guiding number for Supplementary Fig. 1. [2] Ta - Annealing Temperature. [3] RT- Reverse Transcription; [4] - n.a. Not Applicable.

Supplementary Table 2. Predicted viral proteins of ParPgV-A and ParPgV-C, with Pfam domain matches and genome positions. Predicted mature proteins within the viral polyprotein were identified using HMMER Hmmscan search against the Pfam protein database (HmmerWeb version 2.43) and by comparison to the Goose Pegivirus-1 polyprotein.

ParPgV strain	protein	description	Pfam match (Family Id; accession; description; polyprotein start-aa_end-aa)	amino acid position (polyprotein)	nucleotide position (genome)
A	E1	envelope glycoprotein	HCV_env; PF01539; Hepatitis C virus envelope glycoprotein E1; start-28_end-210	20-211	369:942
C			HCV_env; PF01539; Hepatitis C virus envelope glycoprotein E1; aa28_aa209		605:1178
A	E2	envelope glycoprotein	GBV-C_env; PF12786; GB virus C genotype envelope; aa390_aa586	212-679	945:2346
C			GBV-C_env; PF12786; GB virus C genotype envelope; aa361_aa586		1181:2582
A	X	additional glycoprotein	n.d.	680-750	2349:2559
C					2585:2795
A	NS2	non-structural protein NS2	HCV_NS2; PF01538; Hepatitis C virus non-structural protein NS2; aa768_aa963	751-986	2562:3177
C					2798:3503
A	NS3	protease and helicase activity	Peptidase_S29; PF02907; Hepatitis C virus NS3 protease; aa1015_aa1163<br>Flavi_DEAD; PF07652; Flavivirus DEAD domain; aa1181_aa1323	987-1613	3270:5148
C			Peptidase_S29; PF02907; Hepatitis C virus NS3 protease; aa1015_aa1163<br>Flavi_DEAD; PF07652; Flavivirus DEAD domain; aa1181_aa1323<br>DEAD; PF00270; DEAD/DEAH box helicase; aa1168_aa1319		3506:5384
A	NS4A	non-structural protein NS4A	n.d.	1614-1679	5151:5346
C					5387:5582
A	NS4B	non-structural protein NS4B	HCV_NS4b; PF01001; Hepatitis C virus non-structural protein NS4b; aa1686_aa1875	1680-1925	5349:6084
C					5585:6320
A	NS5A	non-structural protein NS5A	HCV_NS5a_1a; PF08300; Hepatitis C virus non-structural 5a zinc finger domain; aa1960_aa2010	1926-2694	6087:8391
C					6323:8627
A	NS5B	RNA-dependent RNA polymerase	RdRP_3; PF00998; Viral RNA-dependent RNA polymerase; aa2697_aa3187<br>RdRP_1; PF00680; Viral RNA-dependent RNA polymerase; aa2737_aa3103	2695-3258	8394:10164
C			RdRP_3; PF00998; Viral RNA-dependent RNA polymerase; aa2697_aa3186<br>RdRP_1; PF00680; Viral RNA-dependent RNA polymerase; aa2738_aa3077		8630:10319

Abbreviations: n.d., not determined; Pfam, Protein family database.

Supplementary Table 3. Percent amino acid identity matrix for the NS3 region (544 amino acids) among partridge pegivirus strains and related avian-origin pegiviruses. Pairwise identities were calculated using uncorrected pairwise distances and global gap removal. Lower left values represent % identity. The matrix highlights 97.98% amino acid identity between ParPgV-A and ParPgV-C, and 59.38–77.39% identity to other avian-origin pegiviruses.

Number of sequences:	29
Lower left values:	%Identity
Distance metric:	Uncorrected Pairwise Distance
Gap treatment:	Global gap removal
Residues considered:	544

	**ParPgV-C**	**ParPgV-A**	WPR17472_Fernbird_pegivirus_1	QUE43477_Goose_pegivirus_1_GPgV-1	QVG71836_Leucosticte brandti pegivirus	QVG71835_Passer montanus pegivirus	UCJ00138_Pin virus	QUE43478_Goose pegivirus-2 GPgV-2	QVG71838_Montifringilla taczanowskii pegivirus	ANO81671_Porcine pegivirus PPgV	AZY88695_Dolphin pegivirus DPgV	AAA96964_*Pegivirus hominis* genotype 2 HPgV-gt2	QKY59672_Human pegivirus genotype 1 HPgV-gt1	AHH32934_Simian pegivirus SPgVkrc_RC29	AHH32962_Simian pegivirus SPgVkob_OB23	QOI90119_Southwest bike trail virus-2 SOBV-2	T08839_Marmoset hepatitis GB Virus A	AAC55983_*Pegivirus platyrrhini* _SPgV-A	AGK41011_Bat pegivirus PDB-737B BPgV	AGK41019_Bat pegivirus PDB-491.2 BPgV	AGI04301_*Pegivirus caballi* _EPgV	AGH70217_Pegivirus equi_TDAV	AGK41018_*Pegivirus sturnirae* _BPgV-I	AEA73241_Bat GB-like virus JFD-2011	ADK12629_*Pegivirus pteropi* _GB virus-D	ALE27082_*Pegivirus columbiaense* _HHPgV	AGK41006_*Pegivirus scotophili* _BPgV-G	AGK41010_*Pegivirus carolliae* _BPgV-F	AGJ71780_*Pegivirus neotomae* _RPgV
**ParPgV-C**																													
**ParPgV-A**	**97.98**																												
WPR17472_Fernbird_pegivirus_1	**76.84**	**76.10**																											
QUE43477_Goose_pegivirus_1_GPgV-1	60.29	59.38	61.76																										
QVG71836_Leucosticte brandti pegivirus	67.46	66.91	65.99	60.66																									
QVG71835_Passer montanus pegivirus	67.65	66.91	65.99	60.66	85.85																								
UCJ00138_Pin virus	**75.92**	**75.37**	84.01	62.13	66.73	65.07																							
QUE43478_Goose pegivirus-2 GPgV-2	**77.39**	**76.84**	83.27	62.50	67.46	66.91	86.03																						
QVG71838_Montifringilla taczanowskii pegivirus	**76.10**	**75.55**	80.88	62.32	68.93	67.65	83.27	84.56																					
ANO81671_Porcine pegivirus PPgV	50.00	49.08	48.90	51.84	49.26	49.82	49.08	49.45	50.18																				
AZY88695_Dolphin pegivirus DPgV	50.74	50.37	49.82	53.31	50.37	50.74	49.45	49.45	50.37	55.70																			
AAA96964_*Pegivirus hominis* genotype 2 HPgV-gt2	48.53	47.79	50.18	51.47	52.39	51.29	48.90	48.90	49.63	56.07	54.96																		
QKY59672_Human pegivirus genotype 1 HPgV-gt1	48.35	47.61	50.37	51.65	52.57	51.65	48.90	48.90	49.63	56.43	54.96	98.90																	
AHH32934_Simian pegivirus SPgVkrc_RC29	48.16	47.61	50.00	52.39	51.29	49.63	50.00	49.45	49.82	58.27	55.88	82.54	82.54																
AHH32962_Simian pegivirus SPgVkob_OB23	48.16	47.61	50.00	52.21	50.74	49.08	49.26	48.90	50.18	56.80	54.78	84.01	83.82	90.44															
QOI90119_Southwest bike trail virus-2 SOBV-2	49.82	49.08	50.92	54.23	52.39	53.86	50.37	51.65	51.29	54.04	54.41	67.28	67.65	67.83	66.73														
T08839_Marmoset hepatitis GB Virus A	50.18	49.63	50.37	52.02	50.37	51.47	49.82	50.74	50.55	55.51	54.96	66.54	66.54	68.38	68.93	75.18													
AAC55983_*Pegivirus platyrrhini* _SPgV-A	49.63	49.08	49.26	52.21	50.92	50.00	49.45	49.63	50.74	53.31	53.31	67.46	67.65	67.65	68.93	73.71	82.35												
AGK41011_Bat pegivirus PDB-737B BPgV	50.55	49.63	51.10	53.68	51.47	52.76	50.55	51.10	52.76	56.43	54.41	68.38	68.38	68.38	68.93	75.55	78.31	76.65											
AGK41019_Bat pegivirus PDB-491.2 BPgV	48.90	48.16	51.29	53.31	52.02	51.65	49.45	50.55	52.21	55.88	55.15	69.30	69.67	69.49	70.04	73.71	76.47	77.57	84.38										
AGI04301_*Pegivirus caballi* _EPgV	49.45	49.08	49.45	54.96	49.08	50.18	49.08	50.00	51.65	56.62	57.35	55.33	55.15	55.51	54.23	54.60	54.23	52.94	56.25	56.25									
AGH70217_*Pegivirus equi* _TDAV	49.26	48.90	49.63	54.96	49.26	50.55	48.35	50.55	50.74	57.54	57.54	54.60	54.41	56.43	54.96	54.04	54.04	53.13	54.23	54.96	63.05								
AGK41018_*Pegivirus sturnirae* _BPgV-I	51.84	50.74	50.00	54.60	49.26	49.26	50.37	50.92	50.74	58.09	55.70	57.72	57.72	59.01	59.93	61.58	61.03	59.56	60.11	59.93	56.25	57.17							
AEA73241_Bat GB-like virus JFD-2011	49.08	48.16	48.16	52.02	50.18	50.74	48.71	49.26	49.08	58.82	54.96	58.64	58.27	60.29	60.11	57.72	58.82	56.43	59.74	59.19	57.17	57.17	60.29						
ADK12629_*Pegivirus pteropi* _GB virus-D	48.71	47.98	48.35	53.13	49.63	50.18	48.90	48.90	48.53	59.93	56.99	60.11	59.74	62.13	60.66	59.93	58.27	56.80	61.03	60.85	59.19	57.72	63.05	76.47					
ALE27082_*Pegivirus columbiaense* _HHPgV	42.46	41.91	41.54	42.46	42.83	42.65	40.44	42.10	43.20	41.73	43.01	45.59	45.22	45.40	46.88	42.28	43.01	41.73	43.75	43.38	42.28	42.10	44.67	41.91	43.20				
AGK41006_*Pegivirus scotophili* _BPgV-G	46.14	45.59	47.24	50.18	46.69	46.88	48.35	46.69	48.53	47.79	48.35	47.79	47.79	47.43	46.88	48.90	47.24	47.79	50.74	49.82	51.84	47.79	47.79	46.69	48.16	48.16			
AGK41010_*Pegivirus carolliae* _BPgV-F	47.43	46.69	47.61	49.63	47.06	47.24	47.98	48.53	49.82	49.63	46.88	49.82	49.82	49.45	50.37	49.63	48.90	48.16	50.74	49.45	51.10	48.53	47.61	47.98	49.82	47.98	64.89		
AGJ71780_*Pegivirus neotomae* _RPgV	45.04	44.30	46.14	50.55	48.53	47.24	47.24	46.51	47.43	48.90	48.16	50.37	49.82	50.92	51.10	50.18	47.61	48.90	51.29	51.29	50.55	50.74	49.26	48.35	51.10	47.43	63.05	63.42	

Supplementary Table 4. Detection of ParPgV negative-strand RNA in tissues from experimentally inoculated birds and embryonated eggs.

	**Brain**	**Liver**	**Kidney**	**Spleen**	**Bone Marrow**	**Cecal Tonsil**	**Embryo**	**Egg Yolk**	**Allantoic Fluid**
Red-legged partridge (RLP-VG)	+	-[1]	+	-[1]	-[1]	+	n.a.	n.a.	n.a.
Red-legged partridge (RLP-CG)	+	-	+	-	-	-	n.a.	n.a.	n.a.
Grey partridge (GP)	+	n.d.	n.d.	n.d.	n.d.	n.d.	n.a.	n.a.	n.a.
SPF Chicken (SPF)	n.d.[2]	+	+	+	+	+	n.a.	n.a.	n.a.
Embryonated eggs	n.a.	n.a.	n.a.	n.a.	n.a.	n.a.	+	+	-[1]

Abbreviations: +, positive; -, negative; n.a., not applicable; n.d., not done.
Footnotes: [1] inconclusive; [2] not done due to low viral load.
