# Supplementary material for: A pegivirus associated with encephalitis in red-legged partridges shows neurotropism across avian species
Source: Nat Commun. 2026 Jun 5;17:7200. doi: 10.1038/s41467-026-73858-8 (PMC13396197; doi:10.1038/s41467-026-73858-8)
Supplement: Supplementary file 2 — Description of Additional Supplementary Files [file 41467_2026_73858_MOESM2_ESM.pdf]

Description of Additional Supplementary Files

Supplementary Movie 1. Red-legged partridge (*Alectoris rufa*) from a field outbreak exhibiting neurological signs consistent with central nervous system involvement. Clinical manifestations include apathy, torticollis, ataxia, and progression to prostration. These observations support the presence of neurologic dysfunction associated with natural infection.

Supplementary Movie 2. Grey partridges (*Perdix perdix*) from the experimental infection study (GP experiment), showing both non-inoculated birds and virus-inoculated individuals. At 4 days post-inoculation (dpi), inoculated birds display a transient reduction in flying ability compared to non-inoculated birds, indicating mild and reversible impairment of motor function following infection.

Supplementary Data 1

1a. Real-time RT-PCR detection of Partridge pegivirus (ParPgV) RNA in field swab samples. Cycle threshold (Ct) values obtained from real-time RT-PCR analyses performed on swab samples collected during field monitoring. Viral RNA was detected using a general ParPgV assay targeting the conserved 5′ untranslated region (5′-UTR), alongside strain-specific assays targeting the NS3 region of ParPgV-A and ParPgV-C. Reactions were carried out on the AriaMx Real-Time PCR System using the Brilliant III Ultra-Fast QRT-PCR Master Mix (Agilent Technologies, Vienna, Austria). Each 20 µL reaction contained 2 µL RNA template, 0.5 µM of each primer, and 0.2 µM probe. Primer sequences and amplification conditions are provided in Supplementary Table 1. Ct values are reported as raw data; non-detected reactions are indicated accordingly.

1b. Real-time RT-PCR detection of Partridge pegivirus (ParPgV) RNA in egg-derived samples. Cycle threshold (Ct) values obtained from real-time RT-PCR analyses performed on egg-derived samples collected during field monitoring. Detection included a general ParPgV assay targeting the conserved 5′ untranslated region (5′-UTR) and strain-specific assays targeting the NS3 region of ParPgV-A and ParPgV-C. Amplifications were performed using the AriaMx Real-Time PCR System and the Brilliant III Ultra-Fast QRT-PCR Master Mix (Agilent Technologies, Vienna, Austria). Each 20 µL reaction comprised 2 µL RNA template, 0.5 µM primers, and 0.2 µM probe. Primer sequences and PCR conditions are detailed in Supplementary Table 1. Ct values are shown as generated, with non-detections indicated where applicable.

Supplementary Data 2

2a. Real-time RT-PCR characterization of ParPgV inocula used in the *in vivo* experiments. Cycle threshold (Ct) values obtained from real-time RT-PCR analyses of viral inocula used for the *in vivo* experiments. Viral RNA was detected using a general ParPgV real-time RT-PCR assay targeting the conserved 5′ untranslated region (5′-UTR), together with strain-specific assays targeting the NS3 region of ParPgV-A and ParPgV-C. Reactions were performed on the AriaMx Real-Time PCR System using the Brilliant III Ultra-Fast QRT-PCR Master Mix (Agilent Technologies, Vienna, Austria). Primer sequences and PCR conditions are provided in Supplementary Table 1. Ct values are reported as raw data.

2b. Real-time RT-PCR detection of ParPgV RNA in organ samples from the grey partridge (GP) experiment. Cycle threshold (Ct) values obtained from real-time RT-PCR analyses of organ samples collected from grey partridges experimentally inoculated with ParPgV. Viral RNA detection was performed using a general ParPgV assay targeting the conserved 5′ untranslated region (5′-UTR) and strain-specific assays targeting the NS3 region of ParPgV-A

and ParPgV-C. Amplifications were carried out on the AriaMx Real-Time PCR System using the Brilliant III Ultra-Fast QRT-PCR Master Mix (Agilent Technologies, Vienna, Austria). Primer sequences and cycling conditions are detailed in Supplementary Table 1. Ct values are shown as generated. Non-detections are indicated where applicable.

2c. Real-time RT-PCR detection of ParPgV RNA in swab and blood samples from the grey partridge (GP) experiment. Cycle threshold (Ct) values obtained from real-time RT-PCR analyses of swab and blood samples collected during the GP experimental infection study. Viral RNA was assessed using a general ParPgV real-time RT-PCR assay targeting the conserved 5′ untranslated region (5′-UTR), alongside strain-specific assays targeting the NS3 region of ParPgV-A and ParPgV-C. Reactions were performed using the AriaMx Real-Time PCR System and the Brilliant III Ultra-Fast QRT-PCR Master Mix (Agilent Technologies, Vienna, Austria). Primer sequences and PCR conditions are provided in Supplementary Table 1. Ct values are reported as raw data, with non-detected samples indicated accordingly.
