# Supplementary material for: A pegivirus associated with encephalitis in red-legged partridges shows neurotropism across avian species
Source: Nat Commun. 2026 Jun 5;17:7200. doi: 10.1038/s41467-026-73858-8 (PMC13396197; doi:10.1038/s41467-026-73858-8)
Supplement: Supplementary file 7 — Reporting Summary [file 41467_2026_73858_MOESM7_ESM.pdf]

nature portfolio

Corresponding author(s): Miguel Matos

Last updated by author(s): Apr 1, 2026

Reporting Summary

Nature Portfolio wishes to improve the reproducibility of the work that we publish. This form provides structure for consistency and transparency in reporting. For further information on Nature Portfolio policies, see our Editorial Policies and the Editorial Policy Checklist.

Statistics

For all statistical analyses, confirm that the following items are present in the figure legend, table legend, main text, or Methods section.

n/a	Confirmed	
☐	☒	The exact sample size (*n*) for each experimental group/condition, given as a discrete number and unit of measurement
☐	☒	A statement on whether measurements were taken from distinct samples or whether the same sample was measured repeatedly
☐	☒	The statistical test(s) used AND whether they are one- or two-sided<br>*Only common tests should be described solely by name; describe more complex techniques in the Methods section.*
☐	☐	A description of all covariates tested
☐	☒	A description of any assumptions or corrections, such as tests of normality and adjustment for multiple comparisons
☐	☒	A full description of the statistical parameters including central tendency (e.g. means) or other basic estimates (e.g. regression coefficient) AND variation (e.g. standard deviation) or associated estimates of uncertainty (e.g. confidence intervals)
☐	☒	For null hypothesis testing, the test statistic (e.g. *F*, *t*, *r*) with confidence intervals, effect sizes, degrees of freedom and *P* value noted<br>*Give P values as exact values whenever suitable.*
☒	☐	For Bayesian analysis, information on the choice of priors and Markov chain Monte Carlo settings
☒	☐	For hierarchical and complex designs, identification of the appropriate level for tests and full reporting of outcomes
☒	☐	Estimates of effect sizes (e.g. Cohen's *d*, Pearson's *r*), indicating how they were calculated

Our web collection on statistics for biologists contains articles on many of the points above.

Software and code

Policy information about availability of computer code

Data collection	No custom or commercial software was used for data collection. Clinical observations were recorded manually. Molecular data (e.g., qPCR Ct values) were exported using Agilent AriaMx software version 1.7
Data analysis	Data analysis was performed using Python version 3.13. The following open-source libraries were used: pandas (data manipulation), scipy (statistical testing), matplotlib and seaborn (data visualization). Serological data was assessed with Table Analyzer (Version 388 v1.00; GitHub repository: https://github.com/nicola-palmieri/TableAnalyzer; Accessed 389 2025.12.12)<br>For sequence analysis and genome assembly, CLC Genomics Workbench versions 23 and 24 (Qiagen Digital Insights) were used. Phylogenetic analyses were conducted using MegAlign Pro (DNASTAR Lasergene v18.0.1) and RAxML (Maximum Likelihood method).

For manuscripts utilizing custom algorithms or software that are central to the research but not yet described in published literature, software must be made available to editors and reviewers. We strongly encourage code deposition in a community repository (e.g. GitHub). See the Nature Portfolio guidelines for submitting code & software for further information.

Data

Policy information about availability of data

All manuscripts must include a data availability statement. This statement should provide the following information, where applicable:

- Accession codes, unique identifiers, or web links for publicly available datasets
- A description of any restrictions on data availability
- For clinical datasets or third party data, please ensure that the statement adheres to our policy

The complete genome sequences of ParPgV-A and ParPgV-C have been deposited in the NCBI GenBank under accession numbers PV472371 and PV472372, respectively. The raw sequencing data from all field samples used as inocula were submitted into the NCBI SRA database, under the BioProject PRJNA1314175. Data under accessions SAMN50922524 and SAMN50922527 corresponds to the inoculum used for animal experiment in grey partridges, whereas data with accessions SAMN50922526 and SAMN50922529 is from the inoculum used in animal experiments performed in red-legged partridges and SPF chickens.

All other data supporting the findings of this study, including viral load measurements, histopathology evaluations, serological data, statistical source data, primers, probes, cycling conditions, and amino acid identity matrix, are available within the manuscript and its supplementary files.

Research involving human participants, their data, or biological material

Policy information about studies with human participants or human data. See also policy information about sex, gender (identity/presentation), and sexual orientation and race, ethnicity and racism.

Reporting on sex and gender	Not applicable.
Reporting on race, ethnicity, or other socially relevant groupings	Not applicable.
Population characteristics	Not applicable.
Recruitment	Not applicable.
Ethics oversight	Not applicable.

Note that full information on the approval of the study protocol must also be provided in the manuscript.

Field-specific reporting

Please select the one below that is the best fit for your research. If you are not sure, read the appropriate sections before making your selection.

[x] Life sciences [ ] Behavioural & social sciences [ ] Ecological, evolutionary & environmental sciences

For a reference copy of the document with all sections, see nature.com/documents/nr-reporting-summary-flat.pdf

Life sciences study design

All studies must disclose on these points even when the disclosure is negative.

Sample size	Sample sizes were selected based on prior experience with similar avian virology experiments, considering ethical limitations and feasibility, while ensuring sufficient biological replicates for statistical analysis and reproducibility.
Data exclusions	No data were excluded from the analyses.
Replication	Experimental infections were performed in three independent animal groups (grey partridges, red-legged partridges, and SPF chickens), with multiple birds sampled at sequential time points. Results were consistent across animals within each group.
Randomization	Animals were randomly assigned to control or experimental groups. No further stratification was applied.
Blinding	Blinding was applied during histopathological scoring and MRI image interpretation. Investigators performing sample collection were not blinded, due to logistical constraints during necropsy and sampling.

Reporting for specific materials, systems and methods

We require information from authors about some types of materials, experimental systems and methods used in many studies. Here, indicate whether each material, system or method listed is relevant to your study. If you are not sure if a list item applies to your research, read the appropriate section before selecting a response.

Materials & experimental systems

n/a	Involved in the study
☐	☒ Antibodies
☒	☐ Eukaryotic cell lines
☒	☐ Palaeontology and archaeology
☐	☒ Animals and other organisms
☒	☐ Clinical data
☒	☐ Dual use research of concern
☒	☐ Plants

Methods

n/a	Involved in the study
☒	☐ ChIP-seq
☒	☐ Flow cytometry
☐	☒ MRI-based neuroimaging

Antibodies

Antibodies used

A custom rabbit polyclonal antibody was generated against the ParPgV-A envelope glycoprotein E2 (amino acids 414–570). The recombinant protein was expressed using the Bac-to-Bac Baculovirus system (Invitrogen) and purified via His-tag affinity chromatography (His GraviTrap™ TALON®, Cytiva, Marlborough, MA, USA). Polyclonal antibody production was outsourced to Davids Biotechnologie GmbH (Regensburg, Germany).
Primary antibody used: Rabbit anti-ParPgV E2 polyclonal serum (custom-made; no catalog number; dilution 1:500 for immunohistochemistry).
Secondary antibody: Biotinylated anti-rabbit IgG (Vector Laboratories, Burlingame, USA); used at 1:400 dilution (Immunohistochemistry); Goat anti-Galliformes IgY conjugated to horseradish peroxidase (IDvet, Grabels, France) used at 1:10000 (ELISA).

Validation

The antibody was validated in-house using formalin-fixed paraffin-embedded (FFPE) tissue from field-infected red-legged partridges and experimental animals. Specificity was confirmed by negative staining in tissues from non-inoculated SPF chickens and red-legged partridges, as well as birds infected with unrelated pathogens (e.g., fowl adenovirus, Histomonas meleagridis). Additional specificity testing of the rabbit polyclonal antibody was evaluated using a commercial multi-species competitive ELISA targeting conserved Orthoflavivirus E protein epitopes, confirming the absence of detectable cross-reactivity.
Signal specificity and background in immunohistochemistry were optimized using three different dilutions (1:100, 1:500, 1:1000), and 1:500 was selected based on optimal staining intensity and minimal background. Validation data are presented in the manuscript (Fig. 8 and 9, Supplementary Fig. 2).

Eukaryotic cell lines

Policy information about cell lines and Sex and Gender in Research

Cell line source(s): Not applicable.

Authentication: Not applicable.

Mycoplasma contamination: Not applicable.

Commonly misidentified lines (See ICLAC register): Not applicable.

Palaeontology and Archaeology

Specimen provenance: Not applicable.

Specimen deposition: Not applicable.

Dating methods: Not applicable.

☐ Tick this box to confirm that the raw and calibrated dates are available in the paper or in Supplementary Information.

Ethics oversight: Not applicable.

Note that full information on the approval of the study protocol must also be provided in the manuscript.

Animals and other research organisms

Policy information about studies involving animals; ARRIVE guidelines recommended for reporting animal research, and Sex and Gender in Research

Laboratory animals

This study involved three species of birds housed under controlled laboratory conditions:
- Red-legged partridges (Alectoris rufa), 5-month-old, obtained as embryonated eggs from a commercial supplier (Gibovendeé, France).

	- Grey partridges (Perdix perdix), 5-month-old, obtained from a local Austrian supplier.<br>- Specific-pathogen-free (SPF) White Leghorn chickens (Gallus gallus), 4-week-old, obtained as embryonated eggs from VALO BioMedia GmbH (Osterholz-Scharmbeck, Germany).
Wild animals	The study did not involve wild animals. All birds were sourced commercially and raised under controlled conditions.
Reporting on sex	Sex was not a primary experimental variable and birds were not sexed prior to inoculation due to the practical difficulty of sexing juvenile birds and the limited number of individuals per group and time point. However, sexing was routinely performed during necropsy as part of the post-mortem protocol, and this information was recorded for all animals.<br>Post hoc segregation of data by sex revealed no differences in viral load or pathological outcomes, although this may reflect the low sample size per sex at individual time points. Consequently, no sex-based analyses were performed in the main manuscript.
Field-collected samples	Field samples were obtained from three breeder flocks of red-legged partridges (Alectoris rufa) on two commercial farms in France experiencing outbreaks of neurological disease. Affected birds were culled by farm personnel based on clinical signs and submitted for diagnostic investigation. Post-mortem brain and organ tissues were collected and shipped to the University of Veterinary Medicine Vienna, Austria, for virological and histopathological analysis. These field-derived samples were used exclusively for diagnostic evaluation, histology, RNAscope, and genome characterization; they were not used for experimental inoculations. Additionally, no live animals were transported from field sites.
Ethics oversight	All animal procedures were reviewed and approved by the Ethics and Animal Welfare Committee of the University of Veterinary Medicine Vienna, Austria, and authorized by the Austrian Federal Ministry of Education, Science, and Research (approval numbers: BMBWF 2022-0.713.294 and Extension 2023-0.430.929).

Note that full information on the approval of the study protocol must also be provided in the manuscript.

Clinical data

Policy information about clinical studies
All manuscripts should comply with the ICMJE guidelines for publication of clinical research and a completed CONSORT checklist must be included with all submissions.

Clinical trial registration	Not applicable.
Study protocol	Not applicable.
Data collection	Not applicable.
Outcomes	Not applicable.

Dual use research of concern

Policy information about dual use research of concern

Hazards

Could the accidental, deliberate or reckless misuse of agents or technologies generated in the work, or the application of information presented in the manuscript, pose a threat to:

No	Yes	
☒	☐	Public health
☒	☐	National security
☒	☐	Crops and/or livestock
☒	☐	Ecosystems
☒	☐	Any other significant area

Experiments of concern

Does the work involve any of these experiments of concern:

No	Yes	
☒	☐	Demonstrate how to render a vaccine ineffective
☒	☐	Confer resistance to therapeutically useful antibiotics or antiviral agents
☒	☐	Enhance the virulence of a pathogen or render a nonpathogen virulent
☒	☐	Increase transmissibility of a pathogen
☒	☐	Alter the host range of a pathogen
☒	☐	Enable evasion of diagnostic/detection modalities
☒	☐	Enable the weaponization of a biological agent or toxin
☒	☐	Any other potentially harmful combination of experiments and agents

Plants

Seed stocks	Not applicable.
Novel plant genotypes	Not applicable.
Authentication	Not applicable.

ChIP-seq

Data deposition

☐ Confirm that both raw and final processed data have been deposited in a public database such as GEO.

☐ Confirm that you have deposited or provided access to graph files (e.g. BED files) for the called peaks.

Data access links<br>*May remain private before publication.*	Not applicable.
Files in database submission	Not applicable.
Genome browser session<br>(e.g. UCSC)	Not applicable.

Methodology

Replicates	Not applicable.
Sequencing depth	Not applicable.
Antibodies	Not applicable.
Peak calling parameters	Not applicable.
Data quality	Not applicable.
Software	Not applicable.

Flow Cytometry

Plots

Confirm that:

☐ The axis labels state the marker and fluorochrome used (e.g. CD4-FITC).

☐ The axis scales are clearly visible. Include numbers along axes only for bottom left plot of group (a 'group' is an analysis of identical markers).

☐ All plots are contour plots with outliers or pseudocolor plots.

☐ A numerical value for number of cells or percentage (with statistics) is provided.

Methodology

Sample preparation	Not applicable.
Instrument	Not applicable.
Software	Not applicable.
Cell population abundance	Not applicable.
Gating strategy	Not applicable.

☐ Tick this box to confirm that a figure exemplifying the gating strategy is provided in the Supplementary Information.

Magnetic resonance imaging

Experimental design

Design type	This study utilized a task-free, resting-state, longitudinal design with repeated MRI acquisitions at predefined time points (7, 10, 14, and 21 days post-infection). No task or stimulus was applied during MRI acquisition. Birds were under deep sedation, and imaging was conducted in a resting, non-responsive condition. The study did not use an event-related or block design.
Design specifications	Each bird underwent one MRI session per time point, with imaging performed at four defined time points: 7, 10, 14, and 21 days post-infection. The birds were under deep sedation during each session to minimize movement and stress. As this was a structural MRI study, not a task-based or functional MRI study, there were no experimental “blocks” or “trials” in the conventional sense.
Behavioral performance measures	Not applicable.

Acquisition

Imaging type(s)	Structural MRI study
Field strength	1.5 Tesla
Sequence & imaging parameters	T2-weighted CISS 3DT1-weighted TSE transverse T2-weighted TIR transverse T1-weighted GRE turbo flash 3D
Area of acquisition	Entire brain of the birds.

Diffusion MRI ☐ Used ☒ Not used

Preprocessing

Preprocessing software	Not applicable.
Normalization	Not applicable.
Normalization template	Not applicable.
Noise and artifact removal	Not applicable.
Volume censoring	Not applicable.

Statistical modeling & inference

Model type and settings	Not applicable. The evaluation of the MRI images was purely descriptive and based on qualitative assessment. No statistical modeling or inferential analysis was performed in this study.
Effect(s) tested	Not applicable. The evaluation of the MRI images was purely descriptive and based on qualitative assessment. No statistical

modeling or inferential analysis was performed in this study.

Specify type of analysis: ☒ Whole brain ☐ ROI-based ☐ Both

Statistic type for inference (See Eklund et al. 2016)	Not applicable. The evaluation of the MRI images was purely descriptive and based on qualitative assessment. No statistical modeling or inferential analysis was performed in this study.
Correction	Not applicable. The evaluation of the MRI images was purely descriptive and based on qualitative assessment. No statistical modeling or inferential analysis was performed in this study.

Models & analysis

n/a	Involved in the study
☒	☐ Functional and/or effective connectivity
☒	☐ Graph analysis
☒	☐ Multivariate modeling or predictive analysis
