# Supplementary material for: A pegivirus associated with encephalitis in red-legged partridges shows neurotropism across avian species
Source: Nat Commun. 2026 Jun 5;17:7200. doi: 10.1038/s41467-026-73858-8 (PMC13396197; doi:10.1038/s41467-026-73858-8)
Supplement: Supplementary file 8 — Transparent Peer Review file [file 41467_2026_73858_MOESM8_ESM.pdf]

nature portfolio

Peer Review File

Encephalitis Associated with a Neurotropic Pegivirus Supported by Experimental Infection Across Avian Species

Corresponding Author: Dr Miguel Matos

This file contains all reviewer reports in order by version, followed by all author rebuttals in order by version.

Version 0:

Reviewer comments:

Reviewer #1

(Remarks to the Author)

The authors present the results of an in-depth investigation of neurological disease in red-legged partridges that resulted in the discovery of novel avian pegiviruses. The authors further set up a series of experiments to determine whether the viruses caused the observed pathology, and to better characterize the pathogenesis of infection. This study represents a substantial amount of work and covers many of the steps one would want to see to show an association, if not causation, between virus and disease. Overall, this is an impressive, multi-layered study that provides several lines of evidence that a newly discovered partridge pegivirus is able to infect partridges and replicate in neurological tissue, causing pathology and observable clinical signs.

In general, the paper could be improved by adding some more detail about the outbreak and the scope of the study itself (see specific comments) as well as some re-organization of the presentation of information, based on the chronological order of the studies, to make the paper a bit easier to follow. It is unfortunate that the experimental findings are hard to interpret given the fact that naive individual red-legged partridges were not used, however, there is still sufficient evidence to show that pegivirus infection is associated with encephalitis in these birds and that the virus is able to replicate in neurologic and other tissues.

The following are specific comments and questions meant to be constructive and help clarify points made in the manuscript or to suggest where additional data or detail may strengthen the paper.

Line: comment

Title: Consider changing “of a” to “associated with”, since causation was not fully established for the encephalitis

29-30: The abstract should include details about the timing of the outbreak.

77: It might help the reader to establish up front the size, location and timing of the outbreaks that were investigated. How many farms/flocks / what was the species of partridge and how many individuals were showing signs in the initial cluster? This will help frame the overall epidemiological part of the study.

93: Were flocks A,B,and C on the same farm?

88: I think it would help the reader if a summary statement was made either at the end of the intro or beginning of methods that outlines all of the studies that were conducted as part of the investigation (e.g. epi, NGS, experimental, pathology, etc…), which will then make it easier to follow which techniques were applied during which part of the study. The methods are quite extensive but it isn’t always clear which technique was used when.

265-7: What was the purpose of the sampling activity? How were the farms selected and where are the farms relative to the two outbreak farms mentioned at the beginning of the paper? It should be included at the end of the intro that the investigation was set up across eight farms. Otherwise, it seems like the study was limited to the two farms. How were the 6 control farms selected? Was the stock from each unconnected, or was there trade among these farms? As you found out,

farms not reporting encephalitis were not negative for the pathogen. Would you propose future studies to better understand the distribution and prevalence of pegivirus on farms in France? (for the discussion).

How was the sample size of 30 birds (15M 15F) determined?

287-88: What were the relative detection rates or viral loads of strain A and C in the field studies?

A note about organization - it seems the authors are reporting a result here - that there were two strains in the inoculum and that strain C was most prevalent following the grey partridge experimental infection study, and then this finding informs the methodology of the subsequent experimental infections. Then, the experimental study results appear in the next section and it's unclear whether the first experiment in included in this section, or not. I suggest finding a more chronologically consistent way to discuss the results of the experimental infections.

309: "euthanized" is a more appropriate term than "culled," here.

361-64: Are the authors reporting results from the study in describing events prior to 2021? This seems like background detail rather than study results.
Also, the speculation about the impact of culling on transmission belongs in the discussion. There is no data related to how the virus is transmitted, nor whether asymptomatic transmission occurs in which case culling would not impact transmission.

368-69 - were the clinical signs observed more in female birds?

371-72 - could the authors clarify what impact they're referring to? The impact of culling?

373: How was the 2% prevalence estimate calculated? Was this on one farm, or both farms, or an average? It would help to show numbers infected over total population. Also noting that this would be an underestimate if made only on clinical signs rather than lab confirmed diagnosis (for discussion).

More detail about the structure of the flocks would also be informative. For example, were males paired with females in each cage, or were males separated from females? If there was separation, and an outbreak occurred in females during laying periods, that could explain both a higher detection frequency and viral loads in females compared to males.

422: Were multiple strains found in a single bird, or a pooled sample?

463: Since the epidemiological investigation is a significantpart of the paper, more data should be provided regarding how many birds were sampled from each flock and farm, and how many were infected of those sampled, along with prevalence estimated and confidence intervals.
These data could be presented in a single table.

466-47: The authors should include data and statistics to support the statement that female samples were more frequently positive than males. The data presented in the figure is viral load data, but not detection frequency data. The authors should clarify that on average, females had higher viral load than males, but as far as detection rates, the sample sizes were quite small, and a chi sq. or Fisher's exact test would determine whether there was a significant difference in the proportion of males and females infected.

528-9: Which previous investigations?

568: What was the rationale for infecting chickens?

689-690: It would seem worth noting that the viruses found do not seem to replicate well in grey partridges or chickens, supporting the idea of narrow host plasticity.

708 - to help the reader, I suggest specifying which bird species is being discussed.

757-758: also West Nile virus (American robins vs corvids)

760-761: Alternatively, poor receptor binding affinity of the virus to chicken cell receptors vs red-legged partridge

Did the partridge farms also have commercial poultry flocks? What would the risk be to chickens on farms with red partridges?

763: I suggest elaborating on the risk to wildlife - this is an important point: Is there viral exchange occurring between wild partridge populations and farmed ones? Could wild birds spread this pegivirus among farms? What is the impact on wild bird health?

Reviewer #2

(Remarks to the Author)
In their manuscript “Neurotropism and Encephalitis of a Novel Pegivirus with Experimental Evidence Across Avian Species,” Matos, Bilic et al. report the discovery and in vivo characterization of a novel avian pegivirus (Partridge pegivirus, ParPgV) associated with encephalitic outbreaks in red-legged partridges in France. While pegivirus-like sequences have been increasingly documented in global metagenomic surveys of wild birds, this study provides the first robust experimental evidence linking a pegivirus to neuropathology. The authors combine metagenomics, histopathology, electron microscopy, MRI, and controlled infection studies across three avian hosts to establish ParPgV as a neurotropic virus capable of inducing cerebellar atrophy and histological encephalitis. This foundational work elevates pegiviruses from incidental findings in sequencing datasets to bona fide avian pathogens and represents a significant advance in our understanding of Flaviviridae host range and pathogenicity.

While the paper makes a foundational contribution by demonstrating pegivirus replication in neural tissues and potential for encephalitic lesions, its central claim, that ParPgV causes neurologic disease, remains only partially substantiated. The lack of naïve hosts, ambiguous clinical phenotype, and mild pathology limit the strength of causal inference. However, the work lays important groundwork and appropriately refrains from overstating its findings in most places.

Major comments
A key limitation of the grey partridge model is the use of a mixed inoculum containing both ParPgV-A and ParPgV-C, identified only retrospectively. The relative abundance of each strain was not determined, and no strain-specific qPCR or remapping analysis was performed, making it impossible to link viral loads or pathology to a specific genotype. This confounds the interpretation of the grey partridge data in Figure 4. While the red-legged partridge and SPF chicken experiments are described as using only ParPgV-C, the manuscript provides no sequencing or RT-PCR based validation to confirm the absence of ParPgV-A.

The in vivo experiments are limited by small sample sizes, incomplete baseline screening, and a lack of mock-infected controls, undermining the strength of causal inference. In red-legged partridges, pre-existing infection (with unknown strain) at baseline confounds attribution of pathology to experimental infection. The reported dip in viremia at 10 dpi followed by a rise at 14 dpi in Figure 4c may suggest biphasic kinetics, but given the low n and variability, stochastic fluctuation cannot be ruled out. Figure 5a further illustrates these limitations: the histological panels lack animal identifiers, viral load data, and inoculum information, making it difficult to contextualize the findings. Moreover, no uninfected control tissues are shown for comparison, and the observed lesions, congestion and lymphoid infiltration, are, at least to my eyes, mild. As presented, the pathology does not compellingly demonstrate virus-specific effects and leaves the overall infection model underpowered to support strong conclusions.

Minor comments
The severity of neuropathology may be overstated. While the figure legend for Fig. 5d describes “severe inflammatory infiltrates” and “neuronal degeneration and necrosis,” the presented cerebellar histology appears relatively mild, with only modest infiltrates and no overt evidence of widespread neuronal loss or Purkinje cell degeneration. Without quantitative scoring, additional stains, or side-by-side mock controls, these interpretations seem somewhat speculative. Although I am not a trained pathologist, the images do not strongly support the claim of severe neuroinflammation or neurodegeneration as written.

Line 436 and elsewhere, Flaviviridae should be italicised.

Figure 2: The depiction of the 5′ and 3′ UTRs as rounded rectangles extending beyond the genome creates the misleading impression that these regions extend past the actual UTR boundaries. Both ParPgV-A and ParPgV-C should be shown with properly annotated UTRs, as done in Supplementary Figure 2, for consistency and accuracy. Furthermore, the figure currently only marks the signalase cleavage site; no putative polyprotein cleavage sites are annotated, and the “gene boundaries” appear to represent protein domains rather than predicted mature proteins. Inclusion of predicted cleavage sites and clarification of what the boundaries represent would strengthen the figure. Finally, the overall nucleotide identity between ParPgV-A and ParPgV-C should be reported in the main text to contextualize their relationship.

The manuscript does not clarify whether the red-legged partridge in Video S1 was confirmed to be ParPgV-positive; without direct testing, it should be explicitly described as anecdotal and not used as supporting evidence. Video S2 is unconvincing; multiple uncontrolled factors influence activity levels, and the footage lacks side-by-side cage status, standardized conditions or even standard video composition. Removal of Video S2 is recommended. Also there is a difference in enrichment between the control and inoculated cages in S2.

Supplementary Figure 2b is unclear regarding the 3′ end of the ParPgV-C genome—please clarify whether this represents an extended 3′ UTR or an unannotated coding region, it looks like a white box is over the 3'UTR and cutting off the letters from UTR.

The legend of Supplementary Figure 2 states that ParPgV-C has longer 5′ and 3′ UTRs than ParPgV-A, yet the general and strain-specific PCR primer positions are depicted identically between the two genomes, please clarify how primer binding sites can align if UTR lengths and genome coordinates differ. Additionally, Supplementary Table 1 does not indicate which primers are "variant-specific" or how they correspond to the labelled PCRs in Supplementary Figure 2; please clarify which primer pairs were used for each screening assay and ensure coordinate consistency across the figure and table.

Where the infecting strain is known, it should be indicated in the relevant figure legends to aid interpretation. For example, Fig. 5 could be revised to read: "Fig. 5. Viral load dynamics, histopathological lesions, and MRI findings in red-legged partridges (Alectoris rufa) experimentally inoculated with ParPgV-C."

Figures 4c and 5a both present viral gRNA quantitation but differ in visual representation and interpretation. While Figure 5a and its legend describe a peak at 14 dpi, the violin plots show narrower distributions and potentially lower variability at that time point than at 7 or 10 dpi. In contrast, the heatmap in Figure 4c suggests viremia peaks earlier, particularly at 7 dpi. To reconcile these differences, the authors should consider plotting individual data points or using consistent visual formats (e.g. line plots) across figures to more accurately reflect temporal dynamics. It is also unclear what threshold defines a negative result (e.g. Ct > 38 or <1 copy/reaction), and whether this was applied consistently—several samples appear negative in Figure 4c, yet all blood samples in Figure 5a are plotted as positive, including those with <1 genome copy per reaction. This should be clarified.

The phylogenetic analysis lacks key methodological details, including the GBlocks trimming parameters and the specific protein substitution model used in RAxML, while likely appropriate, must be explicitly reported. Full version information for all software tools (MUSCLE, GBlocks, RAxML, MegAlign Pro) should also be included.

The qPCR field data in Figure 3 are difficult to interpret due to the use of ΔCt (38 – Ct) without reporting actual Ct values, viral copy numbers, or a clearly defined limit of detection (LOD). As a result, many samples appear "positive" despite likely being near or below the assay's detection threshold. This issue is particularly problematic given the presence of two distinct ParPgV strains, yet no attempt is made to differentiate or quantify them separately in these field samples. The authors should report the Ct threshold for positivity directly on the figure, clarify whether any pools contained negative samples, and ideally provide the proportion of positive pools per farm and sample type. Inclusion of raw Ct values or viral copy numbers, alongside explicit designation of the LOD and clarification of strain composition, would substantially improve interoperability.

The authors should deposit the raw sequencing data from the inoculum and sample datasets on the NCBI SRA database. Additionally, sequencing of the other inocula used in the red-legged partridge and SPF chicken experiments should be performed to confirm the absence of mixed infections.

For both the IHC/RNAscope brain staining (Fig. 7) and the cerebellar MRI images (Fig. 8), it is unclear at what time point post-infection the samples were taken.

The use of recombinant E2 protein to generate the polyclonal antibody for IHC is appropriate, but the inclusion of serological testing would strengthen the study. Serology could clarify baseline infection status and provide independent evidence of immune recognition. Moreover, testing infected partridge serum against the recombinant E2 protein would help validate antibody specificity.

The manuscript does not define the Ct threshold used to classify samples as positive, negative, or inconclusive for negative-strand RNA, nor does it describe how these classifications were validated. Without this information, the designations in Supplementary Table 3 are difficult to interpret and may overstate evidence of active replication. The authors should clearly state the Ct cutoffs, include appropriate controls, and clarify how inconclusive results were determined.

The manuscript does not specify which strain the antigen was derived from, whether it cross-reacts with both genotypes, or the E2 amino acid identity between strains, limiting interpretation of the IHC results and broader applicability of the antibody.

While the histopathological findings are convincing and supported by viral load data, the MRI results are difficult to interpret. With only two infected and one control bird imaged per time point, and no quantification of cerebellar volume or signal intensity, the evidence for atrophy remains anecdotal. The authors should clarify whether the phenotype was consistent across both infected individuals and distinguishable from natural variation. Including all MRI scans as a supplementary figure and attempting even limited quantification, despite the small sample size, would help strengthen this aspect of the study.

Reviewer #3

(Remarks to the Author)
The manuscript Neurotropism and Encephalitis of a Novel Pegivirus with Experimental Evidence Across Avian Species by Matos et al., describes a novel avian pegivirus (partridge pegivirus, ParPgV) associated with field outbreaks of encephalitis in red-legged partridges (Alectoris rufa) in France. The authors have employed multiple methods to identify the new

pegivirus (such as, next-generation sequencing, full-length genomic characterization), and its associated neurotropism (such as, histopathology and electron microscopy), and further performed confirmatory experimental infections in multiple avian species.
I found the research plan expertly executed, and the manuscript flawless. The outcome is original and significant for the field. I do not have further remarks for the authors. I recommend the manuscript for publication, and I compliment the authors for their robust contribution to the scientific field.

Version 1:

Reviewer comments:

Reviewer #2

(Remarks to the Author)
The authors have addressed the majority of my comments satisfactorily, and the revised manuscript is substantially improved as a result. In particular, the additional clarification of inoculum composition, the inclusion of strain-specific qPCR data, and the improved presentation of methodological and experimental details have considerably strengthened the study.

Some limitations in the study design remain, particularly regarding host background infection status and the interpretability of some in vivo findings, and these should continue to be acknowledged clearly in the manuscript. However, I do not consider these remaining issues sufficient to preclude publication in revised form.

Reviewer #4

(Remarks to the Author)
The authors have largely addressed the concerns of reviewer 1 by revising the language around epidemiological findings. However, I agree that the authors have not sufficiently recapitulated clinical presentation of disease observed at partridge farms in their in vivo (but not naive) model to directly link encephalitis to infection with ParPgV as the MRI shows volume loss rather than swelling of the brain tissue. I agree that the title should be revised from "of a" to "associated with".

Open Access This Peer Review File is licensed under a Creative Commons Attribution 4.0 International License, which permits use, sharing, adaptation, distribution and reproduction in any medium or format, as long as you give appropriate credit to the original author(s) and the source, provide a link to the Creative Commons license, and indicate if changes were made.
In cases where reviewers are anonymous, credit should be given to 'Anonymous Referee' and the source.
The images or other third party material in this Peer Review File are included in the article's Creative Commons license, unless indicated otherwise in a credit line to the material. If material is not included in the article's Creative Commons license and your intended use is not permitted by statutory regulation or exceeds the permitted use, you will need to obtain permission directly from the copyright holder.
To view a copy of this license, visit https://creativecommons.org/licenses/by/4.0/

We thank the reviewers for their careful evaluation of our manuscript and for the constructive and insightful comments provided. We have addressed all points raised in detail below and have revised the manuscript accordingly. Reviewer comments are reproduced in full and are followed by our point-by-point responses, with corresponding changes clearly indicated in the revised manuscript.

Reviewer comments

Reviewer #1 (R1) (Remarks to the Author):

"*The authors present the results of an in-depth investigation of neurological disease in red-legged partridges that resulted in the discovery of novel avian pegiviruses. The authors further set up a series of experiments to determine whether the viruses caused the observed pathology, and to better characterize the pathogenesis of infection. This study represents a substantial amount of work and covers many of the steps one would want to see to show an association, if not causation, between virus and disease. Overall, this is an impressive, multi-layered study that provides several lines of evidence that a newly discovered partridge pegivirus is able to infect partridges and replicate in neurological tissue, causing pathology and observable clinical signs.*

In general, the paper could be improved by adding some more detail about the outbreak and the scope of the study itself (see specific comments) as well as some re-organization of the presentation of information, based on the chronological order of the studies, to make the paper a bit easier to follow. It is unfortunate that the experimental findings are hard to interpret given the fact that naive individual red-legged partridges were not used, however, there is still sufficient evidence to show that pegivirus infection is associated with encephalitis in these birds and that the virus is able to replicate in neurologic and other tissues.

The following are specific comments and questions meant to be constructive and help clarify points made in the manuscript or to suggest where additional data or detail may strengthen the paper."

"Line: Comment"

R1: *Title: Consider changing "of a" to "associated with", since causation was not fully established for the encephalitis*

A: We thank the reviewer for the thoughtful comment. We agree that establishing definitive causation in viral encephalitis can be complex. However, our study provides strong experimental evidence supporting an etiological role of the novel pegivirus, including detection of negative-strand RNA replication in neural tissues, histopathological and MRI evidence of encephalitis in experimentally infected birds, and localization of viral RNA and antigen in affected neurons. For this reason, we believe the current study and the applied methods accurately reflects the scope and strength of our findings while the text carefully discusses the limitations regarding causality.

R1: *29-30: The abstract should include details about the timing of the outbreak.*

A: We thank the reviewer for this suggestion. The temporal context of the field outbreaks and the subsequent experimental studies is described in detail in the Materials and Methods (lines 92–101) and Results (lines 415–432). To maintain the abstract concise and focused on the principal findings, we preferred not to expand it with additional temporal details. We believe the current wording sufficiently frames the study without exceeding the abstract's scope and length.

R1: *77: It might help the reader to establish up front the size, location and timing of the outbreaks that were investigated. How many farms/flocks / what was the species of partridge and how many individuals were showing signs in the initial cluster? This will help frame the overall epidemiological part of the study.*

A: We thank the reviewer for the valuable suggestion. The epidemiological details of the outbreaks, including the number of affected farms, flock sizes, timing, and proportion of birds showing clinical signs, are described in detail in the Materials and Methods (lines 92–101) and Results (lines 415–432). We believe that the Introduction is best focused on outlining the scientific context and rationale for the study, namely, the discovery of a novel pegivirus linked to encephalitic lesions, while specific outbreak information is more appropriately presented in the later sections, especially the first paragraph of Materials and Methods

R1: *93: Were flocks A, B, and C on the same farm?*

A: We thank the reviewer for the question and the opportunity to clarify this point. As indicated in line 94, the outbreaks occurred on two geographically proximate farms in France. Flocks A, B, and C were located on these two farms and were managed as independent epidemiological units, each representing a distinct breeder flock. We have now added a brief clarification in the text to avoid any ambiguity (line 95-97).

R1: *88: I think it would help the reader if a summary statement was made either at the end of the intro or beginning of methods that outlines all of the studies that were*

conducted as part of the investigation (e.g. epi, NGS, experimental, pathology, etc…), which will then make it easier to follow which techniques were applied during which part of the study. The methods are quite extensive but it isn't always clear which technique was used when.

A: We thank the reviewer for this helpful suggestion. While the Materials and Methods section provides detailed descriptions of each component of the study, we agree that a concise overview sentence at the end of the Introduction can guide the reader through the different approaches. We have therefore extended slightly the summary statement at the end of the Introduction (lines 84-87).

R1: *265-7: What was the purpose of the sampling activity? How were the farms selected and where are the farms relative to the two outbreak farms mentioned at the beginning of the paper? It should be included at the end of the intro that the investigation was set up across eight farms. Otherwise, it seems like the study was limited to the two farms. How were the 6 control farms selected? Was the stock from each unconnected, or was there trade among these farms? As you found out, farms not reporting encephalitis were not negative for the pathogen. Would you propose future studies to better understand the distribution and prevalence of pegivirus on farms in France? (for the discussion).*

A: We thank the reviewer for these important questions and the opportunity to clarify the rationale and scope of the field sampling.
The primary objectives of the field sampling were (1) to assess the presence and distribution of ParPgV across red-legged partridge farms and (2) to identify potential ParPgV-negative flocks that could serve as a source of embryonated eggs for experimental infection studies, as explained in line 896-898. The latter objective proved unsuccessful, as ParPgV was detected across all investigated farms.
Farms were not selected according to predefined epidemiological criteria. Instead, sampling included farms with a documented history of neurological signs consistent with encephalitis, as well as farms without reported clinical disease, in order to provide an initial field-based overview. The eight farms sampled included the two farms where encephalitis outbreaks had been documented and six additional farms with no reported neurological disease at the time of sampling. These farms were geographically distributed within the same production region.
Due to the nature of red-legged partridge production, which is substantially less centralized and standardized than commercial poultry systems, detailed information on animal movements, trade connections, or shared stock between farms was not available. Consequently, it was not possible to formally assess epidemiological links or independence between farms.
The finding that ParPgV was also detected in farms without reported encephalitis highlights that infection alone is insufficient to predict clinical disease and suggests that additional host, environmental, or management-related factors likely modulate

disease expression, as discussed in lines 907-912. We agree that future studies should aim to investigate the broader distribution, prevalence, and transmission dynamics of pegiviruses in partridge populations, as already mentioned in lines 960-962.

R1: *How was the sample size of 30 birds (15M 15F) determined?*

A: We thank the reviewer for this important question. The sample size of 30 birds per flock (15 males and 15 females) was chosen according to standard epidemiological sampling principles used in avian surveillance studies. The number of individuals required to detect at least one infected bird with a specified confidence depends on the true within-flock infection prevalence (p), the test sensitivity (Se), and the desired confidence level (C), calculated as $n = \ln(1 - C)/\ln(1 - p \times Se)$. Assuming a design prevalence of 10 % ($p = 0.10$), a 95 % confidence level ($C = 0.95$), and perfect test sensitivity ($Se = 1$), this gives $n = \ln(1 - 0.95)/\ln(1 - 0.10) = 28.4$, which was rounded up to 30. Information about this was added in lines 285-286.

R1: *287-88: What were the relative detection rates or viral loads of strain A and C in the field studies?*

A: This information is now available in Supplementary Table 3.

R1: *A note about organization - it seems the authors are reporting a result here - that there were two strains in the inoculum and that strain C was most prevalent following the grey partridge experimental infection study, and then this finding informs the methodology of the subsequent experimental infections. Then, the experimental study results appear in the next section and it's unclear whether the first experiment in included in this section, or not. I suggest finding a more chronologically consistent way to discuss the results of the experimental infections.*

A: We thank the reviewer for this helpful comment and understand that the reference to the "first in vivo experiment" may have caused uncertainty about the experimental sequence. The "first in vivo experiment" refers to the grey partridge trial, which was the initial study in the series. To clarify this chronology, we have revised the paragraph (lines 302 and 310-311) to specify the species involved and to indicate that the subsequent experiments in red-legged partridges and SPF chickens followed this initial trial in agreement with table 1.

R1: *309: "euthanized" is a more appropriate term than "culled," here.*

A: We thank the reviewer for the observation. We have replaced the term "culled" with "euthanized" to more accurately reflect humane termination procedures performed under ethical approval in the experimental setting.

R1: *361-64: Are the authors reporting results from the study in describing events prior to 2021? This seems like background detail rather than study results. Also, the speculation about the impact of culling on transmission belongs in the discussion. There is no data related to how the virus is transmitted, nor whether asymptomatic transmission occurs in which case culling would not impact transmission.*

A: We thank the reviewer for this comment. The section in question was intended to provide a chronological description of the field outbreaks that formed the basis of the study. The information referring to events prior to 2021 constitutes part of the observed outbreak history and clinical context of the affected flocks, rather than introductory background. For this reason, we consider its inclusion in the Results appropriate, as it documents field observations that informed subsequent investigations.
We acknowledge that the brief remark regarding culling as a potential factor influencing transmission was interpretative in nature and have therefore rephrased this sentence (line 419-420) to avoid speculative wording while retaining the factual description of management practices during the outbreaks.

R1: *368-69 - were the clinical signs observed more in female birds?*

A: We thank the reviewer for this question. Individual-level documentation of clinical signs by sex was not systematically recorded during the field outbreaks, and therefore we cannot provide quantitative sex-specific morbidity data. However, neurological signs were predominantly noted during the laying period, coinciding with a measurable reduction in egg production, suggesting that clinically apparent disease was more frequently observed in females.

R1: *371-72 - could the authors clarify what impact they're referring to? The impact of culling?*

A: We appreciate this comment and have clarified the sentence to specify that the observed impact on flock productivity resulted from culling (i.e., removal of actively laying breeders), not from reduced laying due to disease (lines 426-427).

R1: *373: How was the 2% prevalence estimate calculated? Was this on one farm, or both farms, or an average? It would help to show numbers infected over total population. Also noting that this would be an underestimate if made only on clinical signs rather than lab confirmed diagnosis (for discussion).*

A: We thank the reviewer for this comment and appreciate the opportunity to clarify. The 2% figure represents an approximate morbidity estimate based on clinical observations made by veterinary and farm personnel during the 2021 laying season, rather than a calculated infection rate. At the time of the outbreak, the causative agent

was still unknown, and laboratory confirmation was not yet possible. We have rephrased the sentence to make this clear in line 429.

R1: *More detail about the structure of the flocks would also be informative. For example, were males paired with females in each cage, or were males separated from females? If there was separation, and an outbreak occurred in females during laying periods, that could explain both a higher detection frequency and viral loads in females compared to males.*

A: We thank the reviewer for this comment. Each breeding pair consisted of one male and one female housed together within individual cages, which is the standard management practice in red-legged partridge breeder flocks. The description in lines 93–101 already refers to this configuration, but we have now clarified it explicitly to avoid any possible ambiguity.

R1: *422: Were multiple strains found in a single bird, or a pooled sample?*

A: We thank the reviewer for this question. The two ParPgV strains were identified in a single brain sample obtained from an individual bird, not from a pooled sample.

R1: *463: Since the epidemiological investigation is a significantpart of the paper, more data should be provided regarding how many birds were sampled from each flock and farm, and how many were infected of those sampled, along with prevalence estimated and confidence intervals.*
These data could be presented in a single table.

A: We thank the reviewer for this comment and appreciate the interest in the epidemiological component of the study. The field investigation was designed as a farm-level screening using pooled samples rather than an individual-level prevalence study. For each flock, 30 adult birds (15 males and 15 females) and 30 embryonated eggs were sampled, as described in detail in the Materials and Methods (lines 282–290). Samples were pooled in groups of five prior to qRT-PCR testing, precluding precise estimation of individual-level prevalence or confidence intervals.
The primary aim of this screening was to assess farm-level presence and distribution of ParPgV across different sample matrices and to identify potential ParPgV-negative flocks, rather than to calculate individual bird prevalence. To emphasize this we have now updated Figure 3 to convey more detailed information on the field screening results of field samples

R1: *466-47: The authors should include data and statistics to support the statement that female samples were more frequently positive than males. The data presented in the figure is viral load data, but not detection frequency data. The authors should clarify that on average, females had higher viral load than males, but as far as*

detection rates, the sample sizes were quite small, and a chi sq. or Fisher's exact test would determine whether there was a significant difference in the proportion of males and females infected.

A: We thank the reviewer for this important clarification. We agree that the field data primarily support differences in viral load rather than formal comparisons of detection frequency between sexes. Accordingly, the revised Figure 3 now presents viral load distributions using box plots with individual data points, allowing visual assessment of this pattern. Additionally, the raw data is available in Supplementary Table 3.
The field screening was designed as a farm-level, pooled-sample investigation to assess virus presence and relative viral burden across sample types, rather than to compare sex-specific prevalence. Given the pooled sampling strategy and limited number of pools per sex and farm, statistical testing of detection frequency would not be robust.

R1: *528-9: Which previous investigations?*

A: We thank the reviewer for the comment. The phrase "previous investigations" referred to the field surveillance results presented earlier in the manuscript (lines 526–535, Fig. 3), which documented ParPgV detection in multiple red-legged partridge flocks. To make this clearer, we have revised the sentence (602-603).

R1: *568: What was the rationale for infecting chickens?*

A: We thank the reviewer for this observation. The rationale for including SPF chickens in the study was to assess susceptibility in a heterologous host and to evaluate their suitability as a defined laboratory model, compared with partridges, which is discussed in lines 916 ff. To clarify this point earlier in the manuscript, we have added a brief explanation at the beginning of the SPF chicken experiment section (lines 683-684).

R1: *689-690: It would seem worth noting that the viruses found do not seem to replicate well in grey partridges or chickens, supporting the idea of narrow host plasticity.*

A: We thank the reviewer for this helpful observation. We agree that the limited viral replication and absence of overt neuropathology observed in grey partridges and SPF chickens are consistent with a restricted host permissiveness outside red-legged partridges. We have now added a brief statement to the Discussion highlighting this point (lines 944-947).

R1: *708 - to help the reader, I suggest specifying which bird species is being discussed.*

A: We thank the reviewer for this helpful suggestion. We have clarified the text to specify the bird species discussed at this point in the Discussion (lines 873-874).

R1: *757-758: also West Nile virus (American robins vs corvids)*

A: We thank the reviewer for this suggestion. An additional example referring to host-dependent differences in West Nile virus infection has now been included in the manuscript (line 948).

R1: *760-761: Alternatively, poor receptor binding affinity of the virus to chicken cell receptors vs red-legged partridge*

A: We thank the reviewer for this insightful suggestion. We have now added a brief statement to the Discussion, in lines 922-923.

R1: *Did the partridge farms also have commercial poultry flocks? What would the risk be to chickens on farms with red partridges?*

A: We thank the reviewer for this relevant question. Information on the presence of commercial poultry flocks on or near the investigated red-legged partridge farms was not systematically documented during the field investigations. Consequently, we cannot formally assess epidemiological links or transmission risk between partridge and commercial poultry operations based on the available data.
It should be noted, however, that husbandry conditions for red-legged partridges differ substantially from those of commercial chickens, including housing systems, stocking density, management practices, and biosecurity measures. These differences are likely to influence contact structure and transmission dynamics between species. In addition, experimental infection of SPF chickens demonstrated viral replication without clinical disease or neuropathological lesions, suggesting limited pathogenic impact in this host under controlled conditions.

R1: *763: I suggest elaborating on the risk to wildlife - this is an important point: Is there viral exchange occurring between wild partridge populations and farmed ones? Could wild birds spread this pegivirus among farms? What is the impact on wild bird health?*

A: We thank the reviewer for raising this important point. We agree that the potential involvement of wild bird populations in the ecology and transmission of ParPgV is a highly relevant question. However, the present study was limited to farmed red-legged partridges and experimentally inoculated birds, and no sampling of wild partridge populations or other free-ranging avian species was performed. Consequently, we cannot assess viral exchange between wild and farmed populations, the role of wild

birds in virus dissemination among farms, or the impact of ParPgV on wild bird health based on the current data.
However, we expanded the discussion in lines 960-962 to highlight the reviewers point.

Reviewer #2 (R2) (Remarks to the Author):

"*In their manuscript "Neurotropism and Encephalitis of a Novel Pegivirus with Experimental Evidence Across Avian Species," Matos, Bilic et al. report the discovery and in vivo characterization of a novel avian pegivirus (Partridge pegivirus, ParPgV) associated with encephalitic outbreaks in red-legged partridges in France. While pegivirus-like sequences have been increasingly documented in global metagenomic surveys of wild birds, this study provides the first robust experimental evidence linking a pegivirus to neuropathology. The authors combine metagenomics, histopathology, electron microscopy, MRI, and controlled infection studies across three avian hosts to establish ParPgV as a neurotropic virus capable of inducing cerebellar atrophy and histological encephalitis. This foundational work elevates pegiviruses from incidental findings in sequencing datasets to bona fide avian pathogens and represents a significant advance in our understanding of Flaviviridae host range and pathogenicity.*

While the paper makes a foundational contribution by demonstrating pegivirus replication in neural tissues and potential for encephalitic lesions, its central claim, that ParPgV causes neurologic disease, remains only partially substantiated. The lack of naïve hosts, ambiguous clinical phenotype, and mild pathology limit the strength of causal inference. However, the work lays important groundwork and appropriately refrains from overstating its findings in most places."

R2: Major comments

R2: *A key limitation of the grey partridge model is the use of a mixed inoculum containing both ParPgV-A and ParPgV-C, identified only retrospectively. The relative abundance of each strain was not determined, and no strain-specific qPCR or remapping analysis was performed, making it impossible to link viral loads or pathology to a specific genotype. This confounds the interpretation of the grey partridge data in Figure 4. While the red-legged partridge and SPF chicken experiments are described as using only ParPgV-C, the manuscript provides no sequencing or RT-PCR based validation to confirm the absence of ParPgV-A.*

A: We thank the reviewer for this thoughtful and detailed comment. We would like to clarify that ParPgV-A and ParPgV-C are not distinct genotypes but closely related strains with high nucleotide identity. Nonetheless, we agree that the retrospective

identification of both strains in the grey partridge inoculum represents a limitation for strain-specific interpretation of the GP experiment.

To directly address this concern, we have now performed additional strain-specific qRT-PCR analyses. First, strain-specific assays were applied to the original GP inoculum to determine the relative abundance of ParPgV-A and ParPgV-C (now presented in Supplementary Table 4a). Second, strain-specific qRT-PCR was performed on organ, blood, and swab samples collected from the grey partridge experiment to assess the presence and relative predominance of each strain during infection (Supplementary Table 4a-b). These data demonstrate that ParPgV-C predominated in the infected tissues.

Regarding the red-legged partridge and SPF chicken experiments, we now provide explicit validation that only ParPgV-C was present in the inocula used for these studies, based on strain-specific qRT-PCR analysis (Supplementary Table 4a). We have revised the manuscript accordingly to clearly document these additional controls and analyses (lines 302-314).

R2: *The in vivo experiments are limited by small sample sizes, incomplete baseline screening, and a lack of mock-infected controls, undermining the strength of causal inference.*

A: We thank the reviewer for this important and thoughtful assessment. We acknowledge that the in vivo experiments are constrained by sample size, baseline screening limitations, and the absence of mock-infected controls. These limitations largely reflect practical and ethical constraints associated with obtaining and experimentally infecting game bird species such as grey and red-legged partridges, which are not readily available laboratory models.

The grey partridge experiment was intentionally designed as a preliminary exploratory study to assess susceptibility and optimize subsequent experimental design. In contrast, the red-legged partridge (RLP) and SPF chicken experiments were conducted with larger group sizes (n = 20 per species), which we consider appropriate for controlled in vivo infection studies in avian species. In these latter experiments, baseline screening was performed, and we have now added ELISA-based serological data to provide complementary evidence of infection status and immune response over the course of the experiments.

Furthermore, to address concerns regarding strain composition and interpretation of mixed inoculum, we have performed additional strain-specific qRT-PCR analyses of the inocula and of tissues collected from the grey partridge experiment, demonstrating predominance of ParPgV-C (Supplementary Table 4; see above). Strain-specific qRT-PCR was also used to confirm the exclusive presence of ParPgV-C in the inocula used for the RLP and SPF chicken experiments (Supplementary Table 4a).

While we fully agree that these constraints limit definitive causal inference, we believe that the convergence of field epidemiology, viral detection and replication in neural tissues together with experimental infection outcomes across multiple avian species,

histopathology, and newly added serological data provides strong and coherent evidence supporting the pathogenic potential of ParPgV.

R2: *In red-legged partridges, pre-existing infection (with unknown strain) at baseline confounds attribution of pathology to experimental infection.*

A: We thank the reviewer for highlighting this important limitation. We agree that pre-existing ParPgV infection in red-legged partridges represents a confounding factor for attributing pathological findings exclusively to experimental inoculation, as we highlighted in lines 896-898 of the discussion.
Prior to initiating the in vivo studies, we conducted extensive field screening across eight red-legged partridge farms, including adult birds and embryonated eggs, with the aim of identifying ParPgV-negative source material. Unexpectedly, ParPgV RNA was detected in all investigated farms and sample types, precluding the acquisition of ParPgV-free red-legged partridges for experimental infection. This finding itself underscores the widespread distribution of the virus in farmed partridge populations and constrained the experimental design.
In this context, the red-legged partridge experiment was designed to assess viral dynamics, tissue distribution, and neuropathological features in a natural host with endemic infection, rather than to model a naïve infection. To complement this limitation, we established an infection model in SPF chickens, which allowed controlled experimental inoculation in ParPgV-naïve birds and provided an independent system to evaluate susceptibility, replication, and host responses under defined baseline conditions.

R2: *The reported dip in viremia at 10 dpi followed by a rise at 14 dpi in Figure 4c may suggest biphasic kinetics, but given the low n and variability, stochastic fluctuation cannot be ruled out.*

A: We thank the reviewer for their careful examination of Figure 4c. We would like to clarify that viral RNA in blood was measured only at weekly intervals (0, 7, 14, and 21 dpi), as stated in the figure caption. No measurements were performed at 4 or 10 dpi. The absence of coloration at these time points in the heatmap reflects missing data rather than low or fluctuating viral loads.

R2: *Figure 5a further illustrates these limitations: the histological panels lack animal identifiers, viral load data, and inoculum information, making it difficult to contextualize the findings. Moreover, no uninfected control tissues are shown for comparison, and the observed lesions, congestion and lymphoid infiltration, are, at least to my eyes, mild. As presented, the pathology does not compellingly demonstrate virus-specific effects and leaves the overall infection model underpowered to support strong conclusions.*

A: We thank the reviewer for this detailed comment. Figure 5 is intended to provide representative examples of histopathological changes observed in experimentally

infected birds and is not meant to serve as a standalone quantitative comparison. The images shown correspond to animals at 21 dpi with confirmed ParPgV RNA detection in neural tissues, as detailed in the associated molecular analyses. In line with standard pathological practice, uninfected control tissues were not included as side-by-side panels, as histopathological evaluation was descriptive and interpreted in the context of baseline avian neuroanatomy, experimental infection status, and viral localization by RNAscope and immunohistochemistry. We agree that the lesions observed are mild to moderate in nature, and we have revised the text and figure legend accordingly to avoid overstatement (line 647). Importantly, our conclusions regarding neurotropism and pathogenic potential are not based on histology alone but on viral replication, and tissue distribution following natural or experimental infection.

R2: Minor comments

R2: *The severity of neuropathology may be overstated. While the figure legend for Fig. 5d describes "severe inflammatory infiltrates" and "neuronal degeneration and necrosis," the presented cerebellar histology appears relatively mild, with only modest infiltrates and no overt evidence of widespread neuronal loss or Purkinje cell degeneration. Without quantitative scoring, additional stains, or side-by-side mock controls, these interpretations seem somewhat speculative. Although I am not a trained pathologist, the images do not strongly support the claim of severe neuroinflammation or neurodegeneration as written.*

A: We thank the reviewer for this constructive observation. The description of lesion severity was based on the examiner's professional assessment of the neuropathological changes. However, we agree that the term "severe" may appear subjective in the absence of quantitative scoring. Accordingly, we have modified the wording in the figure legend from "severe inflammatory infiltrates" to "clear inflammatory infiltrates," (line 647) which more accurately reflects the qualitative histopathological findings while avoiding potential overstatement. Despite this, the presence of inflammatory infiltrates and neuronal degeneration remains evident in the presented micrographs.

R2: *Line 436 and elsewhere, Flaviviridae should be italicised.*

A: We thank the reviewer for noting this formatting detail. The taxonomic family name Flaviviridae has been italicized throughout the manuscript where appropriate.

R2: *Figure 2: The depiction of the 5′ and 3′ UTRs as rounded rectangles extending beyond the genome creates the misleading impression that these regions extend past the actual UTR boundaries. Both ParPgV-A and ParPgV-C should be shown with properly annotated UTRs, as done in Supplementary Figure 2, for consistency and accuracy. Furthermore, the figure currently only marks the signalase cleavage site; no putative polyprotein cleavage sites are annotated, and the "gene boundaries" appear to*

represent protein domains rather than predicted mature proteins. Inclusion of predicted cleavage sites and clarification of what the boundaries represent would strengthen the figure. Finally, the overall nucleotide identity between ParPgV-A and ParPgV-C should be reported in the main text to contextualize their relationship.

A: We thank reviewer for pointing out the inconsistencies between the Fig. 2 and the Supplementary Fig. 3 (former Supplementrary Fig. 2), which could lead to misunderstanding. We have now amended the suggested changes in the Fig. 2, which now appears aligned to the Supplementary Fig. 3. Furthermore, we also annotated the putative polyprotein protease cleavage sites in the Fig. 2. Details on the predicted mature proteins have already initially been given in the Table 2. To clarify the Table 2 we have added following sentence to its legend:" Predicted mature proteins within the viral polyprotein were identified using HMMER Hmmscan search against the Pfam protein database (HmmerWeb version 2.43) and by comparison to the Goose Pegivirus-1 polyprotein." Finally, to conceptualize the relationship between ParPgV-A and ParPgV-C we have added to the results section of the manuscript the information on the overall nucleotide identity between these two strains as well as the amino acid identity over the entire viral polyprotein (please see lines 491-492).

R2: *The manuscript does not clarify whether the red-legged partridge in Video S1 was confirmed to be ParPgV-positive; without direct testing, it should be explicitly described as anecdotal and not used as supporting evidence.*

A: We thank the reviewer for this comment and agree that clarification is warranted. The video was recorded during the 2021 outbreak in one of the affected red-legged partridge flocks to document the characteristic neurological signs observed at that time. The individual bird shown was not tested for ParPgV infection, as the causative agent had not yet been identified; therefore, the video is presented as illustrative field documentation rather than as direct experimental evidence of infection.

R2: *Video S2 is unconvincing; multiple uncontrolled factors influence activity levels, and the footage lacks side-by-side cage status, standardized conditions or even standard video composition. Removal of Video S2 is recommended. Also there is a difference in enrichment between the control and inoculated cages in S2.*

A: We thank the reviewer for the thoughtful comment and acknowledge that Video S2 was not recorded under standardized experimental filming conditions. The footage was intended solely as qualitative documentation of the transient reduction in flight activity observed in inoculated grey partridges at 4 dpi, consistent with the mild and short-lived clinical signs reported in the text. The footage was collected opportunistically for illustration and does not represent a standardized behavioural test. We believe that retaining this video as part of the Supplementary Material enhances transparency by allowing readers to assess the behavioural observations directly and form their own judgment regarding the described clinical signs.

R2: *Supplementary Figure 2b is unclear regarding the 3′ end of the ParPgV-C genome—please clarify whether this represents an extended 3′ UTR or an unannotated coding region, it looks like a white box is over the 3'UTR and cutting off the letters from UTR.*

A: We thank the reviewer for noticing this mistake that was due to the error in the formatting of the file containing Supplementary figures. We now corrected this error.

R2: *The legend of Supplementary Figure 2 states that ParPgV-C has longer 5′ and 3′ UTRs than ParPgV-A, yet the general and strain-specific PCR primer positions are depicted identically between the two genomes, please clarify how primer binding sites can align if UTR lengths and genome coordinates differ. Additionally, Supplementary Table 1 does not indicate which primers are "variant-specific" or how they correspond to the labelled PCRs in Supplementary Figure 2; please clarify which primer pairs were used for each screening assay and ensure coordinate consistency across the figure and table.*

A: We appreciate the reviewer's comment. Because ParPgV-C contains longer 5′- and 3′-UTRs, slight discrepancies occur in the genomic coordinates between the two ParPgV strains, even when referring to homologous regions. This was indeed considered in Supplementary Table 1, which lists all primers used in the study. Each nucleotide used in PCR-based assays is identified by both name and a corresponding number in the column "Primer/Probe name". These number identifiers are used in the PCR schemes for both genomes shown in Supplementary Fig. 3.
The table also clearly specifies the name of the assay (column: "Assay"), together with oligonucleotides used including name and number ("column: "Primer/Probe name"), their sequences (column: "Sequence 5'-3')), and their precise positions in each of the two ParPgV genomes (column: "Position on ParPgV genome"). In Supplementary Figure 3, primer positions are adapted to the schematic representation of each genome. Although the two schematic representations appear similar despite the ~230 bp difference in genome lengths, the positions of all oligonucleotides are depicted correctly.

R2: *Where the infecting strain is known, it should be indicated in the relevant figure legends to aid interpretation. For example, Fig. 5 could be revised to read: "Fig. 5. Viral load dynamics, histopathological lesions, and MRI findings in red-legged partridges (Alectoris rufa) experimentally inoculated with ParPgV-C."*

A: We thank the reviewer's remark. This was now modified in Figures 4, 5, and 7.

R2: *Figures 4c and 5a both present viral gRNA quantitation but differ in visual representation and interpretation. While Figure 5a and its legend describe a peak at 14 dpi, the violin plots show narrower distributions and potentially lower variability at*

that time point than at 7 or 10 dpi. In contrast, the heatmap in Figure 4c suggests viremia peaks earlier, particularly at 7 dpi. To reconcile these differences, the authors should consider plotting individual data points or using consistent visual formats (e.g. line plots) across figures to more accurately reflect temporal dynamics. It is also unclear what threshold defines a negative result (e.g. Ct > 38 or <1 copy/reaction), and whether this was applied consistently—several samples appear negative in Figure 4c, yet all blood samples in Figure 5a are plotted as positive, including those with <1 genome copy per reaction. This should be clarified.

A: We thank the reviewer for this detailed and constructive comment. We agree that clear visualization and consistent interpretation of viral RNA kinetics are essential. However, Figures 4c and 5a represent data derived from distinct experimental settings and were therefore intentionally presented using different formats. Figure 4c depicts viral RNA levels in blood from the grey partridge experiment, in which only five birds were inoculated. In this experiment, viral RNA was detected sporadically, with a high proportion of negative samples across time points. For this reason, a heatmap representation was chosen as the most appropriate way to visualize presence/absence patterns and relative signal intensity in a small dataset with many non-detects. Direct comparison of temporal dynamics between Figure 4c and Figure 5a is therefore not informative, as the figures represent different host species, different sample sizes, and different experimental contexts. Figure 5a, in contrast, summarizes viral load data from the red-legged partridge experiment with a larger group size and more consistent detection of viral RNA. In response to the reviewer's suggestion, Figure 5a has now been updated to boxplots with overlaid individual jittered data points, allowing direct visualization of variability, distribution, and individual trajectories over time.

With regard to apparent differences in peak timing, we note that Figure 5a describes trends in viral load distribution rather than a formal kinetic peak, and variability between individual birds remains substantial. No claim of precise peak viremia timing is intended.

The definition of qRT-PCR positivity and the assay threshold are described in the Materials and Methods (lines 208–210). In Figure 5a, individual data points below this threshold are plotted at the lower detection limit for visualization purposes.

R2: *The phylogenetic analysis lacks key methodological details, including the GBlocks trimming parameters and the specific protein substitution model used in RAxML, while likely appropriate, must be explicitly reported. Full version information for all software tools (MUSCLE, GBlocks, RAxML, MegAlign Pro) should also be included.*

A: We thank the reviewer for this suggestion. The methodological details of the phylogenetic analysis, including the software tools used, have now been added to the Materials and Methods section under "Genome and phylogenetic analysis" (lines 176–195).

R2: *The qPCR field data in Figure 3 are difficult to interpret due to the use of ΔCt (38 – Ct) without reporting actual Ct values, viral copy numbers, or a clearly defined limit of detection (LOD). As a result, many samples appear "positive" despite likely being near or below the assay's detection threshold. This issue is particularly problematic given the presence of two distinct ParPgV strains, yet no attempt is made to differentiate or quantify them separately in these field samples. The authors should report the Ct threshold for positivity directly on the figure, clarify whether any pools contained negative samples, and ideally provide the proportion of positive pools per farm and sample type. Inclusion of raw Ct values or viral copy numbers, alongside explicit designation of the LOD and clarification of strain composition, would substantially improve interoperability.*

A: We thank the reviewer for this detailed and constructive comment. In response, we have substantially revised the presentation of the field qRT-PCR data to improve clarity, transparency, and interoperability. Specifically, Figure 3 has been updated by (1) redefining the y-axis as ΔCt (40 – Ct), (2) explicitly indicating the limit of detection (LOD) and Ct positivity threshold directly on the figure, and (3) presenting the data as box plots with overlaid individual jittered values, allowing visual assessment of both viral load distribution and the number of positive versus negative pools per farm and sample type.

To further address concerns regarding raw data availability and strain composition, we have added a new Supplementary Table 3 containing the individual Ct values for all tested pools, including results for the general ParPgV assay as well as strain-specific assays for ParPgV-A and ParPgV-C, together with the corresponding ΔCt values.

R2: *The authors should deposit the raw sequencing data from the inoculum and sample datasets on the NCBI SRA database. Additionally, sequencing of the other inocula used in the red-legged partridge and SPF chicken experiments should be performed to confirm the absence of mixed infections.*

A: We have now submitted the raw sequencing data from all field samples that were used as inocula into the NCBI SRA database, under the BioProject PRJNA1314175. Data under accessions SAMN50922524 and SAMN50922527 corresponds to the inoculum used for animal experiment in grey partridges, whereas data with accessions SAMN50922526 and SAMN50922529 is from the inoculum used in animal experiments performed in red-legged partridges and SPF chickens.

R2: *For both the IHC/RNAscope brain staining (Fig. 7) and the cerebellar MRI images (Fig. 8), it is unclear at what time point post-infection the samples were taken.*

A: We thank the reviewer for this comment. The timing of tissue collection for histopathology, immunohistochemistry (IHC), and RNAscope analyses is described in

the Materials and Methods (lines 334–335), where it is stated that birds were euthanized at predetermined time points (Table 1) and that organ samples were collected for histopathological and molecular analyses. IHC and RNAscope were performed on FFPE tissues originally processed for H&E staining, with no separate or additional sampling specifically for these assays. Regarding MRI images, the new Fig. 6 now has that information clearly.

R2: *The use of recombinant E2 protein to generate the polyclonal antibody for IHC is appropriate, but the inclusion of serological testing would strengthen the study. Serology could clarify baseline infection status and provide independent evidence of immune recognition. Moreover, testing infected partridge serum against the recombinant E2 protein would help validate antibody specificity.*

A: We thank the reviewer for this valuable suggestion. In response, we have now incorporated ELISA-based serological analyses into the Materials and Methods and Results sections to assess antibody responses against ParPgV. These data provide additional evidence of immune recognition and help clarify baseline infection status in the investigated birds. In addition, we expanded the characterization of the polyclonal anti-ParPgV antibody in Supplementary Fig. 2 by testing its reactivity against E proteins from closely related flaviviruses, thereby further validating its specificity. We believe these additions substantially strengthen the study and address the reviewer's concerns.

R2: *The manuscript does not define the Ct threshold used to classify samples as positive, negative, or inconclusive for negative-strand RNA, nor does it describe how these classifications were validated. Without this information, the designations in Supplementary Table 3 are difficult to interpret and may overstate evidence of active replication. The authors should clearly state the Ct cutoffs, include appropriate controls, and clarify how inconclusive results were determined.*

A: Detection of negative-strand RNA was performed using conventional reverse transcriptase PCR (RT-PCR); therefore, cycle threshold (Ct) values cannot be provided. To validate the assay, a 260-bp fragment of the NS4A region was amplified and cloned into the pCR®4-TOPO vector. Positive- and negative-strand RNA controls were generated *in vitro* from the T3 and T7 promoter sites of the NS4A-pCR®4-TOPO construct and were used as positive and negative assay controls, respectively. This procedure was already described in the original Materials and Methods section. To improve clarity, we have now expanded and clarified this description in the revised manuscript (see "Detection of ParPgV negative-strand RNA by RT-PCR," lines 379–394).

R2: *The manuscript does not specify which strain the antigen was derived from, whether it cross-reacts with both genotypes, or the E2 amino acid identity between strains, limiting interpretation of the IHC results and broader applicability of the antibody.*

A: As stated in the manuscript, the antigen was derived from the ParPgV-A strain and comprises a partial E2 protein fragment (aa 414–570 in the polyprotein). Since the amino acid identity between the two ParPgV strains within this region is 95.5%, we are confident that the polyclonal antibody raised against this antigen also recognizes the ParPgV-C strain. In our animal experiments with red-legged partridges and chickens, the inoculum consisted exclusively of the ParPgV-C strain. Therefore, the positive IHC results from the red-legged partridges demonstrate that this antibody indeed cross-reacts with both strains.

R2: *While the histopathological findings are convincing and supported by viral load data, the MRI results are difficult to interpret. With only two infected and one control bird imaged per time point, and no quantification of cerebellar volume or signal intensity, the evidence for atrophy remains anecdotal. The authors should clarify whether the phenotype was consistent across both infected individuals and distinguishable from natural variation. Including all MRI scans as a supplementary figure and attempting even limited quantification, despite the small sample size, would help strengthen this aspect of the study.*

A: We thank the reviewer for this thoughtful and important comment. We agree that the MRI component of the study is limited by the small number of imaged birds per time point and by the absence of quantitative volumetric or signal intensity analyses. These constraints reflect both ethical and practical limitations associated with MRI acquisition in game bird species. Accordingly, MRI findings were intended as supportive, qualitative evidence rather than as standalone proof of neuropathology.
To strengthen this aspect of the study, we have now included the complete MRI dataset for all imaged birds and time points in a new Figure (Fig. 6). All MRI examinations were reviewed and interpreted in a blinded manner by a European Board-certified veterinary radiologist to ensure consistency and diagnostic accuracy. Notably, asymmetric cerebellar (and, to a lesser extent, cerebral) atrophy was observed consistently in ParPgV-inoculated birds at later time points and was distinguishable from control animals, supporting a reproducible phenotype rather than incidental anatomical variation.
We acknowledge that inflammatory CNS disease does not invariably result in overt MRI signal changes, and that encephalitis can be present despite normal MRI findings, as documented in veterinary and human medicine. In this context, the absence of additional parenchymal signal alterations outside the cerebellar region does not preclude neuropathology but suggests a localized process. We further note that post-contrast imaging was not performed, as MRI acquisition occurred during euthanasia

and additional contrast-enhanced sequences would have required separate ethical approval.

Reviewer #3 (R3) (Remarks to the Author):

"*The manuscript Neurotropism and Encephalitis of a Novel Pegivirus with Experimental Evidence Across Avian Species by Matos et al., describes a novel avian pegivirus (partridge pegivirus, ParPgV) associated with field outbreaks of encephalitis in red-legged partridges (Alectoris rufa) in France. The authors have employed multiple methods to identify the new pegivirus (such as, next-generation sequencing, full-length genomic characterization), and its associated neurotropism (such as, histopathology and electron microscopy), and further performed confirmatory experimental infections in multiple avian species.*

I found the research plan expertly executed, and the manuscript flawless. The outcome is original and significant for the field. I do not have further remarks for the authors. I recommend the manuscript for publication, and I compliment the authors for their robust contribution to the scientific field."

A: We sincerely thank the reviewer for the positive assessment of our work and the kind remarks. We greatly appreciate the encouraging feedback and are pleased that the reviewer found the study design, methodology, and conclusions to be of high quality and significance to the field.

We thank the reviewers for their careful evaluation of our manuscript and for the constructive and insightful comments provided. We have addressed all points raised in detail below and have revised the manuscript accordingly.

Reviewer comments

Reviewer #2 (Remarks to the Author):

"*The authors have addressed the majority of my comments satisfactorily, and the revised manuscript is substantially improved as a result. In particular, the additional clarification of inoculum composition, the inclusion of strain-specific qPCR data, and the improved presentation of methodological and experimental details have considerably strengthened the study.*

Some limitations in the study design remain, particularly regarding host background infection status and the interpretability of some in vivo findings, and these should continue to be acknowledged clearly in the manuscript. However, I do not consider these remaining issues sufficient to preclude publication in revised form."

A: We sincerely thank the reviewer for the careful evaluation of the revised manuscript and for the positive assessment. We greatly appreciate the recognition that the revisions, including clarification of inoculum composition, incorporation of strain-specific qPCR analyses, and improvements in methodological and experimental descriptions, have strengthened the study.

We fully agree that certain limitations remain, particularly regarding host background infection status and the interpretation of some *in vivo* findings. These aspects are clearly and explicitly acknowledged in the manuscript, particularly in the Discussion section, where we outline the constraints of the experimental design and the implications for causal interpretation (lines 449-451, 466-468).

We are grateful for the reviewer's constructive feedback throughout the review process, which has significantly improved the clarity and rigor of our work.

Reviewer #4 (Remarks to the Author):

"*The authors have largely addressed the concerns of reviewer 1 by revising the language around epidemiological findings. However, I agree that the authors have not sufficiently recapitulated clinical presentation of disease observed at partridge farms in their in vivo (but not naive) model to directly link encephalitis to infection with ParPgV as the MRI shows volume loss rather than swelling of the brain tissue. I agree that the title should be revised from "of a" to "associated with".*

A: We thank the reviewer for this important observation. We acknowledge that MRI findings in the present study demonstrated reduced brain volume rather than swelling. However, the absence of increased brain volume or mass effect does not exclude encephalitis, as imaging features are highly dependent on the stage and severity of inflammation. It is likely that imaging in our *in vivo* model was performed after the acute inflammatory phase, capturing post-encephalitic sequelae such as malacia and secondary cerebral atrophy, which reflect neuronal loss and parenchymal resorption rather than edema. Similar patterns have been described in other avian viral infections; for example, cerebral atrophy and malacia were reported in raptors infected with West Nile virus (Gamino *et al*., 2013), supporting the concept that viral encephalitis may manifest as parenchymal loss at later stages.

We also thank the reviewer for this suggestion regarding the title. We agree that the original phrasing ("of a pegivirus") could be interpreted as going too far regarding the causal relationship. In line with this feedback, we have revised the title to: "Encephalitis Associated with a Neurotropic Pegivirus Supported by Experimental Infection Across Avian Species." This modification more accurately reflects the associative nature of the findings while remaining consistent with the experimental evidence presented.
